# Beyond the X Factor: Relevance of Sex Hormones in NAFLD Pathophysiology

**DOI:** 10.3390/cells10092502

**Published:** 2021-09-21

**Authors:** Sara Della Torre

**Affiliations:** Department of Pharmaceutical Sciences, University of Milan, Via Balzaretti 9, 20133 Milan, Italy; sara.dellatorre@unimi.it

**Keywords:** liver, metabolism, NAFLD, sex and gender differences, sexual dimorphism, sex hormones, estrogens, androgens, estrogen receptors, androgen receptor

## Abstract

Non-alcoholic fatty liver disease (NAFLD) is a major health issue worldwide, being frequently associated with obesity, unbalanced dietary regimens, and reduced physical activity. Despite their greater adiposity and reduced physical activity, women show a lower risk of developing NAFLD in comparison to men, likely a consequence of a sex-specific regulation of liver metabolism. In the liver, sex differences in the uptake, synthesis, oxidation, deposition, and mobilization of lipids, as well as in the regulation of inflammation, are associated with differences in NAFLD prevalence and progression between men and women. Given the major role of sex hormones in driving hepatic sexual dimorphism, this review will focus on the role of sex hormones and their signaling in the regulation of hepatic metabolism and in the molecular mechanisms triggering NAFLD development and progression.

## 1. Introduction

The liver is a key organ in energy metabolism, playing a central role in the processing, partitioning, and metabolism of macronutrients, in the transport and storage of nutrient-derived metabolites, in lipid homeostasis, and in protein and amino acid (AA) metabolism [1]. In addition, the liver is relevant in several other physiological processes, including immune response, detoxification, and breakdown of xenobiotic compounds [1]. In several of these biological processes, the liver of males and females differ, accounting for the highest degree of sexual dimorphism, with 72% of the hepatic genes expressed in a sexually differentiated manner [2]. The underlined, and partly underestimated, liver sexual dimorphism can explain sex differences in susceptibility, progression, and outcomes of liver diseases such as non-alcoholic fatty liver disease (NAFLD) as well as the associated extra-hepatic diseases [3,4,5,6]. The aim of this review is to summarize the role of sex-specific factors in the sexual dimorphism characterizing liver physiology and the development, progression, and degeneration of NAFLD in the two sexes. In particular, this review focuses on the relevance of sex hormones and their nuclear receptors in the regulation of biological processes participating in NAFLD pathophysiology.

## 2. The Sex-Specific Regulation of the Healthy Liver Metabolism

The liver plays a central role in the regulation of energy metabolism and in the adaptation to nutrient availability [1,7]. After feeding, sugars (mainly glucose) released by intestine in the bloodstream reach the liver via the portal vein, where glucose is converted and stored in the form of glycogen [8]. Once the glycogen stores are fulfilled, the excess of glucose is broken down through glycolysis to produce acetyl-CoA, starting from which the liver synthetizes fatty acids (FA) [8]. The stimulation of FA synthesis is greater in the liver of males than females, due also to a sex-specific response to circulating hormones, mainly insulin and leptin, which increase after feeding is, in turn, different between the two sexes [9].

Starting from cholesterol (CH), the liver synthetizes biliary acids (BA), that are then secreted into the small intestine to emulsify and promote the absorption of dietary lipids that reach the bloodstream as components of chylomicrons [10]. The synthesis and composition of BA are sexually dimorphic in both mice and humans [11]. Even though they display larger total BA pool, female mice catabolize less CH via BA and excrete less fecal BA compared to males [11]. In female mice, the excretion of fecal BA changes during the progression of the estrous cycle [12].

FA are extracted from chylomicron remnants through the action of lipoprotein lipase (LPL), which activity is higher in women and female rodents than in males [13,14].

Through several transport proteins, mainly cluster of differentiation 36 (CD36) and fatty acid transport proteins (FATPs), the FA derived from chylomicron remnants are then transported into hepatocytes, where they are assembled into triglycerides (TG). Female liver clears FA from plasma more rapidly than male liver, due to an enhanced rate of transport through the plasma membrane of hepatocytes, which reflects a greater expression and affinity of CD36 and FATPs for FA [15,16]. In comparison to males, females show a greater metabolic flexibility and are able to limit hepatic FA uptake and TG synthesis when lipids are provided in excess with the diet [17].

TG are then packaged with very-low density lipoprotein (VLDL) particles and secreted into the bloodstream to provide lipid substrates for the body. At the hepatic level, women have an accelerated production of VLDL-TG and a lower secretion rate of VLDL particles than men [18,19]. In women, the enrichment of VLDL in TG [18,20,21] facilitates the hydrolysis of VLDL-TG by LPL and enhances the removal of VLDL-TG from the circulation [22], thus resulting in lower circulating VLDL-TG [19]. Under an excess of dietary lipids or under obesogenic-like conditions, the enhanced delivery of FA to the liver increases VLDL production and VLDL-TG clearance rates in females, thus contributing to export TG from the liver, prevent hepatic lipid deposition, and lower circulating VLDL-TG levels [17,20].

Circulating TG can reach other organs such as the adipose tissue, where they can be stored as a long-term energy source, or the skeletal muscle, where they can be used as an energy source. With respect to men who tend to accrue more adipose tissue in the visceral area, women accumulate more adipose tissue in the gluteo-femoral area, that show a reduced lipolytic activity accounting for a lower delivery of free fatty acids (FFA) to the liver [20]. Women also display an enhanced FA clearance by skeletal muscle [20].

The liver preserves glucose homeostasis through the regulation of glycogen breakdown/synthesis, glycolysis, and gluconeogenesis (GNG), through which it produces glucose starting from AA, lactate, and glycerol [8]. In comparison to men, women are more sensitive to insulin action in the liver and suppress hepatic glucose production (HGP, which accounts for 90% of endogenous glucose production) to a greater extent at low insulin concentrations [23]. The livers of males and females differ for their metabolic adaptation to fasting and refeeding conditions [9] and for the regulation of glucose homeostasis under physiological conditions [24], a feature that might partly explain the gender-specific susceptibility to type 2 diabetes (T2D) [25,26].

Under long-term fasting, the liver can oxidize FA as an internal energy source and use FA for the synthesis of ketones (acetoacetate and β-hydroxybutyrate) to provide energy to other organs [7]. In the liver, the oxidation of FA (FAO) and the synthesis of ketones are sexually dimorphic, occurring to a greater extent in women than men [27].

The liver is relevant for the homeostasis of CH, which is required for the assembly of cellular membranes, the maintenance of membrane fluidity, and the synthesis of steroid hormones, including sex hormones [1]. Upon feeding, CH can be absorbed from the intestine or synthetized de novo at the hepatic level. Systemic CH homeostasis is achieved through the complex regulation of secretion, uptake, and efflux of CH by the liver, which has in charge most of the synthesis, assembly, and remodeling of lipoproteins [28]. The livers of males and females show a different regulation of CH and lipoprotein metabolism, resulting in sex-differences in the levels of circulating CH and in the concentration, distribution, and size of lipoprotein particles, which may contribute to the sex-specific prevalence of atherogenesis and cardiovascular diseases (CVDs) [14,19].

During both, fasted and fed conditions, pre-menopausal women display higher circulating levels of high-density lipoprotein (HDL) than age-matched men [19]. Women display greater HDL apolipoprotein A-I and apolipoprotein A-II synthesis rate, resulting in higher circulating levels of HDL [19,29,30]. Furthermore, women have larger, CH-enriched HDL particles than men [29,31]. Despite the lack of clinical studies investigating the potential sexual dimorphism in CH kinetics in the various lipoprotein fractions, pre-clinical studies support the role of sex hormones in mediating CH and lipoprotein metabolism in a sexually dimorphic manner [12,32]. In particular, the female liver contributes to sex differences in lipoprotein remodeling and in CH homeostasis through the synthesis of a class of HDL particles able to better promote CH efflux [12].

The liver is a major site of protein metabolism and turnover, and it is responsible for the synthesis and secretion of ~90% of circulating proteins, including acute-phase proteins, growth factors, hepatokines, and numerous other peptides involved in the systemic regulation [33,34,35,36]. Among them, albumin, that contributes for the 55% of the total plasma proteins, acts as a carrier for several molecules, including lipids and hormones. Notably, the synthesis rate of albumin is greater in males than females [37]. The liver is also the main tissue responsible for the production of sex hormone-binding globulin (SHBG), a glycoprotein that binds sex steroids with high affinity and specificity [38]. The liver has a high capacity to breakdown proteins and metabolize hepatic AA to provide energy to the hepatocytes and glucose to the dependent organs in times of extended fasting. A recent study has demonstrated that the livers of male and female mice differ for their ability to catabolize the hepatic AA under a short-term fasting [39].

Given its central position in the body, its high degree of vascularization, its structure with highly permeable fenestrated endothelia, the liver represents a key immune tissue. Under physiological conditions, being constantly exposed to several foreign but harmless molecules derived from nutrients or resident microbiota, the liver’s immune status is immunotolerant [40,41]. However, when exposed to molecules with an inflammatory potential, the liver mounts a rapid and robust immune response by activating Kupffer cells (KCs, the resident macrophages) which, in turn, can recruit macrophages from the periphery. In the liver, homeostatic inflammation is tightly regulated by mechanisms acting to prevent accidental immune activation against otherwise harmless antigens as well as to resolve inflammation in order to avoid excessive inflammation and pathological consequences [40,41]. Although scarcely investigated under physiological conditions, male and female livers differ for the regulation of hepatic immune response, which may account for the sex-specific prevalence of inflammatory liver diseases [42,43,44].

Sex dimorphic expression of hepatic enzymes active in the metabolism of drugs, steroids, and environmental chemicals, especially cytochromes P450 (CYPs) and sulfotransferases [45,46,47], might account for the well-known sex differences in detoxification and drug metabolism and, therefore, for the differences in pharmacokinetics and pharmacodynamics between the two sexes [42].

## 3. The Sex-Factors Involved in the Hepatic Sexual Dimorphism

In mammal liver, the high degree of sexual dimorphism has been mostly ascribed to growth hormone (GH) and its sex-specific temporal pattern of pituitary secretion, which is highly pulsatile in males and more continuous in females [48,49]. GH regulates the sex-specific expression of a large number of hepatic genes, including CYPs, various plasma and urinary proteins, and several receptors and signaling molecules involved in a broad range of physiological processes, including the lipid metabolism [47,49,50,51].

GH dimorphic regulation of hepatic gene expression occurs mainly through the transcription factor signal transducer and activator of transcription 5b (STAT5b) [52,53], hepatocyte nuclear factors 3β, 4α and 6 (HNF3β, HNF4α, HNF6) [54,55] and their signaling cross-talk [56,57,58]. GH carries out its sexual differentiating action also through the sex-specific regulation of DNA methylation and chromatin structure [49,53,59,60,61,62].

The hepatic responsiveness to GH changes during development and is dynamic during adult life, leading the liver to adapt its functions to the needs of the organism throughout life [53,63,64,65].

The relevance of GH pattern in the hepatic sexual dimorphism has been proved in a series of experiments performed in male mice treated with continuous GH infusion to reproduce the GH pattern typical of females [59,66]. Persistent GH stimulation “feminizes” the temporal profile of liver STAT5 activity, leading to changes in chromatin states and to the activation of sex-specific transcriptional networks, with the repression of most of the male-biased genes and the induction of specific female-biased genes [59]. However, the fact that several highly female-specific genes show weak or no feminization [59] suggests that GH pattern alone cannot account for the sexual differentiation of the liver.

**Figure 1:**
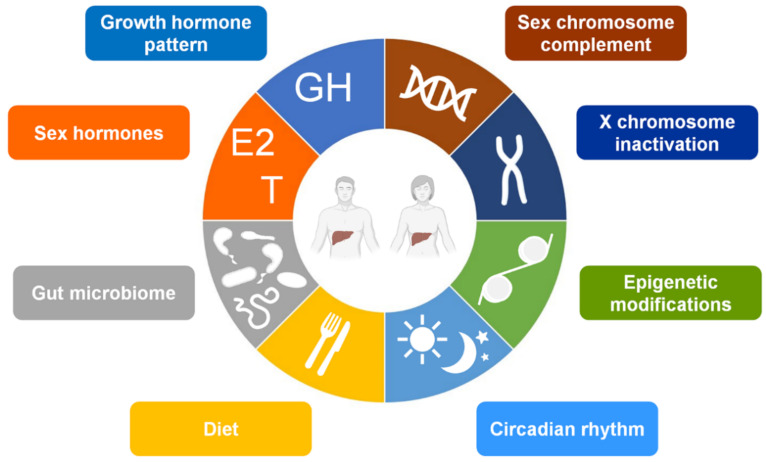
Main factors contributing to the hepatic sexual dimorphism. Abbreviations: E2, 17β-estradiol; GH, growth hormone; T, testosterone.

In addition to GH activity, indeed, several other factors can contribute to hepatic sexual dimorphism, including genetic and epigenetic factors, sex hormones, diet, circadian rhythm, and gut microbiota [39,60,62,67,68,69,70,71,72,73,74,75,76] (Figure 1). However, differently from other tissues where sex-chromosome complement action has a predominant role in the sex dimorphic gene expression, sex hormones have major effect on driving sex-biased gene expression in the liver [77,78]. According to this view, this review addresses the effects of sex hormones and their receptors in the sex-specific regulation of healthy liver and provides insight into relevance and mechanisms of sex hormones and their signaling in NAFLD pathophysiology.

In spite of the still-limited knowledge of the entity of hepatic sexual dimorphism under physio-pathological conditions [75], several evidences indicate that estrogens and their receptors recover a key role in the hepatic sexual dimorphism as well as in the sex differentiation of the liver [17,39,79,80,81]. Estrogens can contribute to the hepatic sexual dimorphism directly, by acting through their receptors [17,39,82,83,84] and indirectly, by regulating GH action [76,85,86]. The contribution of estrogen signaling to hepatic sexual dimorphism likely arises from the different metabolic costs of reproduction for the two sexes, with female mammals having acquired and perfected through evolution a higher metabolic flexibility to adapt the hepatic metabolism to nutrient availability and to the changeable energy needs of the different reproductive stages [39,75,87,88]. At peri-natal level, testosterone-derived estrogen programs the hepatic metabolism of males [39], a mechanism that resembles the estrogen-induced “organizational effects” observed in the brain [89,90] and that can further contribute to hepatic sexual dimorphism.

Androgen signaling can contribute to the sex-based hepatic phenotype in a direct or indirect fashion, by acting on GH dependent pathways [62,76,77,91], or by regulating the accessibility of DNA to several transcription factors through chromatin remodeling [92,93].

Sex differences in the liver can be modulated during lifetime according to the hormonal status, to the levels of estrogens and androgens as well as to their *ratio*, which changes with aging, especially in women after menopause, when the lack of estrogens makes closer male and female liver phenotypes [94].

## 4. Estrogen Signaling in the Healthy Liver

*Estrogens.* The naturally occurring estrogens are 17β-estradiol (E2), estrone (E1), estriol (E3), and estetrol (E4) [95,96]. E2 is the predominant and the most potent estrogen during reproductive years, from the menarche to menopause [97]; in pre-menopausal women, it is primarily synthesized by the theca and granulosa cells of the ovaries. After menopause, E1, which is synthesized in adipose tissue from adrenal dehydroepiandrosterone, becomes the primary form of estrogen in the body [97]. E3 is a weak estrogen that is produced in large quantities by the placenta [97] and plays an important role during pregnancy. In humans, E4 is produced during pregnancy exclusively by the fetal liver [98].

Estrogen synthesis starts with CH uptake by steroidogenic cells and occurs through a long biosynthetic pathway proceeding from CH to androstenedione and then to testosterone that, in the final step, is converted into E2 and E1 by the aromatase enzyme (CYP19A1). Besides the ovaries, CYP19A1 activity has been detected also in other tissues, including skeletal muscle, fat, nervous tissue, and testis, thus suggesting that estrogens play an important role also for male physiopathology, even though E2 concentration is approximately five times lower in men than in pre-menopausal women [97]. In males, the aromatase-dependent conversion of testosterone into estrogen exerts a key role in the sexual differentiation of the liver [39]. Notably, in rodents, aromatase has been found to be expressed and active in the gastric mucosa cells that secrete E2 in the portail vein [99,100], thus leading E2 to reach the liver and exert its regulatory action.

In women, E2 levels raise with puberty and oscillate during the reproductive lifespan [97]. During the reproductive cycle, E2 levels increase significantly from the early follicular phase to the late luteal follicular phase, and further on to the luteal phase. In the four-day long estrous cycle of female mice, E2 levels are highest at *Proestrus* and lowest at *Estrus/Metestrus* [12]. With menopause or ovariectomy (OVX), serum E2 drops to levels found in males [97]; E1, mostly derived from the aromatase-dependent conversion of testosterone occurring in peripheral tissues, especially in the adipose tissue [101,102], becomes the most prevalent estrogen in post-menopausal women.

*Estrogen receptors.* Estrogens exert their action mainly through the nuclear receptors ERα (estrogen receptor α) and ERβ (estrogen receptor β), but also through the membrane-associated ERα and ERβ variants, and the membrane-bound receptor G protein-coupled ER (GPER) [103,104,105]. In the mouse liver, estrogens act mainly through ERα, the most expressed isoform at the hepatic level, whereas ERβ and GPER show a very low expression [94], mostly limited to specific hepatic cell subtypes [3]. With respect to male mice, the hepatic content of ERα mRNA is four-six-fold on average higher in the liver of females [12], a feature that can further contribute to a sex-specific regulation of liver metabolism [39]. In female liver, the expression [12] and the transcriptional activity of liver ERα [81,106] oscillate according to estrogen levels, leading the female liver to adapt the hepatic metabolism to the energy requirements of each reproductive stage [79]. In mice, hepatic ERα expression and activity increase along development and reach their highest expression soon after sexual maturation to decrease with aging/OVX, likely a consequence of a reduced stimulation by the low hormone levels [12,39].

**Figure 2:**
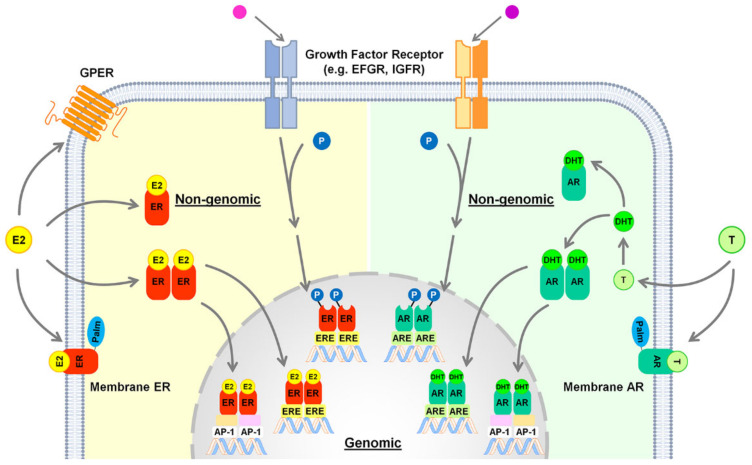
Potential mechanisms of action through which estrogen and androgen receptors can exert their action in hepatocyte cells. Abbreviations: AP-1, activator protein-1; AR, androgen receptor; ARE, androgen responsive element; DHT, dihydrotestosterone; E2, 17β-estradiol; EGFR, epidermal growth factor receptor; ER, estrogen receptor; ERE, estrogen responsive element; GPER, G protein-coupled estrogen receptor; IGFR, insulin-like growth factor receptor; P, phosphorylation; Palm, palmitoylation; T, testosterone.

Once activated by its ligand, ERα dimerizes and binds to estrogen response element (ERE) in the promoters of target genes [103]; as an alternative to the “classical” mechanism, ERα may indirectly bind the DNA through the tethering with other transcription factors, such as activator protein-1 (AP-1) [107] (Figure 2). In addition to these nuclear actions, ERα can exploit its action through faster, non-genomic effects (Figure 2), that involve intracellular second messenger systems, such as protein kinase A (PKA), protein kinase C, and mitogen-activated protein kinase (MAPK)/extracellular signal-regulated protein kinase (ERK) [108]. Estrogens can exert their role also through the palmitoylated, membrane-associated ERα [104,109] and through GPER [104,105] (Figure 2).

ERα is the isoform most expressed in the hepatocytes, the most abundant cell type accounting for 80% of liver mass [3,110,111,112,113], and in KCs [114], where it drives macrophage polarization and regulates cytokine production [115,116,117]. By converse, ERα is not expressed in the hepatic stellate cells (HSCs) [118], where estrogens seem to act mainly through ERβ [119] and GPER [120]. Cholangiocytes—the epithelial cells participating in bile production and secretion and, to a less extent than hepatocytes, in the liver development and regeneration [121,122]—express both, ERα and ERβ [123,124].

*Estrogens signaling, glucose homeostasis and insulin sensitivity.* In both, males and females, estrogen signaling improves glucose tolerance and insulin sensitivity, and may partly account for the sex-specific regulation of glucose homeostasis, as well as for the sex dimorphic prevalence of T2D [25,26,125,126,127]. In comparison to men, pre-menopausal women show higher glucose tolerance, enhanced glucose effectiveness (which is the ability of glucose to promote its own disposal in an insulin-dependent manner) and greater insulin sensitivity [25]. Although the underlined mechanisms are still unclear, sex differences in glucose homeostasis rely, at least in part, on the estrogen signaling. In fact, estrogen deficiency predisposes post-menopausal women to dysglycemia and to impaired hepatic insulin clearance, which can be improved by estrogen administration, thus reducing the risk of developing T2D [128,129,130]. The type and timing of menopause can further impact on glucose homeostasis: in fact, with respect to natural menopause, early menopause, and surgical menopause by OVX further increase the risk of T2D [129]. In female mice, OVX increases hepatic insulin resistance [131]. In male mice and in men, estrogen deficiency due to defects in the aromatase gene leads to insulin resistance, glucose intolerance, and increased risk of developing T2D, which can be improved by estrogen therapy [127,132].

Although most of estrogen effects derive from the promotion of glucose uptake in skeletal muscle and adipose tissue [127], estrogen signaling is relevant for glucose metabolism also at the hepatic level, where it modulates glucose uptake and production. The estrogen-dependent regulation of glucose uptake may occur through the inhibition of GLUT2 (glucose transporter 2, the main glucose transporter in hepatocytes of rodents and humans), which results, indeed, over-expressed in the liver of OVX rodents [133]. Estrogens suppress HGP by reducing GNG, and increase glycogen synthesis and storage, thus lowering circulating glucose level [125,134]. At mechanistic level, estrogens suppress GNG and improve insulin sensitivity acting via FOXO1 (forkhead transcription factor 1), a transcription factor with a pivotal role in HGP [135].

According to its expression in the liver, ERα but not ERβ is believed to account for most actions of estrogens on glucose homeostasis *in vivo*. In fact, total knockout of ERα (ERαKO) male and female mice develop insulin resistance and glucose intolerance, whereas total knockout of ERβ (ERβKO) mice exhibit normal glucose tolerance [125,136].

More recently, a study demonstrates that nuclear and membrane ERα differently contribute to insulin secretion and action in male and female mice, pointing to another level of sex dimorphic regulation of glucose homeostasis [137]. The lack of nuclear ERα signaling strongly impacts on glucose homeostasis, while the lack of membrane ERα leads to mild hepatic insulin resistance and glucose intolerance. Both, male and female mice lacking nuclear ERα exhibit fasting and feeding hyperglycemia and glucose intolerance. However, differently from males, the lack of nuclear ERα alters the central control of insulin sensitivity in females, which display hyperinsulinemia and insulin resistance due to unrestrained hepatic GNG. Differently from females, the lack of nuclear ERα impairs the central regulation of insulin secretion in male mice, which show impaired glucose-stimulated insulin secretion [137].

In spite of the role of estrogen signaling in liver glucose homeostasis, the specific relevance of hepatic ERα in the regulation of liver glucose metabolism has been questioned by studies performed in liver-specific ERα deficient (LERKO) mice leading to conflicting results. In fact, some studies indicate that hepatic ERα may have a specific role in the regulation of glucose homeostasis and insulin sensitivity in both males and females [80,138] while others advocate against it [139].

*Estrogen signaling and hepatic lipid metabolism.* Estrogen signaling is relevant in both males and females for the regulation of hepatic lipid metabolism, as demonstrated by several pre-clinical and clinical studies [12,17,80,81,83,132,136,140,141].

In women, the lack of estrogens due to menopause leads to altered liver lipid metabolism, which may trigger the development of hepatic steatosis/NAFLD [3,79,83]. Similarly, women with altered estrogen signaling, such as occurs in PCOS (poly-cystic ovary syndrome), display impaired liver metabolism and greater risk of developing NAFLD [79,142]. Regardless of energy intake, OVX in female mice leads to dysregulation of lipid metabolism and to liver fat accumulation, which is reversed by hormonal replacement therapy (HRT) [12,81,131,143].

In the liver of females under physiological conditions, estrogen signaling inhibits the expression of genes involved in de novo lipogenesis (DNL) such as *Srebp-1c* (sterol regulatory element binding transcription factor 1), *Fasn* (fatty acid synthase), *Scd1* (stearoyl-CoA desaturase 1), *Elovl6* (ELOVL fatty acid elongase 6), and promotes FAO, all mechanisms that are lost in OVX mice [81,144,145]. During the mouse estrous cycle, the oscillation of estrogen levels influences the hepatic expression of genes relevant for FA and CH metabolism in the liver [12,81], pointing to the relevance of maintaining such an oscillation to limit fat deposition in the female liver [81,106].

Men with aromatase deficiency show altered liver lipid profile and hepatic steatosis, which can be improved by estrogen therapy [146]. In the aromatase knockout (ArKO) mouse model of estrogen deficiency, hepatic steatosis occurs after six months of age, following the development of hepatic glucose intolerance, and can be reverted by estrogen treatment [147]. Although ArKO mice of both sexes display increased adiposity, glucose intolerance and insulin resistance [132], only ArKO males but not females show impaired lipid and lipoprotein metabolism and develop hepatic steatosis [132,148].

At the mechanicistic level, estrogens regulate liver lipid metabolism mostly acting through ERα, which is the predominant ER subtype in both, male and female hepatocytes [3,39,94]. Male and female ERαKO mice develop hepatic steatosis as a consequence of the increased expression of genes involved in DNL (e.g., *Srebp-1c*, *Scd1*) and decreased expression of genes involved in lipid transport [125,136].

The liver-specific ablation of ERα in LERKO female mice leads to increased DNL, altered hepatic expression of genes involved in lipid uptake and reverse CH transport, and increased hepatic lipid content [12,81,84], all changes that are mostly recapitulated in liver-targeted knockdown of ERα [149,150]. Under physiological conditions, when estrogen levels are high, hepatic ERα is responsible for the synthesis of a class of small HDL able to efficiently promote the CH efflux [12], a mechanism that could have been settled to spare CH for reproductive functions (i.e., steroidogenesis) [6,75] and that can explain the lower incidence of atherogenesis and CVDs in pre-menopausal women with respect to men and post-menopausal women [151,152,153].

Although less investigated, also in males the lack of ERα leads to increased hepatic lipid droplets and TG content as a result of enhanced DNL (*Acaca*, acetyl-CoA carboxylase α; *Fasn*) and reduced hepatic lipid export (ApoB, apolipoprotein B; MTTP, microsomal triglyceride transfer protein) [80,138].

More recently, a study demonstrates that hepatic ERα signaling is relevant for the regulation of hepatic metabolism of both males and females, even though through different mechanisms and with different metabolic consequences under unbalanced dietary regimens [17]. The sex-specific role exerted by hepatic ERα could be a consequence of the female-specific ability to regulate the hepatic metabolism to reproductive needs, which might strongly account for the sex differences observed in liver lipid metabolism [6,14,82].

*Estrogen signaling and hepatic amino acid metabolism.* In addition to estrogens, dietary AA activate hepatic ERα to support the progression of the estrous cycle [87]. Under long-term 40% calorie restriction, the significant reduction of hepatic ERα activation results to be associated with the progressive block of estrous cycle progression [87]. A diet enriched in AA has been shown to preserve the progression of the estrous cycle in control but not LERKO females, at least in the early phase of the calorie restricted regimen [87], suggesting that the tight connection between reproductive and liver metabolic functions strongly relies on the activation of hepatic ERα by dietary AA.

The hepatic metabolism of dietary AA is significantly different in the two sexes, likely a consequence of the female-specific role of hepatic ERα in modulating hepatic metabolism according to the energy needs of reproductive functions, a regulation that should have been reached its maximum degree of complexity in female mammals [75,79]. Under short-term fasting, liver ERα promotes in females, but not in males, the catabolism of the hepatic AA pool to preserve lipid synthesis, thus providing lipids for steroidogenesis and ensuring the progression of the estrous cycle [39]. When fed with high-fat diet (HFD), differently from males, female mice preserve the hepatic AA homeostasis [17]. Interestingly, the development of hepatic steatosis in males is associated with a decrease in the hepatic pool of AA, resembling the correlation between low AA content and hepatic steatosis observed in obese humans [154]. The female-specific ability to preserve AA homeostasis under HFD depends on the hepatic ERα, as it is lost in LERKO females. Notably, the metabolism of branched-chain amino acids (BCAA: leucine, isoleucine, valine) is the pathway most affected by HFD regimen in the liver of male mice and LERKO females that develop hepatic steatosis [17], in agreement with studies reporting a role of estrogen signaling in the control of BCAA metabolism in female rodents [155].

*Estrogens and GH signaling.* Estrogens can modulate GH actions in the liver by acting both, at the central and peripheral level [156]. Estrogens stimulates GH secretion and influences the levels of IGF-1 (insulin-like growth factor-1, the main mediator of the anabolic action of GH) depending on the route of administration. In fact, only oral but not parenteral route of administration exposes the liver to pharmacological levels of estrogens able to inhibit IGF-1 production [91,156]. At a peripheral level, estrogens attenuate GH action by affecting the expression of its receptor and its signaling through the JAK/STAT (janus kinase/signal transducer and activator of transcription) signaling pathway [156].

## 5. Androgen Signaling in the Healthy Liver

*Androgens.* Androgens play an important role in the metabolic homeostasis and reproductive health of both sexes. In males, androgens are synthesized mainly by the testes and, to a lower extent, by the adrenal glands [157]. Besides testosterone—the major androgen in males -, other androgens are dihydrotestosterone (DHT), dehydroepiandrosterone (DHEA), androstenedione (A4), androstenediol (A5), and androsterone. In both sexes, androgen levels steeply rise in puberty [158] and gradually decline with aging [159]. Although androgens are commonly thought as male hormones, females can produce androgens, even though to a much lower level than males [157]. In females, androgens are produced by the ovaries (testosterone), by the adrenal glands (androgen precursors like DHEA and A4), and, during pregnancy, by the placenta (testosterone). In older women, the decrease of circulating androgens seems due to aging more than to post-menopausal condition [160]. In women, some pathological conditions such as PCOS, obesity, and endocrinopathies, such as Cushing disease, are associated with high levels of androgens [161].

*Androgen receptors.* Once activated by its ligands (testosterone and DHT), the androgen receptor (AR) enters the nucleus and modulates gene expression by binding as a homodimer to specific DNA known as androgen response element (ARE) motifs in its target genes [162] (Figure 2). Like ERs, AR can regulate the transcriptional activation of many other genes by binding to DNA regions other than ARE, through the recruitment of other transcription factors such as AP-1, nuclear factor-κB (NF-κB), and sex-determining region Y [163] (Figure 2). Androgen effects can be mediated by AR also via non-genomic signaling pathways, including the MAPK (mitogen-activated protein kinase) and the PI3K/AKT (phosphatidylinositol-3-kinase/AKT) pathways, or by the membrane-associated AR as well as by other plasma membrane associated receptors, including the epidermal growth factor receptor (EGFR) [164,165,166] (Figure 2). In rodents, the hepatic expression of AR is several-fold higher in males than in females and changes with age, increasing with puberty and gradually declining with aging [140]. AR is expressed in hepatocytes, but not in other hepatic cell types such as KCs and liver sinusoidal endothelial cells [167].

*Androgen signaling, glucose homeostasis and insulin sensitivity.* Androgen signaling plays a relevant role in glucose homeostasis as suggested by several reports showing that altered androgen levels increase hepatic glucose output, induce hyperglycemia, and lead to high risk of developing T2D in both males and females [168]. However, androgens display sex-specific effects on glucose metabolism in the two sexes, as low levels in males (i.e., due to androgen depletion/deficiency) and high levels in females (as occurs in PCOS women) increase the risk of T2D [168,169,170].

In males under physiological conditions, testosterone, acting via AR, increases insulin receptor (IR) expression [171,172] and glycogen synthesis [173], and decreases glucose uptake by inhibiting the transcription and translation of GLUT-2 [174]. Testosterone can improve glucose homeostasis also by repressing hepatic GNG through the interaction with FOXO1 [175]. The effects of androgens on insulin signaling are not clear, since some studies reported that testosterone supplementation can improve insulin sensitivity [171,172], while others have demonstrated that testosterone supplementation worsens hepatic insulin resistance in a male mouse model of T2D [175]. The inhibition of 5α-reductase 2, which converts testosterone into DHT, leads to hyperglycaemia, elevated hepatic glycogen storage, altered GLUT2, IR, and AR expression in the liver of males, pointing to a specific role of androgen/AR signaling in the regulation of glucose metabolism in males [176]. According to this view are studies reporting that the lack of hepatic AR (LARKO) exacerbates diet-induced impairment of glucose metabolism in male mice [177].

Differently from males, in females, testosterone impairs hepatic glucose metabolism, resulting into a sex-specific risk of developing T2D [168]. In post-menopausal women, increased androgen activity is associated with impaired glucose tolerance [178,179]. In PCOS women, the excess of androgens impairs hepatic glucose metabolism by decreasing insulin-stimulated glucose uptake and glycogen synthesis, thus predisposing PCOS women to insulin resistance [169]. In a lean mouse model of hyperandrogenemia, females show impaired glucose tolerance and disrupted glucose metabolism [180]. At a mechanistic level, low-dose of DHT in females leads to increased hepatic AR binding to PI3K, resulting in reduced PI3K activity, decreased *p*-AKT and lower insulin action, which can be restored by AR inhibition [180]. In the liver of these females, DHT promotes GNG via direct transcriptional regulation of gluconeogenic enzymes such as *Pck1* (phosphoenolpyruvate carboxykinase 1) and *G6pc* (glucose-6-phosphatase) [180].

Beyond DHT-mediated effects, testosterone can regulate glucose metabolism and insulin sensitivity once converted into estrogen by the CYP19A1 enzyme. According to this view, glucose tolerance and insulin sensitivity can be restored by testosterone but not by the non-aromatizable androgen DHT in orchidectomized (ORX) male mice [181] as well as in men with central obesity [182]. Male ArKO mice as well as men with *CYP19A1* defects show insulin resistance and hyperglycemia [146,147,183]. Notably, the lack of aromatase affects in a sex-specific fashion the glucose metabolism in ArKO mice, since, in addition to glucose intolerance, males but not females display pyruvate intolerance and insulin resistance [184].

Low androgen levels may impair glucose metabolism and insulin signaling in the liver also through extra-hepatic actions that, in turn, worsen hepatic glucose production and insulin resistance [185]. Androgens can impact on the regulation of glucose metabolism also through epigenetic and transgenerational mechanisms. In fact, androgen treatment in the last days of pregnancy decreases glucose tolerance in female offspring [186]. In adult females, DHT treatment leads to impaired glucose tolerance, increased insulin resistance, reduced AKT and PI3K, and increases the hepatic content of enzymes involved in GNG (e.g., *Foxo1*, *Pck1*, *G6pc*) [180].

*Androgen signaling and hepatic lipid metabolism.* Many studies have shown that altered androgen levels impair lipid metabolism and cause hepatic steatosis in both humans and rodents [140,171,186,187,188,189], pointing to a role of androgen signaling in the regulation of lipid metabolism in the liver.

In rodent males, ORX leads to insulin resistance at both hepatic (increased HGP) and extra-hepatic (reduced skeletal muscle glucose uptake) level, resulting in hepatic steatosis, that can be reversed by testosterone administration [190]. In males, androgen signaling regulates the hepatic lipid metabolism by inhibiting the expression of genes involved in DNL and lipid storage (such as *Srebp-1c*; *Acaca*; *Ppar*𝛾, peroxisome proliferator activated receptor γ) and by promoting FAO through the up-regulation of PPAR𝛼 (peroxisome proliferator activated receptor α) signaling [188].

Although beneficial in males, androgen excess impairs hepatic lipid metabolism and promotes hepatic steatosis in androgen-treated female rodents [189,191], resembling what observed in PCOS women [169]. In female mice, DHT increases SCAP (sterol regulatory element-binding protein cleavage-activating protein) protein expression and SCAP-SREBP1 binding, thus favoring the nuclear localization of SREBP1 and promoting DNL [189].

The relevance of AR in mediating androgen effects seems to be supported by studies showing that the lack of 5α-reductase, which is responsible for the conversion of testosterone in DHT, results in an impaired regulation of liver lipid metabolism in male mice [187,192]. However, testosterone replacement reduces HFD-induced lipid deposition in the liver of testicular feminized (Tfm) mice, which are characterized by very low testosterone levels as well as by non-functional AR [188], suggesting that testosterone effects can be partially independent of AR. According to this view, ArKO male mice, that are unable to convert testosterone into estrogens, develop hepatic steatosis, which can be reversed by E2 treatment [147]. In addition, differently from total AR knockout mice, which develop liver steatosis and insulin resistance in both sexes, the lack of AR in the liver accounts for sex-specific metabolic consequences. In fact, only LARKO males but not females show impaired DNL and FAO and develop hepatic steatosis and insulin resistance [173,177], suggesting that hepatic AR may play a more critical role in liver lipid metabolism of males than females.

Although less investigated, androgen signaling has a role also in the regulation of CH and lipoprotein metabolism. Low androgen levels in aging men are associated with increased serum CH and LDL levels, and decreased HDL level, that can be normalized by testosterone replacement. In ORX male mice, DHT treatment increases CH uptake from circulating HDL by up-regulating the hepatic expression of scavenger receptor class B member 1 (SR-1B), reduces LDL secretion and suppress CH removal by decreasing cholesterol 7𝛼-hydroxylase (CYP7A1) [173]. Although beneficial in counteracting dyslipidemia, chronic androgen replacement may, indeed, lead to CH accumulation in the liver [171]. Male but not female ArKO mice display abnormal lipoprotein metabolism that is associated with high testosterone levels and increased AR mRNA and protein in the liver [148]. In Tfm mice, non-functional AR and low testosterone levels lead to decreased expression of genes involved in CH metabolism (such as *Abca1*, ATP binding cassette subfamily A member 1 and *ApoE*, apolipoprotein E), through the repression of Liver X receptor α (LXRα) [193], pointing to a cross-talk between AR and LXRα in the regulation of hepatic CH homeostasis [194] that has not been clarified.

All together, these findings suggest that low androgen levels in males and high androgen levels in females negatively affect the regulation of liver lipid metabolism. In comparison to females, androgen signaling has a greater impact in male liver, where androgen effects can be—at least partially—mediated by the conversion of testosterone into estrogens, which can act through ERs.

*Androgen signaling and hepatic amino acid metabolism.* Testosterone limits protein catabolism, by reducing the rate of protein oxidation and by promoting AA availability for reutilization and for muscle protein synthesis and regeneration through mechanisms that have been shown to be mediated by the liver [195,196]. In fact, oral delivery of low dose of unconjugated testosterone, which can reach the liver only, reduces whole-body protein loss in men as well as in post-menopausal women [195,197]. Testosterone prevents protein catabolism by inhibiting the hepatic urea cycle [196], thus reducing hepatic loss of nitrogen and sparing AA for protein synthesis. Such a mechanism could explain, at least in part, the strong correlation between the reduced levels of testosterone and the increased incidence of sarcopenia in aging men [198] and could partly account for sex differences in muscle wasting [199].

Testosterone may have a role also in the programming of AA metabolism, as suggested by studies reporting impaired BCAA metabolism in neonatal testosterone-treated female rats [200]. Beyond this evidence, the relevance of hepatic AR in the liver-mediated regulation of AA metabolism has not been clarified.

*Androgens and GH signaling.* In males, testosterone exerts its effects also by stimulating, at the hypothalamic level, the secretion of GH [201,202], which, in turn, drives hepatic IGF-1 production [203], a process that, however, relies on the conversion of testosterone into estradiol [204]. In fact, treatment with the not-aromatizable androgen DHT does not stimulate GH secretion [205] and the inhibition of ER signaling abrogates the GH-stimulatory effect of testosterone [204]. Notably, men with aromatase deficiency display reduced GH secretion and impaired GH response, which cannot be restored by estrogen replacement [206].

In addition to GH secretion, androgens increase tissue responsiveness to GH [185], pointing to a synergistic interaction between the two signaling pathways [207,208]. In GH-replete individuals, liver-targeted testosterone administration triggers the interaction with GH and promotes the GH-mediated synthesis of IGF-1 [195,197]. At the hepatic level, testosterone may enhance the action of GH also by increasing the expression of the GH receptor [209]. Accordingly, LARKO male mice display a marked reduction of circulating IGF-1 levels [177]. In females, administration of testosterone at neonatal stage contributes to the remodeling of the GH axis and modifies the expression and the methylation status of female-predominant GH-dependent genes in the liver [61,210], a defeminization which may compromise liver physiology [211].

## 6. NAFLD, a Sex-Based Liver Disease

NAFLD is a spectrum of liver diseases, ranging from hepatic steatosis due to excessive TG accumulation within hepatocytes, to non-alcoholic steatohepatitis (NASH), fibrosis, cirrhosis, and hepatocellular carcinoma (HCC) [212,213,214]. NAFLD has emerged as the most common form of chronic liver disease and represents an increasing public health issue due to its emerging association with several extra-hepatic diseases, especially CVDs [215,216,217,218].

In the liver of NAFLD patients, TG accumulation results from the imbalance between enhanced lipid uptake and DNL [219,220,221] and reduced lipid oxidation and secretion [214,222]. The impaired mitochondrial FAO and the enhanced peroxisomal β-oxidation and microsomal ω-oxidation leads to the generation of reactive oxidative species (ROS) within the hepatocytes, to chronic oxidative stress, and to endoplasmic reticulum stress [219,222,223,224,225].

According to the multiple-hit theory [212], lipotoxic lipid intermediates and oxidative stress mediate the activation of JNK (c-Jun N-terminal kinase) and NF-κB signaling pathways, resulting in the increased production of pro-inflammatory cytokines (i.e., IL-6, interleukin 6; TNFα, tumor necrosis factor α) by hepatocytes and non-parenchymal cells, including KCs and HSCs [226,227,228,229,230]. Persistent activation of the JNK and NF-κB pathways worsens insulin resistance and leads to a chronic inflammatory state and to the activation of apoptosis, fostering liver injury and NAFLD progression toward NASH [231,232,233,234,235]. The pro-inflammatory response of these cells, in turn, promotes the recruitment of other immune cells, mainly macrophages [115]. Immune cells release pro-inflammatory cytokines which intensify the inflammatory process, hindering the liver to properly regenerate, an ability essential for the maintenance of the hepatic metabolic functions [236]. In these conditions, the process of liver regeneration, which mostly relies on the proliferative capacity of existing mature hepatocytes, becomes inefficient due to altered interaction with the liver-resident immune cells [237,238]. As a consequence of liver injury and impaired tissue regeneration, HSCs become activated and differentiate into myofibroblasts, leading to extracellular matrix deposition, fibrosis, and liver degeneration [239,240,241]. Under these conditions, the liver act as both a target of and a contributor to systemic chronic inflammation, boosting the progression of NAFLD toward more harmful conditions such as NASH, fibrosis, cirrhosis, and HCC [214,242,243]. Together with the unbalanced metabolism, the systemic inflammation contributes to the development of NAFLD-associated extra-hepatic diseases, such as atherosclerosis, CVDs, chronic kidney disease, osteoporosis, and inflammatory bowel disease [215,244,245,246,247,248,249,250].

**Figure 3:**
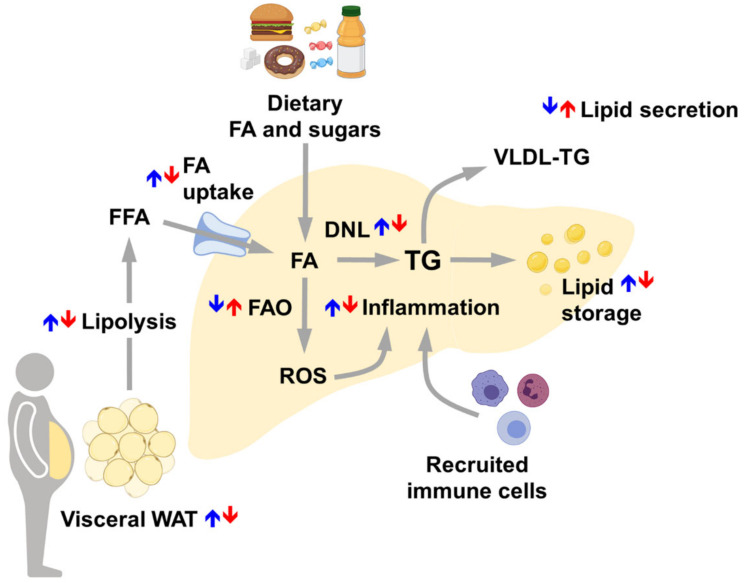
Main sex differences in the regulation of healthy liver accounting for sex differences in NAFLD susceptibility. Arrows represent the relative regulation between males (blue) and females (red). With respect to men, women display decreased visceral adipose tissue lipolysis, limited FA uptake and DNL, restrained lipid storage and inflammation and enhanced FAO and lipid secretion. Abbreviations: DNL, de novo lipogenesis; FA, fatty acids; FAO, fatty acids oxidation; FFA, free fatty acids; ROS, reactive oxygen species; TG, triglycerides; VLDL-TG, very-low density lipoproteins—triglycerides; WAT, white adipose tissue.

In comparison to men, women show a lower incidence of NAFLD, likely a consequence of the sex dimorphic regulation of the healthy liver which may account for a different susceptibility to NAFLD for the two sexes (Figure 3). However, after menopause, the prevalence of NAFLD becomes similar between the two sexes [5,42,79], owing to the protective effect of estrogens [3]. Differently from men who display an increasing prevalence of NAFLD during adulthood from young to middle-age, the prevalence of NAFLD in women rises after the age of 50 years, peaks at 60–69 years and declines after 70 years [251]. This temporal pattern suggests that NAFLD incidence in aging women relies more on the lack of estrogens than on aging *per se*, even though aging may exacerbate its progression [252,253,254]. Supporting this view, young women suffering of reproductive dysfunctions characterized by altered estrogen levels (such as PCOS, Turner Syndrome) as well as young, oophorectomized women show increased incidence of NAFLD with respect to young fertile women [79,255,256]. The relevance of estrogens in counteracting NAFLD in women is further supported by the fact that pre-menopausal, post-menopausal, and PCOS women with NAFLD exhibit a significantly lower concentration of serum 17β-estradiol with respect to their control counterparts [255] and that the risk of developing NAFLD is reduced in post-menopausal women taking HRT [251,257].

The full achievement of sexual differentiation seems to be relevant for sex-specific prevalence and features of NAFLD pathology. In fact, although NAFLD prevalence is higher in boys than in girls [258], sex differences in NAFLD are less relevant in the pediatric population than in adults. Notably, there is a strict association between earlier age at menarche and prevalence and features of NAFLD later in life [259,260,261,262].

## 7. Risk Factors Triggering NAFLD Development and Progression

**Figure 4:**
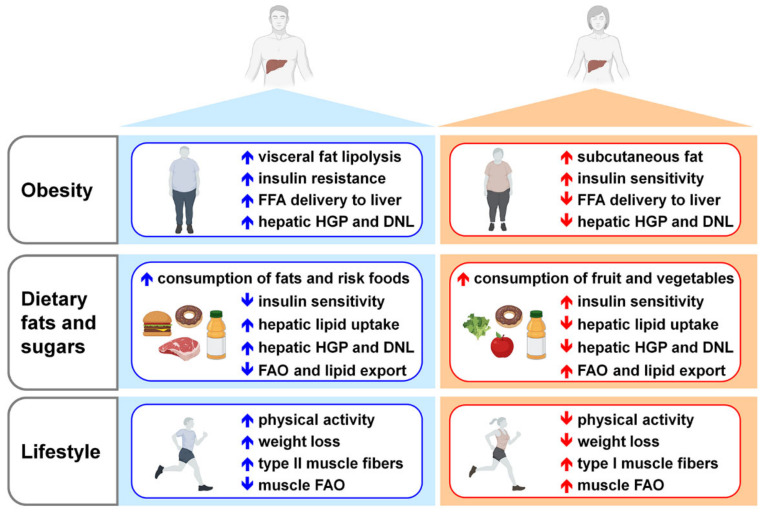
Main risk factors for NAFLD development in men and women. For both sexes, obesity, unbalanced dietary regimens and reduced physical activity represent risk factors for NAFLD development and progression. Arrows represent the relative regulation between males (blue) and females (red). Compared to men, obese pre-menopausal women display more subcutaneous than visceral fat and higher insulin sensitivity, leading to reduced adipose tissue lipolysis and FFA delivery to liver; women show limited HGP and DNL. Under an excess of dietary sugars and fats, with respect to men, women have higher insulin sensitivity, inhibit hepatic lipid uptake, HGP and DNL, and promote FAO and lipid export. Although generally women have a lower physical activity than men, when exercised, oxidize more fats due to the increased type I muscle fibers. Abbreviations: DNL, de novo lipogenesis; FAO, fatty acids oxidation; FFA, free fatty acids; HGP, hepatic glucose production.

Beyond genetic/epigenetic factors [263,264], several other factors might contribute to NAFLD development and progression, especially obesity, diet, and lifestyle (Figure 4).

With respect to general population showing a 25–30% NAFLD incidence, NAFLD prevalence rises to 90% in morbidly obese patients [218,265]. In obese patients, NAFLD is mostly due to the increased uptake mediated by CD36 of the FFA derived from the enhanced lipolysis of adipose tissue [266,267,268]. Dietary FA (~15%) and enhanced DNL (~25%) from ingested carbohydrates, that reach to a greater extent the liver due to the insulin resistance of the muscle, contribute, indeed, to a less extent to liver fat accumulation in obese people [221,266,269]. In obese patients, the high flux of lipids and carbohydrates toward the liver promotes lipotoxicity and glucotoxicity, which, in turn, lead to mitochondrial defects, endoplasmic reticulum stress, oxidative stress, and to the activation of a pro-inflammatory response, favoring NAFLD progression and liver injury [212,222,233,270,271]. Under obesogenic-like conditions, the impaired regulation of metabolic process and signaling pathways in other tissues showing a strong interplay with the liver, including adipose tissue, skeletal muscle, and gut-microbiota, can further negatively affect liver homeostasis and boost NAFLD progression [272,273,274,275,276]. In particular, the unbalanced secretion of adipokines (i.e., adiponectin and leptin) and the enhanced infiltration and activation of immune cells in the adipose tissue of obese people promote insulin resistance and hepatic steatosis, and foster hepatic inflammation [275]. Although the higher prevalence of obesity among female population, women result to be more protected than men from obesity associated NAFLD, at least until menopause [277], suggesting a pivotal role exerted by estrogens in counteracting its development [6,79].

Despite the strong association between obesity and NAFLD, recent studies report that ~40% of the global NAFLD population can be classified as non-obese and almost 1/5 as lean [278], suggesting that, beyond obesity, other factors might contribute to the burden of NAFLD nowadays. Independently of lean/fat mass, peripheral insulin resistance and hepatic insulin resistance are closely linked with NAFLD [279,280,281]. Under conditions of hepatic insulin resistance, insulin fails to suppress HGP, while keeps promoting DNL, leading to hyperglycemia, hypertriglyceridemia, and hepatic steatosis [282]. Notably, HGP strongly correlates with the extent of liver fat in NAFLD patients [283] and NAFLD is a common trait of insulin-resistant disorders such as T2DM and sarcopenia [284,285,286]. Possibly because of a sex-dimorphic regulation of glucose homeostasis [25], women show an improved glycemic control, a greater peripheral and hepatic insulin sensitivity and a reduced HPG with respect to men [26,287,288], all features contributing to a lower susceptibility to NAFLD.

Independently of energy intake, dietary habits may directly promote NAFLD, by modulating liver fat deposition and antioxidant activity and, indirectly, by affecting insulin sensitivity and the post-prandial lipid metabolism [289]. In particular, overconsumption of saturated fats and *trans*-fats and sugars (fructose, in particular) is considered the main nutritional mediator of NAFLD development [290,291,292,293]. Several human studies have reported that women have a higher consumption of fruit and vegetables and a lower consumption of meat and high-risk food (fats, processed meat, soft drinks) than men, contributing to sex differences in the risk of developing NAFLD [294,295,296,297].

Dietary regimens enriched in fats fuel the flux of FFA toward the liver, where they promote DNL and an enhanced oxidation, leading to lipotoxicity [298]. Dietary FA can also modulate the transcription of specific genes involved in lipid metabolism, thus influencing NAFLD pathogenesis [299,300]. Saturated and *trans* FA are particularly detrimental for hepatic health because they can alter the composition of plasma cell membrane, impairing cellular homeostasis and amplifying a pro-inflammatory response, which, in turn, boosts insulin resistance, fatty liver, and liver injury [298,301,302,303]. With respect to the male counterparts, women and female rodents show a lower propensity of developing hepatic steatosis/NAFLD under excess of dietary lipids, likely a consequence of reduced import of FA, limited synthesis, and storage of lipids within the liver and higher oxidation and secretion of FA [17,27]. Dietary FA can contribute to the sex-specific incidence of NAFLD also by changing the composition and the *ratio* of FA in liver plasma cell membrane in sex-specific manner [304]. Dietary FA may alter gut microbiota and interfere with the developmental programming of hepatic metabolism in a sexually dimorphic manner, leading to hepatic steatosis and liver inflammation [73,74].

The overconsumption of sugars is associated with increased incidence of hepatic steatosis and liver inflammation [305,306,307]. Fructose is particularly detrimental for hepatic health, since by-passes the rate-limiting step of glycolysis and promotes hepatic steatosis by stimulating hepatic DNL, inhibiting mitochondrial FAO, and inducing endoplasmic reticulum stress, oxidative stress, hepatocellular damage, and inflammation, which, in turn, further promotes an aberrant lipid metabolism [292,305,307,308,309,310,311]. Gut microbiota dysbiosis due to high fructose intake can also contribute to the development of insulin resistance, inflammation, and NAFLD [312]. The overconsumption of fructose leads to different consequences on liver health for the two sexes; indeed, being more responsive to fructose, males show higher hepatic postprandial DNL and higher prevalence of NAFLD compared to females, likely as direct consequence of the sex-specific modulation of glucose metabolism [25,313,314,315,316].

Although less investigated, dietary proteins and AA may also have a role in NAFLD etiology [289]. However, their effects on hepatic health are controversial, since some studies have indicated that high-protein diets can revert hepatic steatosis, while others have suggested that they can instead promote NAFLD development [317]. Although their causative or associative role has not yet clarified, BCAA, that account for 20% of total protein intake [318], seem to be beneficial for hepatic health, as they alleviate hepatic steatosis and liver injury and prevent hepatic fibrosis in a mouse model of NASH [319,320]. By contrast, elevated circulating BCAA and low hepatic content of BCAA are strongly associated with NAFLD, even in a sex-specific fashion [17,154,321,322]. Indeed, the low hepatic content of BCAA correlates with increased lipid deposition in the liver of male, but not female mice when fed with HFD [17]. Independently of BMI, insulin resistance and age, circulating BCAA levels display sex-dimorphic changes with increasing severity of NAFLD [322], suggesting that the sex-specific regulation of BCAA metabolism might have a key role in driving hepatic steatosis in a sex-specific fashion.

Inadequate physical activity and sedentary behavior are independent predictors for NAFLD development and, therefore, together with diet, exercise interventions are the first-line treatment option for the treatment of this pathology [323,324,325]. Increased physical activity reduces body weight and hepatic lipids, and improves glucose control, insulin sensitivity, and liver histology [325,326,327,328]. Although results from the limited clinical studies available are not consistent, in general men tend to lose more weight, especially fat mass from the visceral area, leading to greater metabolic benefits compared to women, who principally lose subcutaneous adiposity [20,329,330,331,332,333,334]. Indeed, while a 7-10% loss of body weight has been known to be sufficient to significantly ameliorate hepatic health in NAFLD male patients, a weight loss greater than 10% is necessary to obtain a similar improvement in NAFLD females [335].

Even in the absence of major weight loss, endurance exercise training reduces human liver fat by promoting mitochondrial biogenesis and increasing the capacity of hepatocytes to oxidize lipids [324,328,336]. Chronic exercise modifies the hepatic gene expression in a sex-specific manner, improving glucose tolerance and reducing hepatic insulin resistance, steatosis, fibrosis, and inflammation [337].

In response to exercise, the skeletal muscle produces and releases myokines, extracellular vesicles and several metabolites, that can influence the cross-talk between the skeletal muscle and other organs, including the liver [336,338]. Exercise-induced beneficial effects on the hepatic health can be mediated also by other organs, including the adipose tissue, where it increases mitochondrial biogenesis and the oxidative capacity, all adaptations that in mice but not humans are associated with a beige phenotype [339,340,341].

Although generally more sedentary, women display greater beneficial effects from increased physical activity compared to men, likely a consequence of the different composition of the skeletal muscle fibers and of the different lipid metabolism [342,343,344]. The skeletal muscle of women is enriched in type I fibers, which are characterized by higher content of intra-myocellular lipids, greater lipid oxidation, and higher insulin sensitivity with respect to men skeletal muscle, which expresses more type II fibers [345,346]. More recently, a study demonstrates that physical activity improves hepatic mitochondrial function and fat oxidation in a diet-induced model of hepatic steatosis in a sex-specific fashion [347].

## 8. NAFLD and Estrogen Signaling

**Figure 5:**
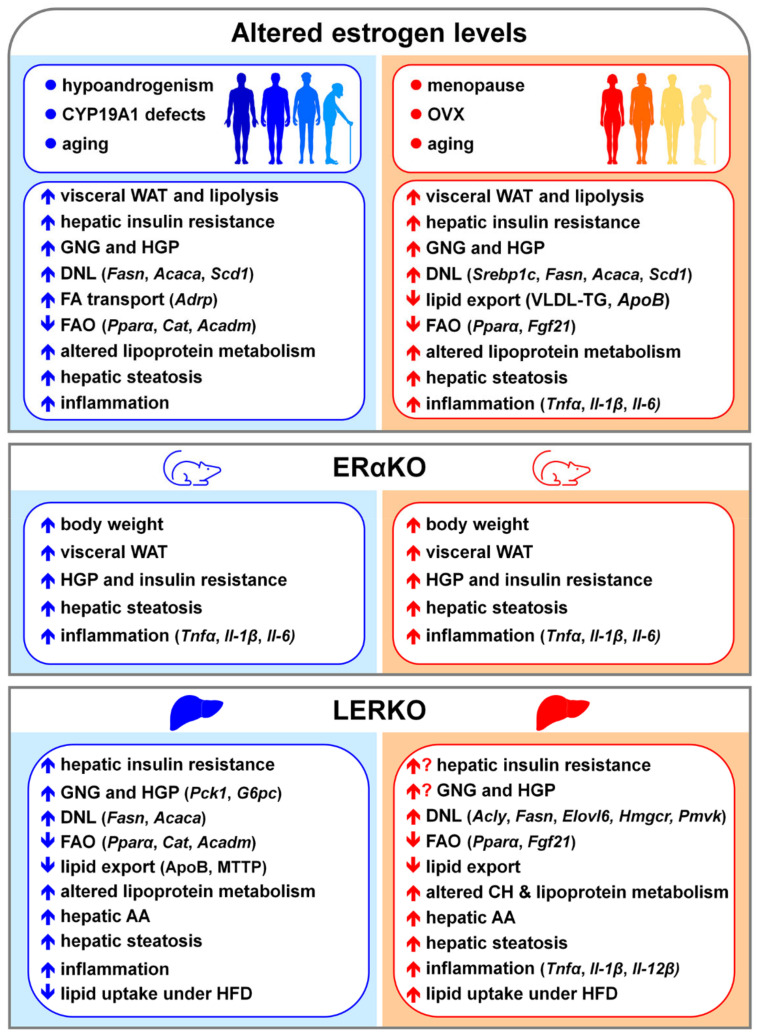
Consequences of altered estrogen signaling in the liver of males (left, in blue) and females (right, in red) favoring NAFLD development and progression. Abbreviations: AA, amino acids; *Acaca*, acetyl-CoA carboxylase α; *Acadm*, acyl-CoA dehydrogenase medium chain; *Acly*, ATP citrate lyase; *Adrp*, adipocyte differentiated regulatory protein; ApoB; apolipoprotein B; Cat, catalase; CH, cholesterol; CYP19A1, aromatase; DNL, de novo lipogenesis; *Elovl6*, ELOVL fatty acid elongase 6; ERαKO, total ERα knockout mice; FA, fatty acids; FAO, fatty acid oxidation; *Fasn*, fatty acid synthase; *Fgf21*, fibroblast growth factor 21; *G6pc*, glucose-6-phosphatase; GNG, gluconeogenesis; HFD, high fat diet; HGP, hepatic glucose production; *Hmgcr*, 3-hydroxy-3-methylglutaryl-CoA reductase; *Il-1β*, interleukin 1β; *Il-6*, interleukin 6; *Il-12β*, interleukin 12β; LERKO, liver ERα knockout mice; MTTP; microsomal triglyceride transfer protein; OVX, ovariectomy; *Pck1*, phosphoenolpyruvate carboxykinase 1; *Pmvk*, phosphomevalonate kinase; *Pparα*, peroxisome proliferator activated receptor α; *Scd1*, stearoyl-CoA desaturase 1; *Srebp1c*, sterol regulatory element binding Transcription factor 1; *Tnfα*, tumor necrosis factor α; VLDL-TG, very-low density lipoprotein-triglycerides; WAT, white adipose tissue.

The relevance of estrogen signaling in counteracting NAFLD has been outlined by several pre-clinical studies recapitulating the increased prevalence of NAFLD in estrogen deficient conditions (Figure 5), such as occurs in post-menopausal women and in men with mutations in the aromatase gene [79,146,348,349].

In OVX female mice, the lack of estrogens elicits hepatic insulin resistance, enhances DNL and FA import, and restrains FAO and lipid secretion, resulting in fatty liver [79,81,83,143]. In OVX females, estrogen replacement limits liver fat deposition by improving insulin sensitivity [135], inhibiting DNL [81,144], increasing hepatic VLDL-TG production, facilitating the VLDL-mediated export of lipids from the liver [83,350,351], and bolstering FAO by inducing PPARα and FGF21 (fibroblast growth factor 21) [144,352].

Although effective in reducing hepatic steatosis [81,143,144,351,352], the administration of constant amounts of estrogens as well as of SERMs (selective estrogen receptor modulators) partially restores a proper regulation of hepatic metabolism, likely as a consequence of the inability to reproduce the physiological, cyclic activation of hepatic ERα associated with estrous cycle progression in females [12,81,106,352]. Furthermore, depending on their route of delivery, estrogens differently impact on liver metabolism. Indeed, oral delivery of estradiol increases VLDL production and plasma TG, while transdermal estradiol reduces plasma TG by increasing the rate of VLDL-TG clearance without affecting VLDL-TG production [83,353,354].

In ArKO male mice, estrogen deficiency triggers liver fat deposition, which results from altered hepatic mitochondrial function, reduced expression of genes involved in FAO (*Cat*, catalase; *Acadm*, acyl-CoA dehydrogenase medium chain), and elevated expression of genes relevant for DNL (such as *Fasn, Acaca*, *Scd1*) and FA transport (such as *Adrp*, adipocyte differentiated regulatory protein) [132,141,147,355,356]. In ArKO males, estradiol treatment preserves hepatic mitochondrial function and partly rescues the regulation of lipid metabolism, thus retrieving mice from hepatic steatosis development [141,356]. Evidence suggests that estrogen effects in male liver can be also mediated by PPARα signaling. In fact, treatment of ArKO male mice with the PPARα agonist bezafibrate greatly limits hepatic steatosis [357]. In addition, PPARα knockout (PPARαKO) male but not female mice die with massive hepatic steatosis after treatment with etoxomir, an inhibitor of hepatocellular FA flux; in PPARαKO males, estradiol pre-treatment reduces etomoxir-induced mortality to 20% [358].

Furthermore, the exposure to endocrine disrupting chemicals (EDCs) predisposes both, males and females to increased risk of developing NAFLD by interfering with the physiological signaling of estrogens [359,360,361,362]. The exposure to EDCs in the early stages of life is particularly detrimental for both sexes since EDCs can interfere with the estrogen-dependent programming of hepatic metabolism and impair the full achievement of hepatic sexual differentiation [39,361,362]. Among EDCs, BPA (bisphenol A), MEPH (mono-2-ethylhexyl phthalate) and DEPH (di-2-ethylhexyl phthalate) are the EDCs which impact mostly on estrogen signaling [359]. At mechanistic level, EDCs interferes with estrogen signaling by competing with endogenous ligands for ER binding, thus altering lipid metabolic pathways and leading to liver fat accumulation. In particular, EDCs favor hepatic steatosis by increasing DNL and lipid uptake, by decreasing FAO, and by reducing the secretion of lipids in the form of VLDL particles and BA [359,360].

NAFLD progression and liver degeneration can be sustained by the propagation of inflammation, which is different between males and females [251,363,364], possibly as a consequence of the inhibitory control exerted by estrogen signaling over JNK and NF-κB signaling pathways [365,366,367,368]. In OVX females, the lack of estrogens fosters NAFLD development and progression by boosting pro-inflammatory response (e.g., TNFα, IL-1β and IL-6), and decreasing antioxidant defense and anti-inflammatory response (e.g., IL-10, interleukin 10), all changes that can be rescued or, at least, limited by estrogen therapy [369,370,371]. Prolonged estrogen deficiency boosts hepatic inflammation and worsens liver injury in OVX female mice fed with HFD as well as in post-menopausal women with NAFLD [372,373]. In the liver, the propagation or the resolution of inflammation mostly relies on the polarization abilities of KCs and of the recruited macrophages, which, once activated, undergo pro-inflammatory or anti-inflammatory and reparative phenotype, promoting or attenuating NAFLD progression, respectively [115,363,374,375]. Estrogens limit NAFLD progression by promoting the skewing of macrophages from a pro-inflammatory to an anti-inflammatory phenotype, thus accelerating the resolution of inflammation [363,376].

Exposure to EDCs can contribute to NAFLD progression by promoting the production of cytokines as well as other pro-inflammatory molecules, by inducing the polarization of KCs to a pro-inflammatory phenotype, by increasing hepatocyte proliferation and immune cell infiltration, and by favoring the transformation of HSCs to myofibroblast-like cells, thus impairing the balance between proliferation/apoptosis and, consequently, triggering liver damage and fibrosis development [359,360].

Impaired estrogen signaling in both, males and females can further increase the risk of developing NAFLD by worsening the negative metabolic effects associated with risk factors such as obesity, unbalanced diet, and low physical activity.

With respect to pre-menopausal women, lean and obese men tend to accrue more visceral than subcutaneous adipose tissue. Given its greater lipolytic potential, visceral adipose tissue strongly fuels the flux of FFA to the liver, where FFA mediate insulin resistance and NAFLD pathogenesis [20,377]. After menopause, the lack of estrogens favors the redistribution of fat towards visceral depots and relieves the inhibition of adipose lipolysis, boosting the FFA flux to the liver and increasing the risk of developing NAFLD [20]. Under obesogenic conditions, the increased insulin resistance and increased inflammation of extra-hepatic tissues showing a cross-talk with the liver, such as the adipose tissue and the skeletal muscle, can further aggravate the hepatic dysmetabolism, yielding to sex-specific and estrogen-mediated differences in obesity-induced NAFLD [20,378,379,380,381,382]. In rodent models, perinatal exposure to EDCs can increase the incidence of NAFLD later in life even by promoting the incidence of obesity in both males and females, also through transgenerational epigenetic mechanisms [359].

The two sexes display a different ability to cope with an excess of dietary lipids, with male mice more prone to develop hepatic steatosis/NAFLD when fed with HFD [17]. Estrogen deficiency contributes to dietary lipids-mediated oxidative damage and worsens liver inflammation and degeneration, accelerating NASH progression, that can be prevented by HRT [372,383]. Perinatal exposure to BPA leads to sex-specific modification of hepatic gene expression and epigenome at birth and exacerbates HFD-induced hepatic steatosis in male rodents, potentially through the epigenetic regulation of genes involved in hepatic FAO [384,385]. In HFD-fed OVX female mice, exposure to BPA exacerbates hepatic steatosis by decreasing lipid export, enhancing DNL, promoting mitochondrial and endoplasmic reticulum stress, and worsening collagen deposition and liver injury [386].

High fructose intake enhances NAFLD progression in OVX female mice by enhancing macrophage accumulation, fibrosis progression and liver damage, that can be ameliorated by estrogen supplementation [387].

In OVX female rodents fed with a high-fat high-fructose (HFHF) diet, estrogen administration combined with exercise prevents the development of insulin resistance, limits hepatic fat accumulation by increasing FAO and suppressing DNL in the liver and ameliorates the circulating lipid profile by reducing lower serum TG levels, decreasing LDL-CH and increasing HDL/CH *ratio* [388]. In addition, the combined treatment (estrogen + exercise) improves the metabolic profiles of HFHF OVX females by promoting the activation of AMPK (AMP-activated protein kinase) and the up-regulation of PGC-1α (peroxisome proliferator-activated receptor-γ coactivator-1α) and PPARδ (proliferator-activated receptor δ) in the skeletal muscle [388].

In the liver, estrogens exert their biological effects acting mainly through ERα, as highlighted by several studies performed with ERα knockout mice. Recapitulating the metabolic phenotype of estrogen-deficient animals, male and female ERαKO mice display increased body weight, visceral adiposity, glucose production, insulin resistance, and hepatic steatosis associated with a sustained hepatic inflammatory signaling [83,125,389,390,391]. In addition to the classical mechanisms, the protective effects of estrogens on liver health can be mediated also by non-nuclear mechanisms, as suggested by studies performed on transgenic mice in which the expression of ERα is limited to the cytoplasm [392,393].

However, the hepatic metabolic alterations observed in ERαKO mice are the resultant of the lack of ERα in the total body, including several organs such as adipose tissue and skeletal muscle which cross-talk with the liver. The LERKO mouse model, obtained by crossing *floxed* ERα mice with mice expressing *Cre*-recombinase under the control of albumin promoter (that it is specifically expressed in the hepatocyte cells), represents a better tool to elucidate the specific relevance of ERα in the liver [87]. Given that hepatocytes are the most abundant cell type in the liver [236] and ERα is the receptor for estrogens most expressed in the hepatocytes [39,80], the LERKO is considered, indeed, a liver-specific ERα KO mouse model.

In LERKO females, the lack of the regulatory activity of hepatic ERα leads to an altered expression of genes involved in lipid and lipoprotein metabolism during estrous cycle progression [12,81]. In LERKO females during *Proestrus* (a phase of the estrous cycle characterized by high estrogen levels), the hepatic expression of key genes involved in DNL such as *Acly* (ATP citrate lyase), *Fasn* and *Elovl6* is higher compared to control females [81], pointing to the specific relevance of hepatic ERα in mediating estrogen effects. As a consequence of the impaired regulation of hepatic lipid metabolism, LERKO females show increased deposition of lipids in the liver, which can further worsen with aging and after OVX [81]. Differently from their counterparts, LERKO females during *Proestrus* are unable to generate a class of small HDL able to efficiently promote CH efflux to the liver [12]. As a result, LERKO females display impaired hepatic CH clearance and high circulating CH levels [12,84], which may explain the increased susceptibility to atherosclerosis and CVDs in post-menopausal women [83]. The phenotype of LERKO female mice has been mostly reproduced by the liver-specific knockdown of ERα, which develop hepatic steatosis, also through the regulation of small heterodimer partner (SHP), a transcription factor relevant for the regulation of hepatic metabolic processes and hepatic inflammation [149].

Further stressing the specific relevance of hepatic ERα in the regulation of female hepatic metabolism, differently from control OVX females, estrogen treatment fails to prevent lipid deposition in the liver of LERKO females [143,394]. More recently, a specific dietary formula modified in the content of essential AA has been shown to rescue the hepatic transcriptomic profile and prevent hepatic steatosis in OVX control but not LERKO females [94]. This last study further points to the relevance of hepatic ERα in regulating the liver metabolism accordingly to hormonal and nutritional inputs (especially AA), a female-specific feature that has been selected and perfected during evolution to guarantee reproduction only under favorable conditions [75,79].

Despite its reduced expression compared to females [12], in the liver of males ERα is required for the estrogen-mediated programming of the hepatic metabolism, thus contributing to hepatic sexual dimorphism [39]. Liver-specific disruption of ERα signaling in males leads to altered expression of genes involved in GNG (e.g., *Pck1*, *G6pc*) and lipid metabolism (e.g., *Fasn*, *Acaca)* and to impaired FOXO1 phosphorylation, resulting in an insulin-resistant phenotype characterized by enhanced HGP, glucose intolerance, increased hepatic lipogenesis, lipid deposition and inflammation in the liver [80,126,138,395,396].

Given the role of liver ERα in sensing nutritional inputs and in modulating the hepatic metabolism accordingly [79], the detrimental effects associated with the lack of the hepatic ERα are particularly relevant under impaired nutritional conditions. When exposed to excess of dietary lipids, LERKO but not control females accumulate lipids in the liver, as a consequence of a reduced inhibition of genes involved in DNL and lipid import [17]. In LERKO females, the HFD-induced liver fat deposition is associated with a reduced hepatic content of AA, especially BCAA [17], in agreement with studies reporting a negative correlation between the BCAA levels in the liver and NAFLD progression [154,321]. Possibly as a consequence of its role in the achievement of hepatic sexual dimorphism, the lack of hepatic ERα signaling has opposite consequences in the liver of males and females [17,39]. When exposed to an excess of dietary lipids (HFD), LERKO males display a significantly lower hepatic content of lipids with respect to control males, due mostly to a reduced DNL (e.g., *Fasn; Elovl6; Hmgr*, 3-hydroxy-3-methylglutaryl-CoA reductase; *Pmvk,* phosphomevalonate kinase) and a limited lipid import (e.g., *Cd36; Ldlr*, LDL receptor) [17]. In LERKO males, however, the limited liver fat accumulation in response to HFD occurs at the expense of an altered plasma lipid profile with high LDL/HDL *ratio* [17], suggesting that the impairment of hepatic estrogen signaling in males may favor atherosclerosis and CVDs.

Acting mainly through ERα, estrogens inhibit the activation of JNK and NF-κB and their signaling pathways, thus reducing the expression of target genes encoding inflammatory mediators, such as TNFα, IL-1β and IL-6 [365,366,367,397], and avoiding the propagation of a chronic inflammatory status, which may account for sex differences in NAFLD progression toward more harmful conditions [71,230,251,363,364]. The lack of ERα derepresses the estrogen-mediated inhibition of several pro-inflammatory markers in the liver of both, male and female ERαKO mice, leading to the propagation of hepatic inflammation [391,396]. With respect to patients with simple steatosis, hepatic ERα expression is lower in the liver of patients with NASH, suggesting that this receptor plays a specific role in counteracting pro-inflammatory and pro-apoptotic processes that can favor NAFLD progression and liver degeneration [398]. Accordingly, even under physiological conditions, the lack of the hepatic ERα in LERKO females induces the expression of genes involved in the inflammatory process (e.g., *Tnfα; Il-1β;* interleukin-12 beta, *Il-12β; Ccr2*, C-C motif chemokine receptor 2) and collagen deposition (sequestosome1, *Sqstm1*; vimentin, *Vim*; serpine1, *Serpine*), leading to portal infiltration of mononuclear leukocytes and portal/centrilobular collagen deposition in the liver [12].

Estrogens, acting mainly through ERα, can limit liver damage by orchestrating cell proliferation, thus favoring liver regeneration, a process that further contributes to sex-differences in NAFLD progression, since male animals show higher recruitment of monocytes and a delayed recovery from acute liver injury [44,399,400,401,402]. Estrogen and ERα play an important role also in regulating the accumulation of fats in the liver by modulating CD36 during the early phase of liver regeneration, when FA, TG and CH are required for the proliferation of hepatocytes and for the formation of new cell membrane [403].

Only few studies have assessed the relevance of estrogen signaling in mediating the beneficial effects of physical activity in counteracting NAFLD [404]. Endurance training has been reported to be effective in reducing intra-hepatic lipid content and inflammation in post-menopausal women and OVX rodents [404,405,406]. Like estrogens, modest exercise improves hepatic health and mitochondrial outcomes in OVX females, by decreasing several genes of lipogenesis (e.g., *Srebp-1c*; *Chrebp*, carbohydrate-responsive element-binding protein; *Scd1*; *Acaca*) and several biomarkers of subclinical inflammation (e.g., *Nf-kb, Tnfα*, *Il-6*,) [407]. However, endurance exercise training prevents liver fat accumulation when exercise started after and not before OVX [408], suggesting that exercise must be conducted concurrently as estrogens withdrawal to be effective. ERα signaling is necessary for exercise-induced prevention of hepatic steatosis also in males, since wheel running suppresses hepatic steatosis and inflammatory gene transcripts in WT but not in ERαKO male mice when fed with an obesogenic diet [396].

Despite its lower expression with respect to ERα [94], GPER may have a role in counteracting NAFLD development and progression, also in a sex-specific fashion. In fact, GPER knockout (GPERKO) mice display increased lipid accumulation in the liver and decreased circulating HDL levels in females, but not males [105,409]. In the liver, the lack of GPER enhances immune cell infiltration, fibrosis, and the production of inflammatory factors, such as IL-6, IL-1β and TNFα [410]. The activation of GPER reduces the expression of lipogenic and pro-inflammatory genes and increases the expression of genes involved in lipid oxidation in the liver of OVX female mice [105]. GPER mediates the estrogen-dependent regulation of liver lipid uptake, by preventing the internalization of PCSK9 (proprotein convertase subtilisin/kexin type 9) and limiting LDLR (low-density lipoprotein receptor) degradation, thus lowering circulating LDL (low-density lipoprotein) cholesterol [411]. Accordingly, individuals with a hypofunctional genetic variant of GPER show increased plasma LDL cholesterol [412]. Nevertheless, the specific role of hepatic GPER signaling in the regulation of liver metabolism and inflammation remains to be clarified, given the lack of liver-specific GPER knockout models.

## 9. NAFLD and Androgen Signaling

**Figure 6:**
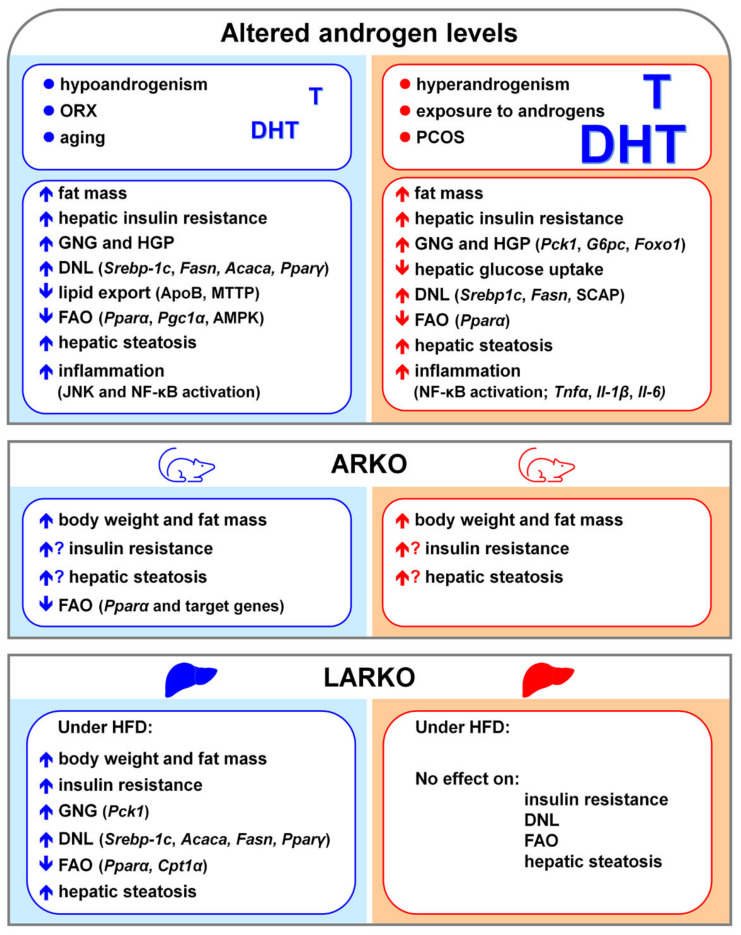
Consequences of altered androgen signaling in the liver of males (left, in blue) and females (right, in red) favoring NAFLD development and progression. Low and high androgen levels are detrimental for the hepatic health of males and females, respectively. Abbreviations: *Acaca*, acetyl-CoA carboxylase α; AMPK, AMP-activated protein kinase; ApoB; apolipoprotein B; ARKO, total AR knockout mice; *Cpt1α*, carnitine palmitotyltransferase 1; DHT, dihydrotestosterone; DNL, de novo lipogenesis; FAO, fatty acid oxidation; *Fasn*, fatty acid synthase; *Foxo1*, forkhead transcription factor 1; *G6pc*, glucose-6-phosphatase; GNG, gluconeogenesis; HGP, hepatic glucose production; *Il-1β*, interleukin 1β; *Il-6*, interleukin 6; JNK, c-Jun N-terminal kinase; LARKO, liver AR knockout mice; MTTP; microsomal triglyceride transfer protein; NF-𝜅B, nuclear factor-𝜅B; ORX, orchidectomy; *Pck1*, phosphoenolpyruvate carboxykinase 1; PCOS, poly-cystic ovary syndrome; *Pgc1α*, peroxisome proliferator-activated receptor-γ coactivator-1α; *Pparα*, peroxisome proliferator activated receptor α; *Ppar*γ, peroxisome proliferator activated receptor γ; *Scap*, sterol regulatory element-binding protein cleavage-activating protein; *Srebp1c*, sterol regulatory element binding Transcription factor 1; T, testosterone; *Tnfα*, tumor necrosis factor α.

The role of androgen signaling in counteracting or promoting NAFLD is still controversial, since some studies have indicated that androgens protect against NAFLD, while others have reported opposite findings [413,414,415,416,417]. Indeed, low androgen levels in men and high androgen levels in women facilitate NAFLD development and progression [140,413,414,416,417,418,419] (Figure 6).

In rodent males, androgen deficiency due to ORX leads to hepatic steatosis, that can be reversed by testosterone administration [190]. In a diet-induced model of NAFLD, the lack of androgens consequent to ORX results in increased hepatic lipid droplet formation, liver inflammation and hepatocyte apoptosis [420,421]. In this rodent model, liver lipid deposition is mostly due to the up-regulation of genes involved in DNL (such as *Srebp-1c* and *Fasn*) and to the inhibition of genes involved in FAO (such as *Pparα* and their target genes) and lipid export, which is mediated by Apo-B and MTTP [420,421]. Testosterone replacement attenuates HFD-induced hepatocyte apoptosis and ameliorates NAFLD in castrated male rodents [420] as well as in mice with impaired androgen signaling due to 5α-reductase deficiency [187]. However, the fact that testosterone replacement can counteract hepatic steatosis in Tfm mice, which have a not-functional AR [188], indicates that the beneficial effects of testosterone can be, at least in part, mediated by its conversion to estrogen, as further suggested by studies performed in ArKO male mice [132,148]. In males, however, more than testosterone and DHT levels *per se*, testosterone/DHT *ratio* seems to be more predictive for NAFLD development and progression [422].

Although the role of androgens in females has been less extensively investigated than in males, several reports indicate that high androgen levels trigger NAFLD development in females [140,189,191,423,424]. In normal weight female mice, DHT causes liver steatosis via transcriptional regulation of SCAP [189]. Androgen excess induces insulin resistance and NAFLD in PCOS women as well as in PCOS-like rodents [140,191,423,424]. In females, prenatal exposure to androgens alters the balance between DNL and FAO pathways and induces a pro-inflammatory status, resulting in hepatic steatosis and insulin resistance [186]. Post-natal exposure of female rodents to DHT induces hepatic steatosis, that is further exacerbated by HFD, and triggers the development of insulin resistance and of a reproductive phenotype, which reproduces the features observed in PCOS women [425]. Notably, androgen exposure, especially in the early stages of life, induces changes in differentiating tissues and drives PCOS development later in life [426], increasing the risk of developing NAFLD [427,428].

Independently of several risk factors, in men as well as in PCOS and post-menopausal women, fatty liver negatively correlates with SHBG, a glycoprotein produced by hepatocytes, that transports testosterone and other steroids in the blood plasma, reduces their metabolic clearance rate, and regulates their access to target tissues [38,140,429,430,431]. In particular, low SHBG more than testosterone levels seem to be associated with hepatic steatosis, suggesting that bioavailable, free testosterone and SHBG levels can be considered early biomarkers for NAFLD development [38,140,431,432]. Beyond its association with insulin resistance [433,434], SHBG may be a mediator, rather than a biomarker, of NAFLD. In fact, the overexpression of SHBG results to protect obesity-prone *db/db* mice from HFD-induced hepatic steatosis, even after ORX [435]. Accordingly, the over-expression of SHBG in HepG2 cells abrogates the increase in ACACA expression induced by high-glucose treatment, thus reducing lipid synthesis [432]. Furthermore, SHBG can limit NAFLD progression by mitigating ER stress in hepatocyte cells [436].

In males, androgen signaling can counteract NAFLD development and progression even by suppressing hepatic inflammation through the inhibition of JNK and NF-κB signaling [420,437]. Notably, sex dimorphic incidence of hepatic steatosis in inflammation-prone mice is abolished by ORX or by testosterone treatment of females [438]. In castrated male rodents fed with HFD, testosterone replacement attenuates hepatocyte apoptosis, by reducing the activation and expression of ER stress proteins, such as PERK (protein kinase R-like endoplasmic reticulum kinase), IRE-1α (inositol-requiring transmembrane kinase/endoribonuclease 1α), JNK, and CHOP (C/EBP homologous protein) [437].

As occurs in PCOS women, DHT treatment in PCOS-like female boosts NAFLD progression and liver degeneration by increasing the activation of NF-κB signaling, enhancing the gene expression of pro-inflammatory markers such as *Il-6*, *Il-1β*, and *Tnfα*, and by reducing the expression of pro-apoptotic markers such as LC3II (microtubule-associated protein light chain 3), thus negatively impacting on the hepatic ability to regenerate [191]. As in NAFLD patients [439], the hepatic expression of the pro-inflammatory MAPK kinase is increased in females subjected prenatally to androgen exposure [440].

Besides androgen levels, the relevance of androgen signaling in counteracting NAFLD has been highlighted by the fact that both, male and female ARKO mice develop liver steatosis and insulin resistance [441]. However, other two global ARKO mouse models do not show hepatic steatosis [442,443], possibly a consequence of different AR targeting strategies adopted and of the genetic background of the mice.

By converse, even when fed normal chow, LARKO mice develop hepatic lipid accumulation, that can worsen by HFD [177]. However, the specific contribution of hepatic AR differs between the two sexes; in fact, only LARKO male but not female mice develop hepatic steatosis and insulin resistance as a result of the over-regulation of genes involved in DNL (*Srebp-1c*, *Acaca*, and *Pparγ*) and downregulation of genes involved in FAO (e.g., *Pparα*) [173,177].

In obese men, there is a strong correlation between NAFLD and low androgen levels, especially free testosterone levels, that decrease with increasing obesity [444,445,446,447,448]. Notably, weight loss can, at least partly, restore the sex steroid profile in men [449,450], suggesting that obesity-related mechanisms, such as the increase in aromatase activity and the inhibitory effects of leptin on testosterone production [451,452], account for the lowering of androgen levels and the increased severity of NAFLD in obese men.

In obese pre-menopausal women with NAFLD, high levels of androgens are associated with increasing severity of liver pathology, but not with an increased inflammation of adipose tissue [453]. Despite the strong association between obesity and NAFLD and between obesity and PCOS, high-free androgen index is associated with increased risk of NAFLD in women with PCOS, independent of obesity [413,423]. All together, these findings suggest that increased adiposity can favor NAFLD development more in males than in females, likely a consequence of the sex-specific distribution of adipose tissue in the two sexes [20].

Impaired androgen signaling in males further increases HFD-induced hepatic lipid deposition, as demonstrated by studies performed in ORX and Tfm rodents [188,417]. In ORX rodents, DHT treatment is able to decrease HFD-induced liver lipid accumulation and CH synthesis by increasing the expression of CPT1α (carnitine palmitotyltransferase 1 α) and the phosphorylation of HMGCR via an AR-mediated pathway [417]. In Tfm mice, testosterone treatment reduces hepatic steatosis by limiting the expression of genes involved in DNL such as *Acaca* and *Fasn* [188]. Unbalanced dietary regimens enriched in lipids and sugars can be particularly detrimental for PCOS women; however, in PCOS women insulin resistance more than high androgens *per se* contributes to worse NAFLD progression, mainly due to the low inhibition of DNL and the enhanced delivery to the liver of FFA derived from the enhanced lipolysis of adipose tissue [423,424]. Nevertheless, given insulin resistance in PCOS women and PCOS-like rodents is a consequence of long-term androgen excess [191,423,424], high androgen levels in women can indirectly account for the negative effects of dietary excess of lipids and sugars on liver health, further boosting NAFLD development.

The association between low androgen levels, reduced physical activity and NAFLD development in the two sexes has been little investigated. However, the strong correlation between sarcopenia and the increased incidence of NAFLD in aging men [198,454,455], as well as between high androgen levels and sarcopenia in PCOS women [456], suggest that low physical activity may further negatively impact on the development and progression of NAFLD in both men and women with altered androgen levels. However, in these physiopathological conditions, reduced physical activity can be a confounding factor, since both, aging men and PCOS women, that are often overweight or obese, move to a less extent than young men or lean women. This evidence outlines the relevance of performing studies to discriminate the specific contribution of impaired androgen signaling and physical activity on NAFLD pathophysiology in both sexes.

## 10. Conclusions

**Figure 7:**
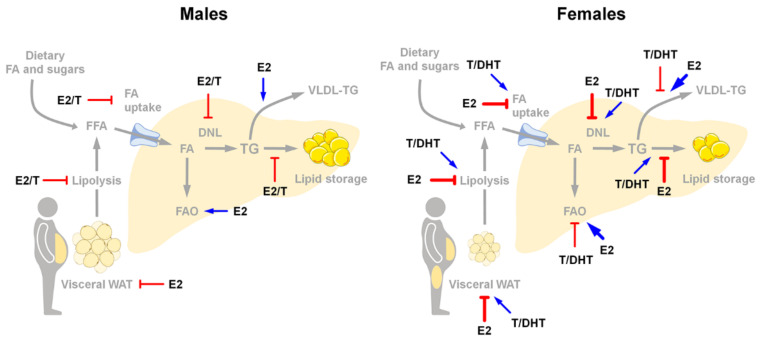
Sex-specific relevance of estrogens and androgens in the regulation of lipid metabolism in the liver of males and females accounting for differences in NAFLD susceptibility. In male liver, androgens and low levels of testosterone-derived estrogens partly limit lipid deposition by inhibiting adipose tissue lipolysis, FA uptake, and DNL, and by promoting FAO and lipid export. In female liver, estrogens prevent while high levels of androgens (especially DHT) promote lipid deposition, by differentially regulating adipose tissue lipolysis, FA uptake, DNL, FAO and lipid export. Abbreviations: DHT, dihydrotestosterone; DNL, de novo lipogenesis; E2, 17β-estradiol; FA, fatty acids; FAO, fatty acid oxidation; T, testosterone.

The data summarized in this review outline the role of sex hormones and their receptors in contributing to the sex-dependent prevalence of NAFLD. Overall, testosterone in males and estrogens in females prevent hepatic steatosis by limiting lipid accumulation through reduced DNL, by promoting FAO and hepatic lipid export, and by suppressing inflammation and oxidative stress (Figure 7). The high metabolic dynamicity conferred by estrogen signaling to female liver contributes to prevent and limit the surge and progression of hepatic metabolic and inflammatory alterations even under unbalanced dietary regimens, thus accounting for the sex-specific prevalence of NAFLD.

In males, perturbance of androgen and estrogen signaling can impair hepatic lipid metabolism and inflammation, further favoring the development NAFLD and its progression toward more harmful conditions. In females, the lack of a proper estrogen signaling in post-menopausal women as well as the exposure to high androgen levels as occurs in PCOS women trigger NAFLD development and progression, nullifying sex dimorphic incidence of this pathology. Especially in females, the impairment of sex hormones (estrogen, in particular) signaling has more negative consequence on hepatic health, likely a consequence of the alteration of the strict interplay between metabolism and reproduction gained by the female liver during evolution [75,79]. In this view, the increased lifespan of women, that is not properly balanced by increased reproductive lifespan [457,458], and the risk factors of the nowadays lifestyle (obesity, unbalanced dietary regimens, low physical activity) may impact more negatively on hepatic health of aging women than men, pointing to the need of new actions to support the research in this field. Furthermore, sex hormones signaling contributes to sex dimorphic expression of drug-metabolizing enzymes in the liver [47,459,460,461], leading often to drug-induced hepatotoxicity and to higher risk of adverse drug reaction in females compared to males [42]. In virtue of the still limited understanding of the hepatic sexual dimorphism, a better comprehension of the extent to which sex hormones and their signaling contribute to sex differences in the liver will be useful for the design of more personalized, sex-specific pharmacological therapies and interventional strategies against NAFLD and the associated co-morbidities, which incidence is greatly increasing worldwide [462,463].

## Data Availability

Not applicable.

## References

[1] Trefts E., Gannon M., Wasserman D.H. (2017). The Liver. Curr. Biol..

[2] Yang X. (2006). Tissue-Specific Expression and Regulation of Sexually Dimorphic Genes in Mice. Genome Res..

[3] Della Torre S. (2020). Non-Alcoholic Fatty Liver Disease as a Canonical Example of Metabolic Inflammatory-Based Liver Disease Showing a Sex-Specific Prevalence: Relevance of Estrogen Signaling. Front. Endocrinol..

[4] Lonardo A., Suzuki A. (2020). Sexual Dimorphism of NAFLD in Adults. Focus on Clinical Aspects and Implications for Practice and Translational Research. J. Clin. Med..

[5] Lonardo A., Nascimbeni F., Ballestri S., Fairweather D., Win S., Than T.A., Abdelmalek M.F., Suzuki A. (2019). Sex Differences in Nonalcoholic Fatty Liver Disease: State of the Art and Identification of Research Gaps. Hepatology.

[6] Maggi A., Della Torre S. (2018). Sex, Metabolism and Health. Mol. Metab..

[7] Smith R.L., Soeters M.R., Wüst R.C.I., Houtkooper R.H. (2018). Metabolic Flexibility as an Adaptation to Energy Resources and Requirements in Health and Disease. Endocr. Rev..

[8] Han H.-S., Kang G., Kim J.S., Choi B.H., Koo S.-H. (2016). Regulation of Glucose Metabolism from a Liver-Centric Perspective. Exp. Mol. Med..

[9] Bazhan N., Jakovleva T., Feofanova N., Denisova E., Dubinina A., Sitnikova N., Makarova E. (2019). Sex Differences in Liver, Adipose Tissue, and Muscle Transcriptional Response to Fasting and Refeeding in Mice. Cells.

[10] Lorbek G., Lewinska M., Rozman D. (2012). Cytochrome P450s in the Synthesis of Cholesterol and Bile Acids--from Mouse Models to Human Diseases. FEBS J..

[11] Phelps T., Snyder E., Rodriguez E., Child H., Harvey P. (2019). The Influence of Biological Sex and Sex Hormones on Bile Acid Synthesis and Cholesterol Homeostasis. Biol. Sex Differ..

[12] Della Torre S., Mitro N., Fontana R., Gomaraschi M., Favari E., Recordati C., Lolli F., Quagliarini F., Meda C., Ohlsson C. (2016). An Essential Role for Liver ERα in Coupling Hepatic Metabolism to the Reproductive Cycle. Cell Rep..

[13] Galan X., Llobera M., Ramírez I. (1994). Lipoprotein Lipase and Hepatic Lipase in Wistar and Sprague-Dawley Rat Tissues. Differences in the Effects of Gender and Fasting. Lipids.

[14] Palmisano B.T., Zhu L., Eckel R.H., Stafford J.M. (2018). Sex Differences in Lipid and Lipoprotein Metabolism. Mol. Metab..

[15] Sorrentino D., Zhou S.L., Kokkotou E., Berk P.D. (1992). Sex Differences in Hepatic Fatty Acid Uptake Reflect a Greater Affinity of the Transport System in Females. Am. J. Physiol..

[16] Ståhlberg N., Rico-Bautista E., Fisher R.M., Wu X., Cheung L., Flores-Morales A., Tybring G., Norstedt G., Tollet-Egnell P. (2004). Female-Predominant Expression of Fatty Acid Translocase/CD36 in Rat and Human Liver. Endocrinology.

[17] Meda C., Barone M., Mitro N., Lolli F., Pedretti S., Caruso D., Maggi A., Della Torre S. (2020). Hepatic ERα Accounts for Sex Differences in the Ability to Cope with an Excess of Dietary Lipids. Mol. Metab..

[18] Matthan N.R., Jalbert S.M., Barrett P.H.R., Dolnikowski G.G., Schaefer E.J., Lichtenstein A.H. (2008). Gender-Specific Differences in the Kinetics of Nonfasting TRL, IDL, and LDL Apolipoprotein B-100 in Men and Premenopausal Women. Arterioscler. Thromb. Vasc. Biol..

[19] Wang X., Magkos F., Mittendorfer B. (2011). Sex differences in Lipid and Lipoprotein Metabolism: It’s Not Just about Sex Hormones. J. Clin. Endocrinol. Metab..

[20] Goossens G.H., Jocken J.W.E., Blaak E.E. (2021). Sexual Dimorphism in Cardiometabolic Health: The Role of Adipose Tissue, Muscle and Liver. Nat. Rev. Endocrinol..

[21] Magkos F., Patterson B.W., Mohammed B.S., Klein S., Mittendorfer B. (2007). Women Produce Fewer but Triglyceride-Richer Very Low-Density Lipoproteins than Men. J. Clin. Endocrinol. Metab..

[22] Karpe F., Bickerton A.S., Hodson L., Fielding B.A., Tan G.D., Frayn K.N. (2007). Removal of Triacylglycerols from Chylomicrons and VLDL by Capillary Beds: The Basis of Lipoprotein Remnant Formation. Biochem. Soc. Trans..

[23] Magkos F., Wang X., Mittendorfer B. (2010). Metabolic Actions of Insulin in Men and Women. Nutrition.

[24] Gustavsson C., Yassin K., Wahlström E., Cheung L., Lindberg J., Brismar K., Östenson C.-G., Norstedt G., Tollet-Egnell P. (2010). Sex-Different Hepaticglycogen Content and Glucose Output in Rats. BMC Biochem..

[25] Mauvais-Jarvis F. (2018). Gender Differences in Glucose Homeostasis and Diabetes. Physiol. Behav..

[26] Tramunt B., Smati S., Grandgeorge N., Lenfant F., Arnal J.-F., Montagner A., Gourdy P. (2020). Sex Differences in Metabolic Regulation and Diabetes Susceptibility. Diabetologia.

[27] Pramfalk C., Pavlides M., Banerjee R., McNeil C.A., Neubauer S., Karpe F., Hodson L. (2015). Sex-Specific Differences in Hepatic Fat Oxidation and Synthesis May Explain the Higher Propensity for NAFLD in Men. J. Clin. Endocrinol. Metab..

[28] Luo J., Yang H., Song B.-L. (2020). Mechanisms and Regulation of Cholesterol Homeostasis. Nat. Rev. Mol. Cell Biol..

[29] Freedman D.S., Otvos J.D., Jeyarajah E.J., Shalaurova I., Cupples L.A., Parise H., D’Agostino R.B., Wilson P.W.F., Schaefer E.J. (2004). Sex and Age Differences in Lipoprotein Subclasses Measured by Nuclear Magnetic Resonance Spectroscopy: The Framingham Study. Clin. Chem..

[30] Johnson J.L., Slentz C.A., Duscha B.D., Samsa G.P., McCartney J.S., Houmard J.A., Kraus W.E. (2004). Gender and Racial Differences in Lipoprotein Subclass Distributions: The STRRIDE Study. Atherosclerosis.

[31] Velez-Carrasco W., Lichtenstein A.H., Li Z., Dolnikowski G.G., Lamon-Fava S., Welty F.K., Schaefer E.J. (2000). Apolipoprotein A-I and A-II Kinetic Parameters as Assessed by Endogenous Labeling with [(2)H(3)]Leucine in Middle-Aged and Elderly Men and Women. Arterioscler. Thromb. Vasc. Biol..

[32] Hewitt K.N., Boon W.C., Murata Y., Jones M.E.E., Simpson E.R. (2003). The Aromatase Knockout Mouse Presents with a Sexually Dimorphic Disruption to Cholesterol Homeostasis. Endocrinology.

[33] Fisher F.M., Maratos-Flier E. (2016). Understanding the Physiology of FGF21. Annu. Rev. Physiol..

[34] Gabay C., Kushner I. (1999). Acute-Phase Proteins and Other Systemic Responses to Inflammation. N. Engl. J. Med..

[35] Jensen-Cody S.O., Potthoff M.J. (2021). Hepatokines and Metabolism: Deciphering Communication from the Liver. Mol. Metab..

[36] Ohlsson C., Mohan S., Sjögren K., Tivesten Å., Isgaard J., Isaksson O., Jansson J.-O., Svensson J. (2009). The Role of Liver-Derived Insulin-Like Growth Factor-I. Endocr. Rev..

[37] Thalacker-Mercer A.E., Johnson C.A., Yarasheski K.E., Carnell N.S., Campbell W.W. (2007). Nutrient Ingestion, Protein Intake, and Sex, but Not Age, Affect the Albumin Synthesis Rate in Humans. J. Nutr..

[38] Jaruvongvanich V., Sanguankeo A., Riangwiwat T., Upala S. (2017). Testosterone, Sex Hormone-Binding Globulin and Nonalcoholic Fatty Liver Disease: A Systematic Review and Meta-Analysis. Ann. Hepatol..

[39] Della Torre S., Mitro N., Meda C., Lolli F., Pedretti S., Barcella M., Ottobrini L., Metzger D., Caruso D., Maggi A. (2018). Short-Term Fasting Reveals Amino Acid Metabolism as a Major Sex-Discriminating Factor in the Liver. Cell Metab..

[40] Heymann F., Tacke F. (2016). Immunology in the Liver–from Homeostasis to Disease. Nat. Rev. Gastroenterol. Hepatol..

[41] Kubes P., Jenne C. (2018). Immune Responses in the Liver. Annu. Rev. Immunol..

[42] Buzzetti E., Parikh P.M., Gerussi A., Tsochatzis E. (2017). Gender Differences in Liver Disease and the Drug-Dose Gender Gap. Pharmacol. Res..

[43] Klein S.L., Flanagan K.L. (2016). Sex Differences in Immune Responses. Nat. Rev. Immunol..

[44] Sutti S., Tacke F. (2018). Liver Inflammation and Regeneration in Drug-Induced Liver Injury: Sex Matters!. Clin. Sci..

[45] Fuscoe J.C., Vijay V., Hanig J.P., Han T., Ren L., Greenhaw J.J., Beger R.D., Pence L.M., Shi Q. (2020). Hepatic Transcript Profiles of Cytochrome P450 Genes Predict Sex Differences in Drug Metabolism. Drug. Metab. Dispos..

[46] Klaassen C.D., Liu L., Dunn R.T. (1998). Regulation of Sulfotransferase MRNA Expression in Male and Female Rats of Various Ages. Chem. Biol. Interact..

[47] Waxman D.J., Holloway M.G. (2009). Sex Differences in the Expression of Hepatic Drug Metabolizing Enzymes. Mol. Pharmacol..

[48] Lichanska A.M., Waters M.J. (2008). How Growth Hormone Controls Growth, Obesity and Sexual Dimorphism. Trends Genet..

[49] Waxman D.J., O’Connor C. (2006). Growth Hormone Regulation of Sex-Dependent Liver Gene Expression. Mol. Endocrinol..

[50] Liu Z., Cordoba-Chacon J., Kineman R.D., Cronstein B.N., Muzumdar R., Gong Z., Werner H., Yakar S. (2016). Growth Hormone Control of Hepatic Lipid Metabolism. Diabetes.

[51] Roy A.K., Chatterjee B. (1983). Sexual Dimorphism in the Liver. Annu. Rev. Physiol..

[52] Clodfelter K.H., Holloway M.G., Hodor P., Park S.-H., Ray W.J., Waxman D.J. (2006). Sex-Dependent Liver Gene Expression Is Extensive and Largely Dependent upon Signal Transducer and Activator of Transcription 5b (STAT5b): STAT5b-Dependent Activation of Male Genes and Repression of Female Genes Revealed by Microarray Analysis. Mol. Endocrinol..

[53] Zhang Y., Laz E.V., Waxman D.J. (2012). Dynamic, Sex-Differential STAT5 and BCL6 Binding to Sex-Biased, Growth Hormone-Regulated Genes in Adult Mouse Liver. Mol. Cell. Biol..

[54] Holloway M.G., Miles G.D., Dombkowski A.A., Waxman D.J. (2008). Liver-Specific Hepatocyte Nuclear Factor-4α Deficiency: Greater Impact on Gene Expression in Male than in Female Mouse Liver. Mol. Endocrinol..

[55] Wiwi C.A., Gupte M., Waxman D.J. (2004). Sexually Dimorphic P450 Gene Expression in Liver-Specific Hepatocyte Nuclear Factor 4α-Deficient Mice. Mol. Endocrinol..

[56] Conforto T.L., Steinhardt G.F., Waxman D.J. (2015). Cross Talk between GH-Regulated Transcription Factors HNF6 and CUX2 in Adult Mouse Liver. Mol. Endocrinol..

[57] Holloway M.G., Laz E.V., Waxman D.J. (2006). Codependence of Growth Hormone-Responsive, Sexually Dimorphic Hepatic Gene Expression on Signal Transducer and Activator of Transcription 5b and Hepatic Nuclear Factor 4α. Mol. Endocrinol..

[58] Park S.-H., Wiwi C.A., Waxman D.J. (2006). Signalling Cross-Talk between Hepatocyte Nuclear Factor 4α and Growth-Hormone-Activated STAT5b. Biochem. J..

[59] Lau-Corona D., Suvorov A., Waxman D.J. (2017). Feminization of Male Mouse Liver by Persistent Growth Hormone Stimulation: Activation of Sex-Biased Transcriptional Networks and Dynamic Changes in Chromatin States. Mol. Cell. Biol..

[60] Ling G., Sugathan A., Mazor T., Fraenkel E., Waxman D.J. (2010). Unbiased, Genome-Wide In Vivo Mapping of Transcriptional Regulatory Elements Reveals Sex Differences in Chromatin Structure Associated with Sex-Specific Liver Gene Expression. Mol. Cell. Biol..

[61] Ramirez M.C., Zubeldía-Brenner L., Wargon V., Ornstein A.M., Becu-Villalobos D. (2014). Expression and Methylation Status of Female-Predominant GH-Dependent Liver Genes Are Modified by Neonatal Androgenization in Female Mice. Mol. Cell. Endocrinol..

[62] Sugathan A., Waxman D.J. (2013). Genome-Wide Analysis of Chromatin States Reveals Distinct Mechanisms of Sex-Dependent Gene Regulation in Male and Female Mouse Liver. Mol. Cell. Biol..

[63] Conforto T.L., Waxman D.J. (2012). Sex-Specific Mouse Liver Gene Expression: Genome-Wide Analysis of Developmental Changes from Pre-Pubertal Period to Young Adulthood. Biol. Sex Differ..

[64] Lowe R., Gemma C., Rakyan V.K., Holland M.L. (2015). Sexually Dimorphic Gene Expression Emerges with Embryonic Genome Activation and Is Dynamic throughout Development. BMC Genom..

[65] Wauthier V., Sugathan A., Meyer R.D., Dombkowski A.A., Waxman D.J. (2010). Intrinsic Sex Differences in the Early Growth Hormone Responsiveness of Sex-Specific Genes in Mouse Liver. Mol. Endocrinol..

[66] Laz E.V., Holloway M.G., Chen C.-S., Waxman D.J. (2007). Characterization of Three Growth Hormone-Responsive Transcription Factors Preferentially Expressed in Adult Female Liver. Endocrinology.

[67] Bur I.M., Cohen-Solal A.M., Carmignac D., Abecassis P.-Y., Chauvet N., Martin A.O., van der Horst G.T.J., Robinson I.C.A.F., Maurel P., Mollard P. (2009). The Circadian Clock Components CRY1 and CRY2 Are Necessary to Sustain Sex Dimorphism in Mouse Liver Metabolism. J. Biol. Chem..

[68] Lomas-Soria C., Reyes-Castro L.A., Rodríguez-González G.L., Ibáñez C.A., Bautista C.J., Cox L.A., Nathanielsz P.W., Zambrano E. (2018). Maternal Obesity Has Sex-Dependent Effects on Insulin, Glucose and Lipid Metabolism and the Liver Transcriptome in Young Adult Rat Offspring: Maternal Obesity Programs Liver Transcriptome Changes in Rat Offspring. J. Physiol..

[69] Lu Y.-F., Jin T., Xu Y., Zhang D., Wu Q., Zhang Y.-K.J., Liu J. (2013). Sex Differences in the Circadian Variation of Cytochrome P450 Genes and Corresponding Nuclear Receptors in Mouse Liver. Chronobiol. Int..

[70] Nohara K., Baba T., Murai H., Kobayashi Y., Suzuki T., Tateishi Y., Matsumoto M., Nishimura N., Sano T. (2011). Global DNA Methylation in the Mouse Liver Is Affected by Methyl Deficiency and Arsenic in a Sex-Dependent Manner. Arch. Toxicol..

[71] Stanimirovic J., Obradovic M., Jovanovic A., Sudar-Milovanovic E., Zafirovic S., Pitt S.J., Stewart A.J., Isenovic E.R. (2016). A High Fat Diet Induces Sex-Specific Differences in Hepatic Lipid Metabolism and Nitrite/Nitrate in Rats. Nitric Oxide.

[72] Takasugi M., Hayakawa K., Arai D., Shiota K. (2013). Age- and Sex-Dependent DNA Hypomethylation Controlled by Growth Hormone in Mouse Liver. Mech. Ageing Dev..

[73] Wankhade U.D., Zhong Y., Kang P., Alfaro M., Chintapalli S.V., Piccolo B.D., Mercer K.E., Andres A., Thakali K.M., Shankar K. (2018). Maternal High-Fat Diet Programs Offspring Liver Steatosis in a Sexually Dimorphic Manner in Association with Changes in Gut Microbial Ecology in Mice. Sci. Rep..

[74] Weger B.D., Gobet C., Yeung J., Martin E., Jimenez S., Betrisey B., Foata F., Berger B., Balvay A., Foussier A. (2019). The Mouse Microbiome Is Required for Sex-Specific Diurnal Rhythms of Gene Expression and Metabolism. Cell Metab..

[75] Della Torre S., Maggi A. (2017). Sex Differences: A Resultant of an Evolutionary Pressure?. Cell Metab..

[76] Zheng D., Wang X., Antonson P., Gustafsson J.-Å., Li Z. (2018). Genomics of Sex Hormone Receptor Signaling in Hepatic Sexual Dimorphism. Mol. Cell. Endocrinol..

[77] van Nas A., GuhaThakurta D., Wang S.S., Yehya N., Horvath S., Zhang B., Ingram-Drake L., Chaudhuri G., Schadt E.E., Drake T.A. (2009). Elucidating the Role of Gonadal Hormones in Sexually Dimorphic Gene Coexpression Networks. Endocrinology.

[78] Zhuang Q.K.-W., Galvez J.H., Xiao Q., AlOgayil N., Hyacinthe J., Taketo T., Bourque G., Naumova A.K. (2020). Sex Chromosomes and Sex Phenotype Contribute to Biased DNA Methylation in Mouse Liver. Cells.

[79] Della Torre S., Benedusi V., Fontana R., Maggi A. (2014). Energy Metabolism and Fertility—A Balance Preserved for Female Health. Nat. Rev. Endocrinol..

[80] Qiu S., Vazquez J.T., Boulger E., Liu H., Xue P., Hussain M.A., Wolfe A. (2017). Hepatic Estrogen Receptor α Is Critical for Regulation of Gluconeogenesis and Lipid Metabolism in Males. Sci. Rep..

[81] Villa A., Della Torre S., Stell A., Cook J., Brown M., Maggi A. (2012). Tetradian Oscillation of Estrogen Receptor Is Necessary to Prevent Liver Lipid Deposition. Proc. Natl. Acad. Sci. USA.

[82] Della Torre S., Lolli F., Ciana P., Maggi A., Mauvais-Jarvis F. (2017). Sexual Dimorphism and Estrogen Action in Mouse Liver. Sex and Gender Factors Affecting Metabolic Homeostasis, Diabetes and Obesity.

[83] Palmisano B.T., Zhu L., Stafford J.M., Mauvais-Jarvis F. (2017). Role of Estrogens in the Regulation of Liver Lipid Metabolism. Sex and Gender Factors Affecting Metabolic Homeostasis, Diabetes and Obesity.

[84] Zhu L., Shi J., Luu T.N., Neuman J.C., Trefts E., Yu S., Palmisano B.T., Wasserman D.H., Linton M.F., Stafford J.M. (2018). Hepatocyte Estrogen Receptor Alpha Mediates Estrogen Action to Promote Reverse Cholesterol Transport during Western-Type Diet Feeding. Mol. Metab..

[85] Addison M.L., Rissman E.F. (2012). Sexual Dimorphism of Growth Hormone in the Hypothalamus: Regulation by Estradiol. Endocrinology.

[86] Fernández-Pérez L., Guerra B., Díaz-Chico J.C., Flores-Morales A. (2013). Estrogens Regulate the Hepatic Effects of Growth Hormone, a Hormonal Interplay with Multiple Fates. Front. Endocrinol..

[87] Della Torre S., Rando G., Meda C., Stell A., Chambon P., Krust A., Ibarra C., Magni P., Ciana P., Maggi A. (2011). Amino Acid-Dependent Activation of Liver Estrogen Receptor Alpha Integrates Metabolic and Reproductive Functions via IGF-1. Cell Metab..

[88] Fontana R., Torre S. (2016). The Deep Correlation between Energy Metabolism and Reproduction: A View on the Effects of Nutrition for Women Fertility. Nutrients.

[89] Arnold A.P., Gorski R.A. (1984). Gonadal Steroid Induction of Structural Sex differences in the Central Nervous System. Annu. Rev. Neurosci..

[90] Gorski R.A., Wagner J.W. (1965). Gonadal activity and sexual differentiation of the hypothalamus. Endocrinology.

[91] Meinhardt U.J., Ho K.K.Y. (2006). Modulation of Growth Hormone Action by Sex Steroids. Clin. Endocrinol..

[92] Biddie S.C. (2011). Chromatin Architecture and the Regulation of Nuclear Receptor Inducible Transcription: Nuclear Receptors and the Epigenetic Regulation of Chromatin. J. Neuroendocrinol..

[93] He H.H., Meyer C.A., Chen M.W., Jordan V.C., Brown M., Liu X.S. (2012). Differential DNase I Hypersensitivity Reveals Factor-Dependent Chromatin Dynamics. Genome Res..

[94] Della Torre S., Benedusi V., Pepe G., Meda C., Rizzi N., Uhlenhaut N.H., Maggi A. (2021). Dietary Essential Amino Acids Restore Liver Metabolism in Ovariectomized Mice via Hepatic Estrogen Receptor α. Nat. Commun..

[95] Ackerman G.E., Carr B.R. (2002). Estrogens. Rev. Endocr. Metab. Disord..

[96] Gruber C.J., Tschugguel W., Schneeberger C., Huber J.C. (2002). Production and Actions of Estrogens. N. Engl. J. Med..

[97] Frederiksen H., Johannsen T.H., Andersen S.E., Albrethsen J., Landersoe S.K., Petersen J.H., Andersen A.N., Vestergaard E.T., Schorring M.E., Linneberg A. (2020). Sex-Specific Estrogen Levels and Reference Intervals from Infancy to Late Adulthood Determined by LC-MS/MS. J. Clin. Endocrinol. Metab..

[98] Holinka C.F., Diczfalusy E., Coelingh Bennink H.J.T. (2008). Estetrol: A Unique Steroid in Human Pregnancy. J. Steroid. Biochem. Mol. Biol..

[99] Kobayashi H. (2020). Estrogen Synthesis in Gastric Parietal Cells and Secretion into Portal Vein. Anat. Sci. Int..

[100] Kobayashi H., Yoshida S., Sun Y.-J., Shirasawa N., Naito A. (2013). Postnatal Development of Gastric Aromatase and Portal Venous Estradiol-17β Levels in Male Rats. J. Endocrinol..

[101] Hetemäki N., Savolainen-Peltonen H., Tikkanen M.J., Wang F., Paatela H., Hämäläinen E., Turpeinen U., Haanpää M., Vihma V., Mikkola T.S. (2017). Estrogen Metabolism in Abdominal Subcutaneous and Visceral Adipose Tissue in Postmenopausal Women. J. Clin. Endocrinol. Metab..

[102] Misso M.L., Jang C., Adams J., Tran J., Murata Y., Bell R., Boon W.C., Simpson E.R., Davis S.R. (2005). Adipose Aromatase Gene Expression Is Greater in Older Women and Is Unaffected by Postmenopausal Estrogen Therapy. Menopause.

[103] Dahlman-Wright K., Cavailles V., Fuqua S.A., Jordan V.C., Katzenellenbogen J.A., Korach K.S., Maggi A., Muramatsu M., Parker M.G., Gustafsson J.-A. (2006). International Union of Pharmacology. LXIV. Estrogen Receptors. Pharmacol. Rev..

[104] Levin E.R. (2009). Plasma Membrane Estrogen Receptors. Trends Endocrinol. Metab..

[105] Sharma G., Prossnitz E.R., Mauvais-Jarvis F. (2017). G-Protein-Coupled Estrogen Receptor (GPER) and Sex-Specific Metabolic Homeostasis. Sex and Gender Factors Affecting Metabolic Homeostasis, Diabetes and Obesity.

[106] Della Torre S., Biserni A., Rando G., Monteleone G., Ciana P., Komm B., Maggi A. (2011). The Conundrum of Estrogen Receptor Oscillatory Activity in the Search for an Appropriate Hormone Replacement Therapy. Endocrinology.

[107] Jakacka M., Ito M., Weiss J., Chien P.Y., Gehm B.D., Jameson J.L. (2001). Estrogen Receptor Binding to DNA Is Not Required for Its Activity through the Nonclassical AP1 Pathway. J. Biol. Chem..

[108] Levin E.R. (2005). Integration of the Extranuclear and Nuclear Actions of Estrogen. Mol. Endocrinol..

[109] Acconcia F., Ascenzi P., Bocedi A., Spisni E., Tomasi V., Trentalance A., Visca P., Marino M. (2005). Palmitoylation-Dependent Estrogen Receptor Alpha Membrane Localization: Regulation by 17beta-Estradiol. Mol. Biol. Cell.

[110] Almazroo O.A., Miah M.K., Venkataramanan R. (2017). Drug Metabolism in the Liver. Clin. Liver Dis..

[111] Fabris G., Dumortier O., Pisani D.F., Gautier N., Van Obberghen E. (2019). Amino Acid-Induced Regulation of Hepatocyte Growth: Possible Role of Drosha. Cell Death Dis..

[112] Jenne C.N., Kubes P. (2013). Immune Surveillance by the Liver. Nat. Immunol..

[113] Nagarajan S.R., Paul-Heng M., Krycer J.R., Fazakerley D.J., Sharland A.F., Hoy A.J. (2019). Lipid and Glucose Metabolism in Hepatocyte Cell Lines and Primary Mouse Hepatocytes: A Comprehensive Resource for in Vitro Studies of Hepatic Metabolism. Am. J. Physiol. Endocrinol. Metab..

[114] Vickers A.E.M., Lucier G.W. (1996). Estrogen Receptor Levels and Occupancy in Hepatic Sinusoidal Endothelial and Kupffer Cells Are Enhanced by Initiation with Diethylnitrosamine and Promotion with 17α-Ethinylestradiol in Rats. Carcinogenesis.

[115] Kazankov K., Jørgensen S.M.D., Thomsen K.L., Møller H.J., Vilstrup H., George J., Schuppan D., Grønbæk H. (2019). The Role of Macrophages in Nonalcoholic Fatty Liver Disease and Nonalcoholic Steatohepatitis. Nat. Rev. Gastroenterol. Hepatol..

[116] Krenkel O., Tacke F. (2017). Liver Macrophages in Tissue Homeostasis and Disease. Nat. Rev. Immunol..

[117] Dixon L.J., Barnes M., Tang H., Pritchard M.T., Nagy L.E., Terjung R. (2013). Kupffer Cells in the Liver. Comprehensive Physiology.

[118] Zhou Y., Shimizu I., Lu G., Itonaga M., Okamura Y., Shono M., Honda H., Inoue S., Muramatsu M., Ito S. (2001). Hepatic Stellate Cells Contain the Functional Estrogen Receptor β but Not the Estrogen Receptor α in Male and Female Rats. Biochem. Biophys. Res. Commun..

[119] Zhang B., Zhang C.-G., Ji L.-H., Zhao G., Wu Z.-Y. (2018). Estrogen Receptor **β** Selective Agonist Ameliorates Liver Cirrhosis in Rats by Inhibiting the Activation and Proliferation of Hepatic Stellate Cells: ERβ Agonist and Liver Cirrhosis. J. Gastroenterol. Hepatol..

[120] Cortes E., Lachowski D., Rice A., Thorpe S.D., Robinson B., Yeldag G., Lee D.A., Ghemtio L., Rombouts K., del Río Hernández A.E. (2019). Tamoxifen Mechanically Deactivates Hepatic Stellate Cells via the G Protein-Coupled Estrogen Receptor. Oncogene.

[121] Banales J.M., Huebert R.C., Karlsen T., Strazzabosco M., LaRusso N.F., Gores G.J. (2019). Cholangiocyte Pathobiology. Nat. Rev. Gastroenterol. Hepatol..

[122] Raven A., Lu W.-Y., Man T.Y., Ferreira-Gonzalez S., O’Duibhir E., Dwyer B.J., Thomson J.P., Meehan R.R., Bogorad R., Koteliansky V. (2017). Cholangiocytes Act as Facultative Liver Stem Cells during Impaired Hepatocyte Regeneration. Nature.

[123] Alvaro D. (2006). Estrogens and the Pathophysiology of the Biliary Tree. World J. Gastroenterol..

[124] Alvaro D., Alpini G., Onori P., Perego L., Baroni G.S., Franchitto A., Baiocchi L., Glaser S.S., Le Sage G., Folli F. (2000). Estrogens Stimulate Proliferation of Intrahepatic Biliary Epithelium in Rats. Gastroenterology.

[125] Bryzgalova G., Gao H., Ahren B., Zierath J.R., Galuska D., Steiler T.L., Dahlman-Wright K., Nilsson S., Gustafsson J.-Å., Efendic S. (2006). Evidence That Oestrogen Receptor-α Plays an Important Role in the Regulation of Glucose Homeostasis in Mice: Insulin Sensitivity in the Liver. Diabetologia.

[126] Khristi V., Ratri A., Ghosh S., Borosha S., Dai E., Chakravarthi V.P., Rumi M.A.K., Wolfe M.W. (2019). Liver Transcriptome Data of Esr1 Knockout Male Rats Reveals Altered Expression of Genes Involved in Carbohydrate and Lipid Metabolism. Data Brief.

[127] Mauvais-Jarvis F., Clegg D.J., Hevener A.L. (2013). The Role of Estrogens in Control of Energy Balance and Glucose Homeostasis. Endocr. Rev..

[128] Manson J.E., Chlebowski R.T., Stefanick M.L., Aragaki A.K., Rossouw J.E., Prentice R.L., Anderson G., Howard B.V., Thomson C.A., LaCroix A.Z. (2013). Menopausal Hormone Therapy and Health Outcomes during the Intervention and Extended Poststopping Phases of the Women’s Health Initiative Randomized Trials. JAMA.

[129] Mauvais-Jarvis F., Manson J.E., Stevenson J.C., Fonseca V.A. (2017). Menopausal Hormone Therapy and Type 2 Diabetes Prevention: Evidence, Mechanisms, and Clinical Implications. Endocr. Rev..

[130] Van Pelt R.E., Gozansky W.S., Schwartz R.S., Kohrt W.M. (2003). Intravenous Estrogens Increase Insulin Clearance and Action in Postmenopausal Women. Am. J. Physiol. Endocrinol. Metab..

[131] D’Eon T.M., Souza S.C., Aronovitz M., Obin M.S., Fried S.K., Greenberg A.S. (2005). Estrogen Regulation of Adiposity and Fuel Partitioning: Evidence of Genomic and Non-Genomic Regulation of Lipogenic and Oxidative Pathways. J. Biol. Chem..

[132] Jones M.E.E., Thorburn A.W., Britt K.L., Hewitt K.N., Wreford N.G., Proietto J., Oz O.K., Leury B.J., Robertson K.M., Yao S. (2000). Aromatase-Deficient (ArKO) Mice Have a Phenotype of Increased Adiposity. Proc. Natl. Acad. Sci. USA.

[133] Tomaz L.M., Barbosa M.R., Farahnak Z., Lagoeiro C.G., Magosso N.S.S., Lavoie J.-M., Perez S.E.A. (2016). GLUT2 Proteins and PPARγ Transcripts Levels Are Increased in Liver of Ovariectomized Rats: Reversal Effects of Resistance Training. J. Exerc. Nutr. Biochem..

[134] Ahmed-Sowur H., Bailey C.J. (1981). Role of Ovarian Hormones in the Long-Term Control of Glucose Homeostasis Glycogen Formation and Gluconeogenesis. Ann. Nutr. Metab..

[135] Yan H., Yang W., Zhou F., Li X., Pan Q., Shen Z., Han G., Newell-Fugate A., Tian Y., Majeti R. (2019). Estrogen Improves Insulin Sensitivity and Suppresses Gluconeogenesis via the Transcription Factor Foxo1. Diabetes.

[136] Heine P.A., Taylor J.A., Iwamoto G.A., Lubahn D.B., Cooke P.S. (2000). Increased Adipose Tissue in Male and Female Estrogen Receptor-Alpha Knockout Mice. Proc. Natl. Acad. Sci. USA.

[137] Allard C., Morford J.J., Xu B., Salwen B., Xu W., Desmoulins L., Zsombok A., Kim J.K., Levin E.R., Mauvais-Jarvis F. (2019). Loss of Nuclear and Membrane Estrogen Receptor-α Differentially Impairs Insulin Secretion and Action in Male and Female Mice. Diabetes.

[138] Zhu L., Martinez M.N., Emfinger C.H., Palmisano B.T., Stafford J.M. (2014). Estrogen Signaling Prevents Diet-Induced Hepatic Insulin Resistance in Male Mice with Obesity. Am. J. Physiol. Endocrinol. Metab..

[139] Matic M., Bryzgalova G., Gao H., Antonson P., Humire P., Omoto Y., Portwood N., Pramfalk C., Efendic S., Berggren P.-O. (2013). Estrogen Signalling and the Metabolic Syndrome: Targeting the Hepatic Estrogen Receptor Alpha Action. PLoS ONE.

[140] Grossmann M., Wierman M.E., Angus P., Handelsman D.J. (2019). Reproductive Endocrinology of Nonalcoholic Fatty Liver Disease. Endocr. Rev..

[141] Hewitt K.N., Pratis K., Jones M.E.E., Simpson E.R. (2004). Estrogen Replacement Reverses the Hepatic Steatosis Phenotype in the Male Aromatase Knockout Mouse. Endocrinology.

[142] Macut D., Tziomalos K., Božić-Antić I., Bjekić-Macut J., Katsikis I., Papadakis E., Andrić Z., Panidis D. (2016). Non-Alcoholic Fatty Liver Disease Is Associated with Insulin Resistance and Lipid Accumulation Product in Women with Polycystic Ovary Syndrome. Hum. Reprod..

[143] Zhu L., Brown W.C., Cai Q., Krust A., Chambon P., McGuinness O.P., Stafford J.M. (2013). Estrogen Treatment After Ovariectomy Protects Against Fatty Liver and May Improve Pathway-Selective Insulin Resistance. Diabetes.

[144] Paquette A., Wang D., Jankowski M., Gutkowska J., Lavoie J.-M. (2008). Effects of Ovariectomy on PPARα, SREBP-1c, and SCD-1 Gene Expression in the Rat Liver. Menopause.

[145] Paquette A., Chapados N., Bergeron R., Lavoie J.-M. (2009). Fatty Acid Oxidation Is Decreased in the Liver of Ovariectomized Rats. Horm. Metab. Res..

[146] Chen Z., Wang O., Nie M., Elison K., Zhou D., Li M., Jiang Y., Xia W., Meng X., Chen S. (2015). Aromatase Deficiency in a Chinese Adult Man Caused by Novel Compound Heterozygous CYP19A1 Mutations: Effects of Estrogen Replacement Therapy on the Bone, Lipid, Liver and Glucose Metabolism. Mol. Cell. Endocrinol..

[147] Van Sinderen M.L., Steinberg G.R., Jørgensen S.B., To S.Q., Knower K.C., Clyne C.D., Honeyman J., Chow J.D., Herridge K.A., Jones M.E.E. (2014). Hepatic Glucose Intolerance Precedes Hepatic Steatosis in the Male Aromatase Knockout (ArKO) Mouse. PLoS ONE.

[148] Amano A., Kondo Y., Noda Y., Ohta M., Kawanishi N., Machida S., Mitsuhashi K., Senmaru T., Fukui M., Takaoka O. (2017). Abnormal Lipid/Lipoprotein Metabolism and High Plasma Testosterone Levels in Male but Not Female Aromatase-Knockout Mice. Arch. Biochem. Biophys..

[149] Wang X., Lu Y., Wang E., Zhang Z., Xiong X., Zhang H., Lu J., Zheng S., Yang J., Xia X. (2015). Hepatic Estrogen Receptor α Improves Hepatosteatosis through Upregulation of Small Heterodimer Partner. J. Hepatol..

[150] Zhang Z.-C., Liu Y., Xiao L.-L., Li S.-F., Jiang J.-H., Zhao Y., Qian S.-W., Tang Q.-Q., Li X. (2015). Upregulation of MiR-125b by Estrogen Protects against Non-Alcoholic Fatty Liver in Female Mice. J. Hepatol..

[151] Gerdts E., Regitz-Zagrosek V. (2019). Sex Differences in Cardiometabolic Disorders. Nat. Med..

[152] Man J.J., Beckman J.A., Jaffe I.Z. (2020). Sex as a Biological Variable in Atherosclerosis. Circ. Res..

[153] Regitz-Zagrosek V., Kararigas G. (2017). Mechanistic Pathways of Sex differences in Cardiovascular Disease. Physiol. Rev..

[154] Lake A.D., Novak P., Shipkova P., Aranibar N., Robertson D.G., Reily M.D., Lehman-McKeeman L.D., Vaillancourt R.R., Cherrington N.J. (2015). Branched Chain Amino Acid Metabolism Profiles in Progressive Human Nonalcoholic Fatty Liver Disease. Amino Acids.

[155] Obayashi M., Shimomura Y., Nakai N., Jeoung N.H., Nagasaki M., Murakami T., Sato Y., Harris R.A. (2004). Estrogen Controls Branched-Chain Amino Acid Catabolism in Female Rats. Nutr. J..

[156] Leung K.-C., Johannsson G., Leong G.M., Ho K.K.Y. (2004). Estrogen Regulation of Growth Hormone Action. Endocr. Rev..

[157] Schiffer L., Arlt W., Storbeck K.-H. (2018). Intracrine Androgen Biosynthesis, Metabolism and Action Revisited. Mol. Cell. Endocrinol..

[158] Courant F., Aksglaede L., Antignac J.-P., Monteau F., Sorensen K., Andersson A.-M., Skakkebaek N.E., Juul A., Bizec B.L. (2010). Assessment of Circulating Sex Steroid Levels in Prepubertal and Pubertal Boys and Girls by a Novel Ultrasensitive Gas Chromatography-Tandem Mass Spectrometry Method. J. Clin. Endocrinol. Metab..

[159] Travison T.G., Vesper H.W., Orwoll E., Wu F., Kaufman J.M., Wang Y., Lapauw B., Fiers T., Matsumoto A.M., Bhasin S. (2017). Harmonized Reference Ranges for Circulating Testosterone Levels in Men of Four Cohort Studies in the United States and Europe. J. Clin. Endocrinol. Metab..

[160] Davison S.L., Bell R., Donath S., Montalto J.G., Davis S.R. (2005). Androgen Levels in Adult Females: Changes with Age, Menopause, and Oophorectomy. J. Clin. Endocrinol. Metab..

[161] Kostakis E.K., Gkioni L.N., Macut D., Mastorakos G., Pasquali R., Pignatelli D. (2019). Androgens in Menopausal Women: Not Only Polycystic Ovary Syndrome. Hyperandrogenism in Women.

[162] Matsumoto T., Sakari M., Okada M., Yokoyama A., Takahashi S., Kouzmenko A., Kato S. (2013). The Androgen Receptor in Health and Disease. Annu. Rev. Physiol..

[163] Vlahopoulos S., Zimmer W.E., Jenster G., Belaguli N.S., Balk S.P., Brinkmann A.O., Lanz R.B., Zoumpourlis V.C., Schwartz R.J. (2005). Recruitment of the Androgen Receptor via Serum Response Factor Facilitates Expression of a Myogenic Gene. J. Biol. Chem..

[164] Heinlein C.A., Chang C. (2002). The Roles of Androgen Receptors and Androgen-Binding Proteins in Nongenomic Androgen Actions. Mol. Endocrinol..

[165] Treviño L.S., Gorelick D.A. (2021). The Interface of Nuclear and Membrane Steroid Signaling. Endocrinology.

[166] Thomas P. (2019). Membrane Androgen Receptors Unrelated to Nuclear Steroid Receptors. Endocrinology.

[167] Takeda H., Chodak G., Mutchnik S., Nakamoto T., Chang C. (1990). Immunohistochemical Localization of Androgen Receptors with Mono- and Polyclonal Antibodies to Androgen Receptor. J. Endocrinol..

[168] Navarro G., Allard C., Xu W., Mauvais-Jarvis F. (2015). The Role of Androgens in Metabolism, Obesity, and Diabetes in Males and Females: Androgens and Diabetes. Obesity.

[169] Azziz R., Carmina E., Chen Z., Dunaif A., Laven J.S.E., Legro R.S., Lizneva D., Natterson-Horowtiz B., Teede H.J., Yildiz B.O. (2016). Polycystic Ovary Syndrome. Nat. Rev. Dis. Primers.

[170] Grossmann M. (2011). Low Testosterone in Men with Type 2 Diabetes: Significance and Treatment. J. Clin. Endocrinol. Metab..

[171] Movérare-Skrtic S., Venken K., Andersson N., Lindberg M.K., Svensson J., Swanson C., Vanderschueren D., Oscarsson J., Gustafsson J.-Å., Ohlsson C. (2006). Dihydrotestosterone Treatment Results in Obesity and Altered Lipid Metabolism in Orchidectomized Mice. Obesity.

[172] Parthasarathy C., Renuka V.N., Balasubramanian K. (2009). Sex Steroids Enhance Insulin Receptors and Glucose Oxidation in Chang Liver Cells. Clin. Chim. Acta.

[173] Kelly D.M., Jones T.H. (2013). Testosterone: A Metabolic Hormone in Health and Disease. J. Endocrinol..

[174] Muthusamy T., Murugesan P., Balasubramanian K. (2009). Sex Steroids Deficiency Impairs Glucose Transporter 4 Expression and Its Translocation through Defective Akt Phosphorylation in Target Tissues of Adult Male Rat. Metabolism.

[175] Pal M., Gupta S. (2016). Testosterone Supplementation Improves Glucose Homeostasis despite Increasing Hepatic Insulin Resistance in Male Mouse Model of Type 2 Diabetes Mellitus. Nutr. Diabetes.

[176] Kur P., Kolasa-Wołosiuk A., Grabowska M., Kram A., Tarnowski M., Baranowska-Bosiacka I., Rzeszotek S., Piasecka M., Wiszniewska B. (2021). The Postnatal Offspring of Finasteride-Treated Male Rats Shows Hyperglycaemia, Elevated Hepatic Glycogen Storage and Altered GLUT2, IR, and AR Expression in the Liver. Int. J. Mol. Sci..

[177] Lin H.-Y., Yu I.-C., Wang R.-S., Chen Y.-T., Liu N.-C., Altuwaijri S., Hsu C.-L., Ma W.-L., Jokinen J., Sparks J.D. (2008). Increased Hepatic Steatosis and Insulin Resistance in Mice Lacking Hepatic Androgen Receptor. Hepatology.

[178] Golden S.H., Dobs A.S., Vaidya D., Szklo M., Gapstur S., Kopp P., Liu K., Ouyang P. (2007). Endogenous Sex Hormones and Glucose Tolerance Status in Postmenopausal Women. J. Clin. Endocrinol. Metab..

[179] Larsson H., Ahren B. (1996). Androgen Activity as a Risk Factor for Impaired Glucose Tolerance in Postmenopausal Women. Diabetes Care.

[180] Andrisse S., Childress S., Ma Y., Billings K., Chen Y., Xue P., Stewart A., Sonko M.L., Wolfe A., Wu S. (2017). Low-Dose Dihydrotestosterone Drives Metabolic Dysfunction via Cytosolic and Nuclear Hepatic Androgen Receptor Mechanisms. Endocrinology.

[181] Dubois V., Laurent M.R., Jardi F., Antonio L., Lemaire K., Goyvaerts L., Deldicque L., Carmeliet G., Decallonne B., Vanderschueren D. (2016). Androgen Deficiency Exacerbates High-Fat Diet-Induced Metabolic Alterations in Male Mice. Endocrinology.

[182] Mårin P., Holmäng S., Gustafsson C., Jönsson L., Kvist H., Elander A., Eldh J., Sjöström L., Holm G., Björntorp P. (1993). Androgen Treatment of Abdominally Obese Men. Obes. Res..

[183] Rochira V., Madeo B., Zirilli L., Caffagni G., Maffei L., Carani C. (2007). Oestradiol Replacement Treatment and Glucose Homeostasis in Two Men with Congenital Aromatase Deficiency: Evidence for a Role of Oestradiol and Sex Steroids Imbalance on Insulin Sensitivity in Men. Diabet. Med..

[184] Van Sinderen M., Steinberg G., Jorgensen S.B., Honeyman J., Chow J.D.Y., Simpson E.R., Jones M.E.E., Boon W.C. (2017). Sexual Dimorphism in the Glucose Homeostasis Phenotype of the Aromatase Knockout (ArKO) Mice. J. Steroid Biochem. Mol. Biol..

[185] Birzniece V. (2018). Hepatic Actions of Androgens in the Regulation of Metabolism. Curr. Opin. Endocrinol. Diabetes Obes..

[186] Abruzzese G.A., Heber M.F., Ferreira S.R., Velez L.M., Reynoso R., Pignataro O.P., Motta A.B. (2016). Prenatal Hyperandrogenism Induces Alterations That Affect Liver Lipid Metabolism. J. Endocrinol..

[187] Dowman J.K., Hopkins L.J., Reynolds G.M., Armstrong M.J., Nasiri M., Nikolaou N., van Houten E.L.A.F., Visser J.A., Morgan S.A., Lavery G.G. (2013). Loss of 5α-Reductase Type 1 Accelerates the Development of Hepatic Steatosis but Protects Against Hepatocellular Carcinoma in Male Mice. Endocrinology.

[188] Kelly D.M., Nettleship J.E., Akhtar S., Muraleedharan V., Sellers D.J., Brooke J.C., McLaren D.S., Channer K.S., Jones T.H. (2014). Testosterone Suppresses the Expression of Regulatory Enzymes of Fatty Acid Synthesis and Protects against Hepatic Steatosis in Cholesterol-Fed Androgen Deficient Mice. Life Sci..

[189] Seidu T., McWhorter P., Myer J., Alamgir R., Eregha N., Bogle D., Lofton T., Ecelbarger C., Andrisse S. (2021). DHT Causes Liver Steatosis via Transcriptional Regulation of SCAP in Normal Weight Female Mice. J. Endocrinol..

[190] Xia F., Xu X., Zhai H., Meng Y., Zhang H., Du S., Xu H., Wu H., Lu Y. (2013). Castration-Induced Testosterone Deficiency Increases Fasting Glucose Associated with Hepatic and Extra-Hepatic Insulin Resistance in Adult Male Rats. Reprod. Biol. Endocrinol..

[191] Cui P., Hu W., Ma T., Hu M., Tong X., Zhang F., Shi J., Xu X., Li X., Shao L.R. (2021). Long-Term Androgen Excess Induces Insulin Resistance and Non-Alcoholic Fatty Liver Disease in PCOS-like Rats. J. Steroid Biochem. Mol. Biol..

[192] Livingstone D.E.W., Barat P., Di Rollo E.M., Rees G.A., Weldin B.A., Rog-Zielinska E.A., MacFarlane D.P., Walker B.R., Andrew R. (2015). 5α-Reductase Type 1 Deficiency or Inhibition Predisposes to Insulin Resistance, Hepatic Steatosis, and Liver Fibrosis in Rodents. Diabetes.

[193] Kelly D.M., Akhtar S., Sellers D.J., Muraleedharan V., Channer K.S., Jones T.H. (2016). Testosterone Differentially Regulates Targets of Lipid and Glucose Metabolism in Liver, Muscle and Adipose Tissues of the Testicular Feminised Mouse. Endocrine.

[194] Krycer J.R., Brown A.J. (2011). Cross-Talk between the Androgen Receptor and the Liver X Receptor. J. Biol. Chem..

[195] Birzniece V., Meinhardt U.J., Umpleby M.A., Handelsman D.J., Ho K.K.Y. (2011). Interaction between Testosterone and Growth Hormone on Whole-Body Protein Anabolism Occurs in the Liver. J. Clin. Endocrinol. Metab..

[196] Lam T., Poljak A., McLean M., Bahl N., Ho K.K.Y., Birzniece V. (2017). Testosterone Prevents Protein Loss via the Hepatic Urea Cycle in Human. Eur. J. Endocrinol..

[197] Birzniece V., Umpleby M.A., Poljak A., Handelsman D.J., Ho K.K.Y. (2013). Oral Low-Dose Testosterone Administration Induces Whole-Body Protein Anabolism in Postmenopausal Women: A Novel Liver-Targeted Therapy. Eur. J. Endocrinol..

[198] Basualto-Alarcón C., Varela D., Duran J., Maass R., Estrada M. (2014). Sarcopenia and Androgens: A Link between Pathology and Treatment. Front. Endocrinol..

[199] Anderson L.J., Liu H., Garcia J.M., Mauvais-Jarvis F. (2017). Sex Differences in Muscle Wasting. Sex and Gender Factors Affecting Metabolic Homeostasis, Diabetes and Obesity.

[200] Anzai Á., Marcondes R.R., Gonçalves T.H., Carvalho K.C., Simões M.J., Garcia N., Soares J.M., Padmanabhan V., Baracat E.C., da Silva I.D.C.G. (2017). Impaired Branched-Chain Amino Acid Metabolism May Underlie the Nonalcoholic Fatty Liver Disease-like Pathology of Neonatal Testosterone-Treated Female Rats. Sci. Rep..

[201] Rizvi S., Weinbauer G., Arslan M., Partsch C., Nieschlag E. (2000). Testosterone Modulates Growth Hormone Secretion at the Hypothalamic but Not at the Hypophyseal Level in the Adult Male Rhesus Monkey. J. Endocrinol..

[202] Roelfsema F., Yang R.J., Takahashi P.Y., Erickson D., Bowers C.Y., Veldhuis J.D. (2018). Aromatized Estrogens Amplify Nocturnal Growth Hormone Secretion in Testosterone-Replaced Older Hypogonadal Men. J. Clin. Endocrinol. Metab..

[203] Saggese G., Cesaretti G., Franchi G., Startari L. (1996). Testosterone-Induced Increase of Insulin-like Growth Factor I Levels Depends upon Normal Levels of Growth Hormone. Eur. J. Endocrinol..

[204] Weissberger A.J., Ho K.K. (1993). Activation of the Somatotropic Axis by Testosterone in Adult Males: Evidence for the Role of Aromatization. J. Clin. Endocrinol. Metab..

[205] Veldhuis J.D., Metzger D.L., Martha P.M., Mauras N., Kerrigan J.R., Keenan B., Rogol A.D., Pincus S.M. (1997). Estrogen and Testosterone, but Not a Nonaromatizable Androgen, Direct Network Integration of the Hypothalamo-Somatotrope (Growth Hormone)-Insulin-like Growth Factor I Axis in the Human: Evidence from Pubertal Pathophysiology and Sex-Steroid Hormone Replacement. J. Clin. Endocrinol. Metab..

[206] Rochira V., Zirilli L., Maffei L., Premrou V., Aranda C., Baldi M., Ghigo E., Aimaretti G., Carani C., Lanfranco F. (2010). Tall Stature without Growth Hormone: Four Male Patients with Aromatase Deficiency. J. Clin. Endocrinol. Metab..

[207] Gibney J., Wolthers T., Johannsson G., Umpleby A.M., Ho K.K.Y. (2005). Growth Hormone and Testosterone Interact Positively to Enhance Protein and Energy Metabolism in Hypopituitary Men. Am. J. Physiol. Endocrinol. Metab..

[208] Mauras N., Rini A., Welch S., Sager B., Murphy S.P. (2003). Synergistic Effects of Testosterone and Growth Hormone on Protein Metabolism and Body Composition in Prepubertal Boys. Metabolism.

[209] Yu Y.M., Domené H.M., Sztein J., Counts D.R., Cassorla F. (1996). Developmental Changes and Differential Regulation by Testosterone and Estradiol of Growth Hormone Receptor Expression in the Rabbit. Eur. J. Endocrinol..

[210] Ramirez M.C., Luque G.M., Ornstein A.M., Becu-Villalobos D. (2010). Differential Neonatal Testosterone Imprinting of GH-Dependent Liver Proteins and Genes in Female Mice. J. Endocrinol..

[211] Ellefson W.M., Lakner A.M., Hamilton A., McKillop I.H., Bonkovsky H.L., Steuerwald N.M., Huet Y.M., Schrum L.W. (2011). Neonatal Androgenization Exacerbates Alcohol-Induced Liver Injury in Adult Rats, an Effect Abrogated by Estrogen. PLoS ONE.

[212] Buzzetti E., Pinzani M., Tsochatzis E.A. (2016). The Multiple-Hit Pathogenesis of Non-Alcoholic Fatty Liver Disease (NAFLD). Metabolism.

[213] Ferguson D., Finck B.N. (2021). Emerging Therapeutic Approaches for the Treatment of NAFLD and Type 2 Diabetes Mellitus. Nat. Rev. Endocrinol..

[214] Friedman S.L., Neuschwander-Tetri B.A., Rinella M., Sanyal A.J. (2018). Mechanisms of NAFLD Development and Therapeutic Strategies. Nat. Med..

[215] Adams L.A., Anstee Q.M., Tilg H., Targher G. (2017). Non-Alcoholic Fatty Liver Disease and its Relationship with Cardiovascular Disease and Other Extrahepatic Diseases. Gut.

[216] Estes C., Razavi H., Loomba R., Younossi Z., Sanyal A.J. (2018). Modeling the Epidemic of Nonalcoholic Fatty Liver Disease Demonstrates an Exponential Increase in Burden of Disease: Estes et Al. Hepatology.

[217] Mikolasevic I., Milic S., Turk Wensveen T., Grgic I., Jakopcic I., Stimac D., Wensveen F., Orlic L. (2016). Nonalcoholic Fatty Liver Disease—A Multisystem Disease?. World J. Gastroenterol..

[218] Younossi Z., Anstee Q.M., Marietti M., Hardy T., Henry L., Eslam M., George J., Bugianesi E. (2018). Global Burden of NAFLD and NASH: Trends, Predictions, Risk Factors and Prevention. Nat. Rev. Gastroenterol. Hepatol..

[219] Ipsen D.H., Lykkesfeldt J., Tveden-Nyborg P. (2018). Molecular Mechanisms of Hepatic Lipid Accumulation in Non-Alcoholic Fatty Liver Disease. Cell. Mol. Life Sci..

[220] Kawano Y., Cohen D.E. (2013). Mechanisms of Hepatic Triglyceride Accumulation in Non-Alcoholic Fatty Liver Disease. J. Gastroenterol..

[221] Lambert J.E., Ramos–Roman M.A., Browning J.D., Parks E.J. (2014). Increased De Novo Lipogenesis is a Distinct Characteristic of Individuals with Nonalcoholic Fatty Liver Disease. Gastroenterology.

[222] Engin A., Engin A.B., Engin A. (2017). Non-Alcoholic Fatty Liver Disease. Obesity and Lipotoxicity.

[223] Lebeaupin C., Vallée D., Hazari Y., Hetz C., Chevet E., Bailly-Maitre B. (2018). Endoplasmic Reticulum Stress Signalling and the Pathogenesis of Non-Alcoholic Fatty Liver Disease. J. Hepatol..

[224] Satapati S., Kucejova B., Duarte J.A.G., Fletcher J.A., Reynolds L., Sunny N.E., He T., Nair L.A., Livingston K.A., Livingston K. (2015). Mitochondrial Metabolism Mediates Oxidative Stress and Inflammation in Fatty Liver. J. Clin. Investig..

[225] Sunny N.E., Bril F., Cusi K. (2017). Mitochondrial Adaptation in Nonalcoholic Fatty Liver Disease: Novel Mechanisms and Treatment Strategies. Trends Endocrinol. Metab..

[226] Day C.P. (2006). From Fat to Inflammation. Gastroenterology.

[227] Hotamisligil G.S. (2006). Inflammation and Metabolic Disorders. Nature.

[228] Luedde T., Schwabe R.F. (2011). NF-ΚB in the Liver—Linking Injury, Fibrosis and Hepatocellular Carcinoma. Nat. Rev. Gastroenterol. Hepatol..

[229] Papa S., Bubici C., Zazzeroni F., Franzoso G. (2009). Mechanisms of Liver Disease: Cross-Talk between the NF-ΚB and JNK Pathways. Biol. Chem..

[230] Sharma M., Mitnala S., Vishnubhotla R.K., Mukherjee R., Reddy D.N., Rao P.N. (2015). The Riddle of Nonalcoholic Fatty Liver Disease: Progression From Nonalcoholic Fatty Liver to Nonalcoholic Steatohepatitis. J. Clin. Exp. Hepatol..

[231] Chen Z., Yu R., Xiong Y., Du F., Zhu S. (2017). A Vicious Circle between Insulin Resistance and Inflammation in Nonalcoholic Fatty Liver Disease. Lipids Health Dis..

[232] Kanda T., Matsuoka S., Yamazaki M., Shibata T., Nirei K., Takahashi H., Kaneko T., Fujisawa M., Higuchi T., Nakamura H. (2018). Apoptosis and Non-Alcoholic Fatty Liver Diseases. World J. Gastroenterol..

[233] Schuster S., Cabrera D., Arrese M., Feldstein A.E. (2018). Triggering and Resolution of Inflammation in NASH. Nat. Rev. Gastroenterol. Hepatol..

[234] Solinas G., Becattini B. (2017). JNK at the Crossroad of Obesity, Insulin Resistance, and Cell Stress Response. Mol. Metab..

[235] Yan H., Gao Y., Zhang Y. (2017). Inhibition of JNK Suppresses Autophagy and Attenuates Insulin Resistance in a Rat Model of Nonalcoholic Fatty Liver Disease. Mol. Med. Rep..

[236] Stanger B.Z. (2015). Cellular Homeostasis and Repair in the Mammalian Liver. Annu. Rev. Physiol..

[237] Fausto N., Campbell J.S., Riehle K.J. (2006). Liver Regeneration. Hepatology.

[238] Kumar S., Duan Q., Wu R., Harris E.N., Su Q. (2021). Pathophysiological Communication between Hepatocytes and Non-Parenchymal Cells in Liver Injury from NAFLD to Liver Fibrosis. Adv. Drug Deliv. Rev..

[239] Koyama Y., Brenner D.A. (2017). Liver Inflammation and Fibrosis. J. Clin. Investig..

[240] Tsuchida T., Friedman S.L. (2017). Mechanisms of Hepatic Stellate Cell Activation. Nat. Rev. Gastroenterol. Hepatol..

[241] Zhang C.-Y., Yuan W.-G., He P., Lei J.-H., Wang C.-X. (2016). Liver Fibrosis and Hepatic Stellate Cells: Etiology, Pathological Hallmarks and Therapeutic Targets. World J. Gastroenterol..

[242] Anstee Q.M., Reeves H.L., Kotsiliti E., Govaere O., Heikenwalder M. (2019). From NASH to HCC: Current Concepts and Future Challenges. Nat. Rev. Gastroenterol. Hepatol..

[243] Wree A., Broderick L., Canbay A., Hoffman H.M., Feldstein A.E. (2013). From NAFLD to NASH to Cirrhosis—New Insights into Disease Mechanisms. Nat. Rev. Gastroenterol. Hepatol..

[244] Mantovani A., Zaza G., Byrne C.D., Lonardo A., Zoppini G., Bonora E., Targher G. (2018). Nonalcoholic Fatty Liver Disease Increases Risk of Incident Chronic Kidney Disease: A Systematic Review and Meta-Analysis. Metabolism.

[245] Motamed N., Rabiee B., Poustchi H., Dehestani B., Hemasi G.R., Khonsari M.R., Maadi M., Saeedian F.S., Zamani F. (2017). Non-Alcoholic Fatty Liver Disease (NAFLD) and 10-Year Risk of Cardiovascular Diseases. Clin. Res. Hepatol. Gastroenterol..

[246] Principi M., Iannone A., Losurdo G., Mangia M., Shahini E., Albano F., Rizzi S.F., La Fortezza R.F., Lovero R., Contaldo A. (2018). Nonalcoholic Fatty Liver Disease in Inflammatory Bowel Disease: Prevalence and Risk Factors. Inflamm. Bowel Dis..

[247] Stahl E.P., Dhindsa D.S., Lee S.K., Sandesara P.B., Chalasani N.P., Sperling L.S. (2019). Nonalcoholic Fatty Liver Disease and the Heart. J. Am. Coll. Cardiol..

[248] Stols-Gonçalves D., Hovingh G.K., Nieuwdorp M., Holleboom A.G. (2019). NAFLD and Atherosclerosis: Two Sides of the Same Dysmetabolic Coin?. Trends Endocrinol. Metab..

[249] Targher G., Byrne C.D., Tilg H. (2020). NAFLD and Increased Risk of Cardiovascular Disease: Clinical Associations, Pathophysiological Mechanisms and Pharmacological Implications. Gut.

[250] Upala S., Jaruvongvanich V., Wijarnpreecha K., Sanguankeo A. (2017). Nonalcoholic Fatty Liver Disease and Osteoporosis: A Systematic Review and Meta-Analysis. J. Bone Miner. Metab..

[251] Ballestri S., Nascimbeni F., Baldelli E., Marrazzo A., Romagnoli D., Lonardo A. (2017). NAFLD as a Sexual Dimorphic Disease: Role of Gender and Reproductive Status in the Development and Progression of Nonalcoholic Fatty Liver Disease and Inherent Cardiovascular Risk. Adv. Ther..

[252] Gong Z., Tas E., Yakar S., Muzumdar R. (2017). Hepatic Lipid Metabolism and Non-Alcoholic Fatty Liver Disease in Aging. Mol. Cell. Endocrinol..

[253] Papatheodoridi A.-M., Chrysavgis L., Koutsilieris M., Chatzigeorgiou A. (2020). The Role of Senescence in the Development of Nonalcoholic Fatty Liver Disease and Progression to Nonalcoholic Steatohepatitis. Hepatology.

[254] Stahl E.C., Haschak M.J., Popovic B., Brown B.N. (2018). Macrophages in the Aging Liver and Age-Related Liver Disease. Front. Immunol..

[255] Gutierrez-Grobe Y., Ponciano-Rodríguez G., Ramos M.H., Uribe M., Méndez-Sánchez N. (2010). Prevalence of Non Alcoholic Fatty Liver Disease in Premenopausal, Posmenopausal and Polycystic Ovary Syndrome Women. The Role of Estrogens. Ann. Hepatol..

[256] Matsuo K., Gualtieri M.R., Cahoon S.S., Jung C.E., Paulson R.J., Shoupe D., Muderspach L.I., Wakatsuki A., Wright J.D., Roman L.D. (2016). Surgical Menopause and Increased Risk of Nonalcoholic Fatty Liver Disease in Endometrial Cancer. Menopause.

[257] Clark J.M., Brancati F.L., Diehl A.M. (2002). Nonalcoholic Fatty Liver Disease. Gastroenterology.

[258] Anderson E.L., Howe L.D., Jones H.E., Higgins J.P.T., Lawlor D.A., Fraser A. (2015). The Prevalence of Non-Alcoholic Fatty Liver Disease in Children and Adolescents: A Systematic Review and Meta-Analysis. PLoS ONE.

[259] Lu J., Zhang J., Du R., Wang T., Xu M., Xu Y., Wang W., Bi Y., Li D., Chen Y. (2017). Age at Menarche Is Associated with the Prevalence of Non-Alcoholic Fatty Liver Disease Later in Life. J. Diabetes.

[260] Mueller N.T., Pereira M.A., Demerath E.W., Dreyfus J.G., MacLehose R.F., Carr J.J., Terry J.G., Jacobs D.R. (2015). Earlier Menarche Is Associated with Fatty Liver and Abdominal Ectopic Fat in Midlife, Independent of Young Adult BMI: The CARDIA Study: Menarcheal Timing, Abdominal Fat, and NAFLD. Obesity.

[261] Suzuki A., Abdelmalek M.F., Schwimmer J.B., Lavine J.E., Scheimann A.O., Unalp–Arida A., Yates K.P., Sanyal A.J., Guy C.D., Diehl A.M. (2012). Association Between Puberty and Features of Nonalcoholic Fatty Liver Disease. Clin. Gastroenterol. Hepatol..

[262] Yi K.H., Hwang J.S., Lim S.W., Lee J.A., Kim D.H., Lim J.S. (2017). Early Menarche is Associated with Non-Alcoholic Fatty Liver Disease in Adulthood. Pediatr. Int..

[263] Eslam M., Valenti L., Romeo S. (2018). Genetics and Epigenetics of NAFLD and NASH: Clinical Impact. J. Hepatol..

[264] Kovalic A.J., Banerjee P., Tran Q.T., Singal A.K., Satapathy S.K. (2018). Genetic and Epigenetic Culprits in the Pathogenesis of Nonalcoholic Fatty Liver Disease. J. Clin. Exp. Hepatol..

[265] Fazel Y., Koenig A.B., Sayiner M., Goodman Z.D., Younossi Z.M. (2016). Epidemiology and Natural History of Non-Alcoholic Fatty Liver Disease. Metabolism.

[266] Donnelly K.L., Smith C.I., Schwarzenberg S.J., Jessurun J., Boldt M.D., Parks E.J. (2005). Sources of Fatty Acids Stored in Liver and Secreted via Lipoproteins in Patients with Nonalcoholic Fatty Liver Disease. J. Clin. Investig..

[267] Fabbrini E., Magkos F., Mohammed B.S., Pietka T., Abumrad N.A., Patterson B.W., Okunade A., Klein S. (2009). Intrahepatic Fat, Not Visceral Fat, Is Linked with Metabolic Complications of Obesity. Proc. Natl. Acad. Sci. USA.

[268] Wilson C.G., Tran J.L., Erion D.M., Vera N.B., Febbraio M., Weiss E.J. (2016). Hepatocyte-Specific Disruption of CD36 Attenuates Fatty Liver and Improves Insulin Sensitivity in HFD-Fed Mice. Endocrinology.

[269] Petersen K.F., Dufour S., Savage D.B., Bilz S., Solomon G., Yonemitsu S., Cline G.W., Befroy D., Zemany L., Kahn B.B. (2007). The Role of Skeletal Muscle Insulin Resistance in the Pathogenesis of the Metabolic Syndrome. Proc. Natl. Acad. Sci. USA.

[270] Farrell G.C., Haczeyni F., Chitturi S., Yu J. (2018). Pathogenesis of NASH: How Metabolic Complications of Overnutrition Favour Lipotoxicity and Pro-Inflammatory Fatty Liver Disease. Obesity, Fatty Liver and Liver Cancer.

[271] Mota M., Banini B.A., Cazanave S.C., Sanyal A.J. (2016). Molecular Mechanisms of Lipotoxicity and Glucotoxicity in Nonalcoholic Fatty Liver Disease. Metabolism.

[272] Henao-Mejia J., Elinav E., Jin C., Hao L., Mehal W.Z., Strowig T., Thaiss C.A., Kau A.L., Eisenbarth S.C., Jurczak M.J. (2012). Inflammasome-Mediated Dysbiosis Regulates Progression of NAFLD and Obesity. Nature.

[273] Jornayvaz F.R., Samuel V.T., Shulman G.I. (2010). The Role of Muscle Insulin Resistance in the Pathogenesis of Atherogenic Dyslipidemia and Nonalcoholic Fatty Liver Disease Associated with the Metabolic Syndrome. Annu. Rev. Nutr..

[274] Nachit M., Leclercq I.A. (2019). Emerging Awareness on the Importance of Skeletal Muscle in Liver Diseases: Time to Dig Deeper into Mechanisms!. Clin. Sci..

[275] Polyzos S.A., Kountouras J., Mantzoros C.S. (2015). Leptin in Nonalcoholic Fatty Liver Disease: A Narrative Review. Metabolism.

[276] Safari Z., Gérard P. (2019). The Links between the Gut Microbiome and Non-Alcoholic Fatty Liver Disease (NAFLD). Cell. Mol. Life Sci..

[277] Kautzky-Willer A., Handisurya A. (2009). Metabolic Diseases and Associated Complications: Sex and Gender Matter!. Eur. J. Clin. Investig..

[278] Ye Q., Zou B., Yeo Y.H., Li J., Huang D.Q., Wu Y., Yang H., Liu C., Kam L.Y., Tan X.X.E. (2020). Global Prevalence, Incidence, and Outcomes of Non-Obese or Lean Non-Alcoholic Fatty Liver Disease: A Systematic Review and Meta-Analysis. Lancet Gastroenterol. Hepatol..

[279] Petersen M.C., Vatner D.F., Shulman G.I. (2017). Regulation of Hepatic Glucose Metabolism in Health and Disease. Nat. Rev. Endocrinol..

[280] Samuel V.T., Shulman G.I. (2016). The Pathogenesis of Insulin Resistance: Integrating Signaling Pathways and Substrate Flux. J. Clin. Investig..

[281] Watt M.J., Miotto P.M., De Nardo W., Montgomery M.K. (2019). The Liver as an Endocrine Organ—Linking NAFLD and Insulin Resistance. Endocr. Rev..

[282] Santoleri D., Titchenell P.M. (2019). Resolving the Paradox of Hepatic Insulin Resistance. Cell. Mol. Gastroenterol. Hepatol..

[283] Sunny N.E., Parks E.J., Browning J.D., Burgess S.C. (2011). Excessive Hepatic Mitochondrial TCA Cycle and Gluconeogenesis in Humans with Nonalcoholic Fatty Liver Disease. Cell Metab..

[284] De Bandt J.-P., Jegatheesan P., Tennoune-El-Hafaia N. (2018). Muscle Loss in Chronic Liver Diseases: The Example of Nonalcoholic Liver Disease. Nutrients.

[285] Seko Y., Sumida Y., Tanaka S., Mori K., Taketani H., Ishiba H., Hara T., Okajima A., Umemura A., Nishikawa T. (2018). Insulin Resistance Increases the Risk of Incident Type 2 Diabetes Mellitus in Patients with Non-Alcoholic Fatty Liver Disease: HOMA-IR Predicts Development T2DM in NAFLD. Hepatol. Res..

[286] Tilg H., Moschen A.R., Roden M. (2017). NAFLD and Diabetes Mellitus. Nat. Rev. Gastroenterol. Hepatol..

[287] Couchepin C., Le K.-A., Bortolotti M., da Encarnacao J.A., Oboni J.-B., Tran C., Schneiter P., Tappy L. (2008). Markedly Blunted Metabolic Effects of Fructose in Healthy Young Female Subjects Compared With Male Subjects. Diabetes Care.

[288] Ter Horst K.W., Gilijamse P.W., de Weijer B.A., Kilicarslan M., Ackermans M.T., Nederveen A.J., Nieuwdorp M., Romijn J.A., Serlie M.J. (2015). Sexual Dimorphism in Hepatic, Adipose Tissue, and Peripheral Tissue Insulin Sensitivity in Obese Humans. Front. Endocrinol..

[289] Perdomo C.M., Frühbeck G., Escalada J. (2019). Impact of Nutritional Changes on Nonalcoholic Fatty Liver Disease. Nutrients.

[290] Eslamparast T., Tandon P., Raman M. (2017). Dietary Composition Independent of Weight Loss in the Management of Non-Alcoholic Fatty Liver Disease. Nutrients.

[291] Ter Horst K.W., Serlie M.J. (2017). Fructose Consumption, Lipogenesis, and Non-Alcoholic Fatty Liver Disease. Nutrients.

[292] Lim J.S., Mietus-Snyder M., Valente A., Schwarz J.-M., Lustig R.H. (2010). The Role of Fructose in the Pathogenesis of NAFLD and the Metabolic Syndrome. Nat. Rev. Gastroenterol. Hepatol..

[293] Yki-Järvinen H., Luukkonen P.K., Hodson L., Moore J.B. (2021). Dietary Carbohydrates and Fats in Nonalcoholic Fatty Liver Disease. Nat. Rev. Gastroenterol. Hepatol..

[294] Shiferaw B., Verrill L., Booth H., Zansky S.M., Norton D.M., Crim S., Henao O.L. (2012). Sex-Based Differences in Food Consumption: Foodborne Diseases Active Surveillance Network (FoodNet) Population Survey, 2006–2007. Clin. Infect. Dis..

[295] Wardle J., Haase A.M., Steptoe A., Nillapun M., Jonwutiwes K., Bellisle F. (2004). Gender Differences in Food Choice: The Contribution of Health Beliefs and Dieting. Ann. Behav. Med..

[296] Wolongevicz D.M., Zhu L., Pencina M.J., Kimokoti R.W., Newby P.K., D’Agostino R.B., Millen B.E. (2010). Diet Quality and Obesity in Women: The Framingham Nutrition Studies. Br. J. Nutr..

[297] Vitale M., Masulli M., Cocozza S., Anichini R., Babini A.C., Boemi M., Bonora E., Buzzetti R., Carpinteri R., Caselli C. (2016). Sex Differences in Food Choices, Adherence to Dietary Recommendations and Plasma Lipid Profile in Type 2 Diabetes—The TOSCA.IT Study. Nutr. Metab. Cardiovasc. Dis..

[298] Zámbó V., Simon-Szabó L., Szelényi P., Kereszturi É., Bánhegyi G., Csala M. (2013). Lipotoxicity in the Liver. World J. Hepatol..

[299] Jump D.B. (2011). Fatty Acid Regulation of Hepatic Lipid Metabolism. Curr. Opin. Clin. Nutr. Metab. Care.

[300] Leamy A.K., Egnatchik R.A., Young J.D. (2013). Molecular Mechanisms and the Role of Saturated Fatty Acids in the Progression of Non-Alcoholic Fatty Liver Disease. Prog. Lipid Res..

[301] Dhibi M., Brahmi F., Mnari A., Houas Z., Chargui I., Bchir L., Gazzah N., Alsaif M.A., Hammami M. (2011). The Intake of High Fat Diet with Different Trans Fatty Acid Levels Differentially Induces Oxidative Stress and Non Alcoholic Fatty Liver Disease (NAFLD) in Rats. Nutr. Metab..

[302] da Silva-Santi L., Antunes M., Caparroz-Assef S., Carbonera F., Masi L., Curi R., Visentainer J., Bazotte R. (2016). Liver Fatty Acid Composition and Inflammation in Mice Fed with High-Carbohydrate Diet or High-Fat Diet. Nutrients.

[303] Yki-Järvinen H. (2015). Nutritional Modulation of Non-Alcoholic Fatty Liver Disease and Insulin Resistance. Nutrients.

[304] Ranković S., Popović T., Martačić J.D., Petrović S., Tomić M., Ignjatović Đ., Tovilović-Kovačević G., Glibetić M. (2017). Liver Phospholipids Fatty Acids Composition in Response to Different Types of Diets in Rats of Both Sexes. Lipids Health Dis..

[305] Basaranoglu M. (2013). Fructose as a Key Player in the Development of Fatty Liver Disease. World J. Gastroenterol..

[306] Jegatheesan P., De Bandt J. (2017). Fructose and NAFLD: The Multifaceted Aspects of Fructose Metabolism. Nutrients.

[307] Jensen T., Abdelmalek M.F., Sullivan S., Nadeau K.J., Green M., Roncal C., Nakagawa T., Kuwabara M., Sato Y., Kang D.-H. (2018). Fructose and Sugar: A Major Mediator of Non-Alcoholic Fatty Liver Disease. J. Hepatol..

[308] Alwahsh S.M., Gebhardt R. (2017). Dietary Fructose as a Risk Factor for Non-Alcoholic Fatty Liver Disease (NAFLD). Arch. Toxicol..

[309] Softic S., Stanhope K.L., Boucher J., Divanovic S., Lanaspa M.A., Johnson R.J., Kahn C.R. (2020). Fructose and Hepatic Insulin Resistance. Crit. Rev. Clin. Lab. Sci..

[310] Tappy L., Lê K.-A. (2010). Metabolic Effects of Fructose and the Worldwide Increase in Obesity. Physiol. Rev..

[311] Wei Y., Wang D., Pagliassotti M.J. (2005). Fructose Selectively Modulates C-Jun N-Terminal Kinase Activity and Insulin Signaling in Rat Primary Hepatocytes. J. Nutr..

[312] Lambertz J., Weiskirchen S., Landert S., Weiskirchen R. (2017). Fructose: A Dietary Sugar in Crosstalk with Microbiota Contributing to the Development and Progression of Non-Alcoholic Liver Disease. Front. Immunol..

[313] Comitato R., Saba A., Turrini A., Arganini C., Virgili F. (2015). Sex Hormones and Macronutrient Metabolism. Crit. Rev. Food Sci. Nutr..

[314] Hyer M.M., Dyer S.K., Kloster A., Adrees A., Taetzsch T., Feaster J., Valdez G., Neigh G.N. (2019). Sex Modifies the Consequences of Extended Fructose Consumption on Liver Health, Motor Function, and Physiological Damage in Rats. Am. J. Physiol. Regul. Integr. Comp. Physiol..

[315] Low W., Cornfield T., Charlton C., Tomlinson J., Hodson L. (2018). Sex Differences in Hepatic De Novo Lipogenesis with Acute Fructose Feeding. Nutrients.

[316] Morrell A., Tripet B.P., Eilers B.J., Tegman M., Thompson D., Copié V., Burkhead J.L. (2020). Copper Modulates Sex-Specific Fructose Hepatoxicity in Nonalcoholic Fatty Liver Disease (NALFD) Wistar Rat Models. J. Nutr. Biochem..

[317] De Chiara F., Ureta Checcllo C., Ramón Azcón J. (2019). High Protein Diet and Metabolic Plasticity in Non-Alcoholic Fatty Liver Disease: Myths and Truths. Nutrients.

[318] Harris R.A., Joshi M., Jeoung N.H., Obayashi M. (2005). Overview of the Molecular and Biochemical Basis of Branched-Chain Amino Acid Catabolism. J. Nutr..

[319] Honda T., Ishigami M., Luo F., Lingyun M., Ishizu Y., Kuzuya T., Hayashi K., Nakano I., Ishikawa T., Feng G.-G. (2017). Branched-Chain Amino Acids Alleviate Hepatic Steatosis and Liver Injury in Choline-Deficient High-Fat Diet Induced NASH Mice. Metabolism.

[320] Takegoshi K., Honda M., Okada H., Takabatake R., Matsuzawa-Nagata N., Campbell J.S., Nishikawa M., Shimakami T., Shirasaki T., Sakai Y. (2017). Branched-Chain Amino Acids Prevent Hepatic Fibrosis and Development of Hepatocellular Carcinoma in a Non-Alcoholic Steatohepatitis Mouse Model. Oncotarget.

[321] Gaggini M., Carli F., Rosso C., Buzzigoli E., Marietti M., Della Latta V., Ciociaro D., Abate M.L., Gambino R., Cassader M. (2018). Altered Amino Acid Concentrations in NAFLD: Impact of Obesity and Insulin Resistance: Gaggini et Al. Hepatology.

[322] Grzych G., Vonghia L., Bout M.-A., Weyler J., Verrijken A., Dirinck E., Joncquel M., Van Gaal L., Paumelle R., Francque S. (2020). Plasma BCAA Changes in Patients with NAFLD Are Sex Dependent. J. Clin. Endocrinol. Metab..

[323] Kim D., Vazquez-Montesino L.M., Li A.A., Cholankeril G., Ahmed A. (2020). Inadequate Physical Activity and Sedentary Behavior Are Independent Predictors of Nonalcoholic Fatty Liver Disease. Hepatology.

[324] Rodriguez B., Torres D.M., Harrison S.A. (2012). Physical Activity: An Essential Component of Lifestyle Modification in NAFLD. Nat. Rev. Gastroenterol. Hepatol..

[325] Romero-Gómez M., Zelber-Sagi S., Trenell M. (2017). Treatment of NAFLD with Diet, Physical Activity and Exercise. J. Hepatol..

[326] Pereira R.M., da Cruz Rodrigues K.C., Anaruma C.P., Sant’Ana M.R., de Campos T.D.P., Gaspar R.S., Canciglieri R.D.S., de Melo D.G., Mekary R.A., da Silva A.S.R. (2019). Short-Term Strength Training Reduces Gluconeogenesis and NAFLD in Obese Mice. J. Endocrinol..

[327] Sung K.-C., Ryu S., Lee J.-Y., Kim J.-Y., Wild S.H., Byrne C.D. (2016). Effect of Exercise on the Development of New Fatty Liver and the Resolution of Existing Fatty Liver. J. Hepatol..

[328] Van der Windt D.J., Sud V., Zhang H., Tsung A., Huang H. (2018). The Effects of Physical Exercise on Fatty Liver Disease. Gene Expr..

[329] Caudwell P., Gibbons C., Finlayson G., Näslund E., Blundell J. (2014). Exercise and Weight Loss: No Sex Differences in Body Weight Response to Exercise. Exerc. Sport Sci. Rev..

[330] Christensen P., Meinert Larsen T., Westerterp-Plantenga M., Macdonald I., Martinez J.A., Handjiev S., Poppitt S., Hansen S., Ritz C., Astrup A. (2018). Men and Women Respond Differently to Rapid Weight Loss: Metabolic Outcomes of a Multi-Centre Intervention Study after a Low-Energy Diet in 2500 Overweight, Individuals with Pre-Diabetes (PREVIEW). Diabetes Obes. Metab..

[331] Doucet E., St-Pierre S., Alméras N., Imbeault P., Mauriège P., Pascot A., Després J.-P., Tremblay A. (2002). Reduction of Visceral Adipose Tissue during Weight Loss. Eur. J. Clin. Nutr..

[332] Hagobian T.A., Evero N. (2013). Exercise and Weight Loss: What Is the Evidence of Sex Differences?. Curr. Obes. Rep..

[333] Lovejoy J.C., Sainsbury A. (2009). Stock Conference 2008 Working Group Sex Differences in Obesity and the Regulation of Energy Homeostasis. Obes. Rev..

[334] White U.A., Tchoukalova Y.D. (2014). Sex Dimorphism and Depot Differences in Adipose Tissue Function. Biochim. Biophys. Acta.

[335] Vilar-Gomez E., Martinez-Perez Y., Calzadilla-Bertot L., Torres-Gonzalez A., Gra-Oramas B., Gonzalez-Fabian L., Friedman S.L., Diago M., Romero-Gomez M. (2015). Weight Loss Through Lifestyle Modification Significantly Reduces Features of Nonalcoholic Steatohepatitis. Gastroenterology.

[336] McGee S.L., Hargreaves M. (2020). Exercise Adaptations: Molecular Mechanisms and Potential Targets for Therapeutic Benefit. Nat. Rev. Endocrinol..

[337] Melo L., Tilmant K., Hagar A., Klaunig J.E. (2021). Effect of Endurance Exercise Training on Liver Gene Expression in Male and Female Mice. Appl. Physiol. Nutr. Metab..

[338] Severinsen M.C.K., Pedersen B.K. (2020). Muscle–Organ Crosstalk: The Emerging Roles of Myokines. Endocr. Rev..

[339] Camera D.M., Anderson M.J., Hawley J.A., Carey A.L. (2010). Short-Term Endurance Training Does Not Alter the Oxidative Capacity of Human Subcutaneous Adipose Tissue. Eur. J. Appl. Physiol..

[340] Stanford K.I., Goodyear L.J. (2016). Exercise Regulation of Adipose Tissue. Adipocyte.

[341] Tsiloulis T., Carey A.L., Bayliss J., Canny B., Meex R.C.R., Watt M.J. (2018). No Evidence of White Adipocyte Browning after Endurance Exercise Training in Obese Men. Int. J. Obes..

[342] Beaudry K.M., Devries M.C. (2019). Sex-Based Differences in Hepatic and Skeletal Muscle Triglyceride Storage and Metabolism 1. Appl. Physiol. Nutr. Metab..

[343] Burra P., Bizzaro D., Gonta A., Shalaby S., Gambato M., Morelli M.C., Trapani S., Floreani A., Marra F., Brunetto M.R. (2021). Clinical Impact of Sexual Dimorphism in Non-alcoholic Fatty Liver Disease (NAFLD) and Non-alcoholic Steatohepatitis (NASH). Liver Int..

[344] Henderson G.C. (2014). Sexual Dimorphism in the Effects of Exercise on Metabolism of Lipids to Support Resting Metabolism. Front. Endocrinol..

[345] Haizlip K.M., Harrison B.C., Leinwand L.A. (2015). Sex-Based Differences in Skeletal Muscle Kinetics and Fiber-Type Composition. Physiology.

[346] Lundsgaard A.-M., Kiens B. (2014). Gender Differences in Skeletal Muscle Substrate Metabolism-Molecular Mechanisms and Insulin Sensitivity. Front. Endocrinol..

[347] McCoin C.S., Von Schulze A., Allen J., Fuller K.N.Z., Xia Q., Koestler D.C., Houchen C.J., Maurer A., Dorn G.W., Shankar K. (2019). Sex Modulates Hepatic Mitochondrial Adaptations to High-Fat Diet and Physical Activity. Am. J. Physiol. Endocrinol. Metab..

[348] Brady C.W. (2015). Liver Disease in Menopause. World J. Gastroenterol..

[349] DiStefano J.K. (2020). NAFLD and NASH in Postmenopausal Women: Implications for Diagnosis and Treatment. Endocrinology.

[350] Barsalani R., Chapados N., Lavoie J.-M. (2010). Hepatic VLDL-TG Production and MTP Gene Expression Are Decreased in Ovariectomized Rats: Effects of Exercise Training. Horm. Metab. Res..

[351] Palmisano B.T., Le T.D., Zhu L., Lee Y.K., Stafford J.M. (2016). Cholesteryl Ester Transfer Protein Alters Liver and Plasma Triglyceride Metabolism through Two Liver Networks in Female Mice. J. Lipid Res..

[352] Kim J.H., Meyers M.S., Khuder S.S., Abdallah S.L., Muturi H.T., Russo L., Tate C.R., Hevener A.L., Najjar S.M., Leloup C. (2014). Tissue-Selective Estrogen Complexes with Bazedoxifene Prevent Metabolic Dysfunction in Female Mice. Mol. Metab..

[353] Smith G.I., Reeds D.N., Okunade A.L., Patterson B.W., Mittendorfer B. (2014). Systemic Delivery of Estradiol, but Not Testosterone or Progesterone, Alters Very Low Density Lipoprotein-Triglyceride Kinetics in Postmenopausal Women. J. Clin. Endocrinol. Metab..

[354] Zegura B., Guzic-Salobir B., Sebestjen M., Keber I. (2006). The Effect of Various Menopausal Hormone Therapies on Markers of Inflammation, Coagulation, Fibrinolysis, Lipids, and Lipoproteins in Healthy Postmenopausal Women. Menopause.

[355] Moro L., Arbini A.A., Hsieh J.-T., Ford J., Simpson E.R., Hajibeigi A., Öz O.K. (2010). Aromatase Deficiency Inhibits the Permeability Transition in Mouse Liver Mitochondria. Endocrinology.

[356] Nemoto Y., Toda K., Ono M., Fujikawa-Adachi K., Saibara T., Onishi S., Enzan H., Okada T., Shizuta Y. (2000). Altered Expression of Fatty Acid–Metabolizing Enzymes in Aromatase-Deficient Mice. J. Clin. Investig..

[357] Yoshikawa T. (2002). Aromatase-Deficient (ArKO) Mice Are Retrieved from Severe Hepatic Steatosis by Peroxisome Proliferator Administration. Hepatol. Res..

[358] Djouadi F., Weinheimer C.J., Saffitz J.E., Pitchford C., Bastin J., Gonzalez F.J., Kelly D.P. (1998). A Gender-Related Defect in Lipid Metabolism and Glucose Homeostasis in Peroxisome Proliferator- Activated Receptor Alpha- Deficient Mice. J. Clin. Investig..

[359] Cano R., Pérez J.L., Dávila L.A., Ortega Á., Gómez Y., Valero-Cedeño N.J., Parra H., Manzano A., Véliz Castro T.I., Albornoz M.P.D. (2021). Role of Endocrine-Disrupting Chemicals in the Pathogenesis of Non-Alcoholic Fatty Liver Disease: A Comprehensive Review. Int. J. Mol. Sci..

[360] Foulds C.E., Treviño L.S., York B., Walker C.L. (2017). Endocrine-Disrupting Chemicals and Fatty Liver Disease. Nat. Rev. Endocrinol..

[361] Stratakis N., Conti D.V., Jin R., Margetaki K., Valvi D., Siskos A.P., Maitre L., Garcia E., Varo N., Zhao Y. (2020). Prenatal Exposure to Perfluoroalkyl Substances Associated With Increased Susceptibility to Liver Injury in Children. Hepatology.

[362] Treviño L.S., Katz T.A. (2018). Endocrine Disruptors and Developmental Origins of Nonalcoholic Fatty Liver Disease. Endocrinology.

[363] Ministrini S., Montecucco F., Sahebkar A., Carbone F. (2020). Macrophages in the Pathophysiology of NAFLD: The Role of Sex Differences. Eur. J. Clin. Investig..

[364] Naugler W.E., Sakurai T., Kim S., Maeda S., Kim K., Elsharkawy A.M., Karin M. (2007). Gender Disparity in Liver Cancer Due to Sex Differences in MyD88-Dependent IL-6 Production. Science.

[365] Evans M.J., Eckert A., Lai K., Adelman S.J., Harnish D.C. (2001). Reciprocal Antagonism between Estrogen Receptor and NF-ΚB Activity In Vivo. Circ. Res..

[366] Galmés-Pascual B.M., Martínez-Cignoni M.R., Morán-Costoya A., Bauza-Thorbrügge M., Sbert-Roig M., Valle A., Proenza A.M., Lladó I., Gianotti M. (2020). 17β-Estradiol Ameliorates Lipotoxicity-Induced Hepatic Mitochondrial Oxidative Stress and Insulin Resistance. Free Radic. Biol. Med..

[367] Kalaitzidis D., Gilmore T.D. (2005). Transcription Factor Cross-Talk: The Estrogen Receptor and NF-ΚB. Trends Endocrinol. Metab..

[368] Win S., Min R.W.M., Chen C.Q., Zhang J., Chen Y., Li M., Suzuki A., Abdelmalek M.F., Wang Y., Aghajan M. (2019). Expression of Mitochondrial Membrane–Linked SAB Determines Severity of Sex-Dependent Acute Liver Injury. J. Clin. Investig..

[369] Benedusi V., Martini E., Kallikourdis M., Villa A., Meda C., Maggi A. (2015). Ovariectomy Shortens the Life Span of Female Mice. Oncotarget.

[370] Kireev R.A., Tresguerres A.C.F., Garcia C., Borras C., Ariznavarreta C., Vara E., Vina J., Tresguerres J.A.F. (2010). Hormonal Regulation of Pro-Inflammatory and Lipid Peroxidation Processes in Liver of Old Ovariectomized Female Rats. Biogerontology.

[371] Rogers N.H., Perfield J.W., Strissel K.J., Obin M.S., Greenberg A.S. (2009). Reduced Energy Expenditure and Increased Inflammation Are Early Events in the Development of Ovariectomy-Induced Obesity. Endocrinology.

[372] Kamada Y., Kiso S., Yoshida Y., Chatani N., Kizu T., Hamano M., Tsubakio M., Takemura T., Ezaki H., Hayashi N. (2011). Estrogen Deficiency Worsens Steatohepatitis in Mice Fed High-Fat and High-Cholesterol Diet. Am. J. Physiol. Gastrointest. Liver Physiol..

[373] Klair J.S., Yang J.D., Abdelmalek M.F., Guy C.D., Gill R.M., Yates K., Unalp-Arida A., Lavine J.E., Clark J.M., Diehl A.M. (2016). A Longer Duration of Estrogen Deficiency Increases Fibrosis Risk among Postmenopausal Women with Nonalcoholic Fatty Liver Disease: Steatohepatitis/Metabolic Liver Disease. Hepatology.

[374] Li C., Xu M.M., Wang K., Adler A.J., Vella A.T., Zhou B. (2018). Macrophage Polarization and Meta-Inflammation. Transl. Res..

[375] Sica A., Invernizzi P., Mantovani A. (2014). Macrophage Plasticity and Polarization in Liver Homeostasis and Pathology: Sica et Al. Hepatology.

[376] Villa A., Rizzi N., Vegeto E., Ciana P., Maggi A. (2015). Estrogen Accelerates the Resolution of Inflammation in Macrophagic Cells. Sci. Rep..

[377] Verrijken A., Francque S., Gaal L.V. (2010). The Role of Visceral Adipose Tissue in the Pathogenesis of Non-Alcoholic Fatty Liver Disease. Eur. Endocrinol..

[378] Meyer M.R., Clegg D.J., Prossnitz E.R., Barton M. (2011). Obesity, Insulin Resistance and Diabetes: Sex Differences and Role of Oestrogen Receptors: Obesity, Insulin Resistance and Diabetes: Role of Oestrogen. Acta Physiol..

[379] Azzu V., Vacca M., Virtue S., Allison M., Vidal-Puig A. (2020). Adipose Tissue-Liver Cross Talk in the Control of Whole-Body Metabolism: Implications in Nonalcoholic Fatty Liver Disease. Gastroenterology.

[380] Gancheva S., Jelenik T., Álvarez-Hernández E., Roden M. (2018). Interorgan Metabolic Crosstalk in Human Insulin Resistance. Physiol. Rev..

[381] Kurt Z., Barrere-Cain R., LaGuardia J., Mehrabian M., Pan C., Hui S.T., Norheim F., Zhou Z., Hasin Y., Lusis A.J. (2018). Tissue-Specific Pathways and Networks Underlying Sexual Dimorphism in Non-Alcoholic Fatty Liver Disease. Biol. Sex Differ..

[382] Varghese M., Griffin C., McKernan K., Eter L., Lanzetta N., Agarwal D., Abrishami S., Singer K. (2019). Sex Differences in Inflammatory Responses to Adipose Tissue Lipolysis in Diet-Induced Obesity. Endocrinology.

[383] Besse-Patin A., Léveillé M., Oropeza D., Nguyen B.N., Prat A., Estall J.L. (2017). Estrogen Signals Through Peroxisome Proliferator-Activated Receptor-γ Coactivator 1α to Reduce Oxidative Damage Associated With Diet-Induced Fatty Liver Disease. Gastroenterology.

[384] Strakovsky R.S., Wang H., Engeseth N.J., Flaws J.A., Helferich W.G., Pan Y.-X., Lezmi S. (2015). Developmental Bisphenol A (BPA) Exposure Leads to Sex-Specific Modification of Hepatic Gene Expression and Epigenome at Birth That May Exacerbate High-Fat Diet-Induced Hepatic Steatosis. Toxicol. Appl. Pharmacol..

[385] Wei J., Sun X., Chen Y., Li Y., Song L., Zhou Z., Xu B., Lin Y., Xu S. (2014). Perinatal Exposure to Bisphenol A Exacerbates Nonalcoholic Steatohepatitis-like Phenotype in Male Rat Offspring Fed on a High-Fat Diet. J. Endocrinol..

[386] Figueiredo L.S., Oliveira K.M., Freitas I.N., Silva J.A., Silva J.N., Favero-Santos B.C., Bonfleur M.L., Carneiro E.M., Ribeiro R.A. (2020). Bisphenol-A Exposure Worsens Hepatic Steatosis in Ovariectomized Mice Fed on a High-Fat Diet: Role of Endoplasmic Reticulum Stress and Fibrogenic Pathways. Life Sci..

[387] Ohashi T., Kato M., Yamasaki A., Kuwano A., Suzuki H., Kohjima M., Ogawa Y. (2018). Effects of High Fructose Intake on Liver Injury Progression in High Fat Diet Induced Fatty Liver Disease in Ovariectomized Female Mice. Food Chem. Toxicol..

[388] Buniam J., Chukijrungroat N., Khamphaya T., Weerachayaphorn J., Saengsirisuwan V. (2019). Estrogen and Voluntary Exercise Attenuate Cardiometabolic Syndrome and Hepatic Steatosis in Ovariectomized Rats Fed a High-Fat High-Fructose Diet. Am. J. Physiol. Endocrinol. Metab..

[389] Barros R.P.A., Gustafsson J.-Å. (2011). Estrogen Receptors and the Metabolic Network. Cell Metab..

[390] Evans M.J., Lai K., Shaw L.J., Harnish D.C., Chadwick C.C. (2002). Estrogen Receptor α Inhibits IL-1β Induction of Gene Expression in the Mouse Liver. Endocrinology.

[391] Ribas V., Nguyen M.T.A., Henstridge D.C., Nguyen A.-K., Beaven S.W., Watt M.J., Hevener A.L. (2010). Impaired Oxidative Metabolism and Inflammation Are Associated with Insulin Resistance in ERα-Deficient Mice. Am. J. Physiol. Endocrinol. Metab..

[392] Chambliss K.L., Barrera J., Umetani M., Umetani J., Kim S.H., Madak-Erdogan Z., Huang L., Katzenellenbogen B.S., Katzenellenbogen J.A., Mineo C. (2016). Nonnuclear Estrogen Receptor Activation Improves Hepatic Steatosis in Female Mice. Endocrinology.

[393] Pedram A., Razandi M., O’Mahony F., Harvey H., Harvey B.J., Levin E.R. (2013). Estrogen Reduces Lipid Content in the Liver Exclusively from Membrane Receptor Signaling. Sci. Signal..

[394] Guillaume M., Riant E., Fabre A., Raymond-Letron I., Buscato M., Davezac M., Tramunt B., Montagner A., Smati S., Zahreddine R. (2019). Selective Liver Estrogen Receptor α Modulation Prevents Steatosis, Diabetes, and Obesity Through the Anorectic Growth Differentiation Factor 15 Hepatokine in Mice. Hepatol. Commun..

[395] Khristi V., Ratri A., Ghosh S., Pathak D., Borosha S., Dai E., Roy R., Chakravarthi V.P., Wolfe M.W., Karim Rumi M.A. (2019). Disruption of ESR1 Alters the Expression of Genes Regulating Hepatic Lipid and Carbohydrate Metabolism in Male Rats. Mol. Cell. Endocrinol..

[396] Winn N.C., Jurrissen T.J., Grunewald Z.I., Cunningham R.P., Woodford M.L., Kanaley J.A., Lubahn D.B., Manrique-Acevedo C., Rector R.S., Vieira-Potter V.J. (2019). Estrogen Receptor-α Signaling Maintains Immunometabolic Function in Males and Is Obligatory for Exercise-Induced Amelioration of Nonalcoholic Fatty Liver. Am. J. Physiol. Endocrinol. Metab..

[397] Sun B., Karin M. (2008). NF-ΚB Signaling, Liver Disease and Hepatoprotective Agents. Oncogene.

[398] Erkan G., Yilmaz G., Konca Degertekin C., Akyol G., Ozenirler S. (2013). Presence and Extent of Estrogen Receptor-Alpha Expression in Patients with Simple Steatosis and NASH. Pathol. Res. Pract..

[399] Bizzaro D., Crescenzi M., Di Liddo R., Arcidiacono D., Cappon A., Bertalot T., Amodio V., Tasso A., Stefani A., Bertazzo V. (2018). Sex-Dependent Differences in Inflammatory Responses during Liver Regeneration in a Murine Model of Acute Liver Injury. Clin. Sci..

[400] Kao T.-L., Kuan Y.-P., Cheng W.-C., Chang W.-C., Jeng L.-B., Yeh S., Ma W.-L. (2018). Estrogen Receptors Orchestrate Cell Growth and Differentiation to Facilitate Liver Regeneration. Theranostics.

[401] Tsugawa Y., Natori M., Handa H., Imai T. (2019). Estradiol Accelerates Liver Regeneration through Estrogen Receptor α. Clin. Exp. Gastroenterol..

[402] Uebi T., Umeda M., Imai T. (2015). Estrogen Induces Estrogen Receptor Alpha Expression and Hepatocyte Proliferation in the Livers of Male Mice. Genes Cells.

[403] Srisowanna N., Choijookhuu N., Yano K., Batmunkh B., Ikenoue M., Nhat Huynh Mai N., Yamaguchi Y., Hishikawa Y. (2019). The Effect of Estrogen on Hepatic Fat Accumulation during Early Phase of Liver Regeneration after Partial Hepatectomy in Rats. Acta Histochem. Cytochem..

[404] Lavoie J.-M., Pighon A. (2012). NAFLD, Estrogens, and Physical Exercise: The Animal Model. J. Nutr. Metab..

[405] Venetsanaki V., Polyzos S.A. (2019). Menopause and Non-Alcoholic Fatty Liver Disease: A Review Focusing on Therapeutic Perspectives. Curr. Vasc. Pharmacol..

[406] Witayavanitkul N., Werawatganon D., Chayanupatkul M., Klaikeaw N., Sanguanrungsirikul S., Siriviriyakul P. (2020). Genistein and Exercise Modulated Lipid Peroxidation and Improved Steatohepatitis in Ovariectomized Rats. BMC Complement. Med. Ther..

[407] Fuller K.N.Z., McCoin C.S., Von Schulze A.T., Houchen C.J., Choi M.A., Thyfault J.P. (2021). Estradiol Treatment or Modest Exercise Improves Hepatic Health and Mitochondrial Outcomes in Female Mice Following Ovariectomy. Am. J. Physiol. Endocrinol. Metab..

[408] Pighon A., Barsalani R., Yasari S., Prud’homme D., Lavoie J.-M. (2010). Does Exercise Training Prior to Ovariectomy Protect against Liver and Adipocyte Fat Accumulation in Rats?. Climacteric.

[409] Meoli L., Isensee J., Zazzu V., Nabzdyk C.S., Soewarto D., Witt H., Foryst-Ludwig A., Kintscher U., Noppinger P.R. (2014). Sex- and Age-Dependent Effects of Gpr30 Genetic Deletion on the Metabolic and Cardiovascular Profiles of Diet-Induced Obese Mice. Gene.

[410] Wei T., Chen W., Wen L., Zhang J., Zhang Q., Yang J., Liu H., Chen B.W., Zhou Y., Feng X. (2016). G Protein-Coupled Estrogen Receptor Deficiency Accelerates Liver Tumorigenesis by Enhancing Inflammation and Fibrosis. Cancer Lett..

[411] Fu W., Gao X.-P., Zhang S., Dai Y.-P., Zou W.-J., Yue L.-M. (2020). 17β-Estradiol Inhibits PCSK9-Mediated LDLR Degradation Through GPER/PLC Activation in HepG2 Cells. Front. Endocrinol..

[412] Hussain Y., Ding Q., Connelly P.W., Brunt J.H., Ban M.R., McIntyre A.D., Huff M.W., Gros R., Hegele R.A., Feldman R.D. (2015). G-Protein Estrogen Receptor as a Regulator of Low-Density Lipoprotein Cholesterol Metabolism: Cellular and Population Genetic Studies. Arterioscler. Thromb. Vasc. Biol..

[413] Cai J., Wu C.H., Zhang Y., Wang Y.Y., Xu W.D., Lin T.C., Li S.X., Wang L.H., Zheng J., Sun Y. (2017). High-Free Androgen Index Is Associated with Increased Risk of Non-Alcoholic Fatty Liver Disease in Women with Polycystic Ovary Syndrome, Independent of Obesity and Insulin Resistance. Int. J. Obes..

[414] Kim S., Kwon H., Park J.-H., Cho B., Kim D., Oh S.-W., Lee C.M., Choi H.-C. (2012). A Low Level of Serum Total Testosterone Is Independently Associated with Nonalcoholic Fatty Liver Disease. BMC Gastroenterol..

[415] Magyar Z., Bekesi G., Racz K., Feher J., Schaff Z., Lengyel G., Blazovics A., Illyes G., Szombath D., Hrabak A. (2011). Increased Total Scavenger Capacity and Decreased Liver Fat Content in Rats Fed Dehydroepiandrosterone and Its Sulphate on a High-Fat Diet. Gerontology.

[416] Sarkar M., Wellons M., Cedars M.I., VanWagner L., Gunderson E.P., Ajmera V., Torchen L., Siscovick D., Carr J.J., Terry J.G. (2017). Testosterone Levels in Pre-Menopausal Women Are Associated With Nonalcoholic Fatty Liver Disease in Midlife. Am. J. Gastroenterol..

[417] Zhang H., Liu Y., Wang L., Li Z., Zhang H., Wu J., Rahman N., Guo Y., Li D., Li N. (2013). Differential Effects of Estrogen/Androgen on the Prevention of Nonalcoholic Fatty Liver Disease in the Male Rat. J. Lipid Res..

[418] Cerda C., Pérez-Ayuso R.M., Riquelme A., Soza A., Villaseca P., Sir-Petermann T., Espinoza M., Pizarro M., Solis N., Miquel J.F. (2007). Nonalcoholic Fatty Liver Disease in Women with Polycystic Ovary Syndrome. J. Hepatol..

[419] Paschou S.A., Polyzos S.A., Anagnostis P., Goulis D.G., Kanaka-Gantenbein C., Lambrinoudaki I., Georgopoulos N.A., Vryonidou A. (2020). Nonalcoholic Fatty Liver Disease in Women with Polycystic Ovary Syndrome. Endocrine.

[420] Nikolaenko L., Jia Y., Wang C., Diaz-Arjonilla M., Yee J.K., French S.W., Liu P.Y., Laurel S., Chong C., Lee K. (2014). Testosterone Replacement Ameliorates Nonalcoholic Fatty Liver Disease in Castrated Male Rats. Endocrinology.

[421] Senmaru T., Fukui M., Okada H., Mineoka Y., Yamazaki M., Tsujikawa M., Hasegawa G., Kitawaki J., Obayashi H., Nakamura N. (2013). Testosterone Deficiency Induces Markedly Decreased Serum Triglycerides, Increased Small Dense LDL, and Hepatic Steatosis Mediated by Dysregulation of Lipid Assembly and Secretion in Mice Fed a High-Fat Diet. Metabolism.

[422] Münzker J., Hofer D., Trummer C., Ulbing M., Harger A., Pieber T., Owen L., Keevil B., Brabant G., Lerchbaum E. (2015). Testosterone to Dihydrotestosterone Ratio as a New Biomarker for an Adverse Metabolic Phenotype in the Polycystic Ovary Syndrome. J. Clin. Endocrinol. Metab..

[423] Condorelli R.A., Calogero A.E., Di Mauro M., Mongioi’ L.M., Cannarella R., Rosta G., La Vignera S. (2018). Androgen Excess and Metabolic Disorders in Women with PCOS: Beyond the Body Mass Index. J. Endocrinol. Investig..

[424] Petta S., Ciresi A., Bianco J., Geraci V., Boemi R., Galvano L., Magliozzo F., Merlino G., Craxì A., Giordano C. (2017). Insulin Resistance and Hyperandrogenism Drive Steatosis and Fibrosis Risk in Young Females with PCOS. PLoS ONE.

[425] Caldwell A.S.L., Middleton L.J., Jimenez M., Desai R., McMahon A.C., Allan C.M., Handelsman D.J., Walters K.A. (2014). Characterization of Reproductive, Metabolic, and Endocrine Features of Polycystic Ovary Syndrome in Female Hyperandrogenic Mouse Models. Endocrinology.

[426] Gur E.B., Karadeniz M., Turan G.A. (2015). Fetal Programming of Polycystic Ovary Syndrome. World J. Diabetes.

[427] Rocha A.L.L., Faria L.C., Guimarães T.C.M., Moreira G.V., Cândido A.L., Couto C.A., Reis F.M. (2017). Non-Alcoholic Fatty Liver Disease in Women with Polycystic Ovary Syndrome: Systematic Review and Meta-Analysis. J. Endocrinol. Investig..

[428] Salva-Pastor N., Chávez-Tapia N.C., Uribe M., Nuño-Lámbarri N. (2019). Understanding the Association of Polycystic Ovary Syndrome and Non-Alcoholic Fatty Liver Disease. J. Steroid Biochem. Mol. Biol..

[429] Polyzos S.A., Kountouras J., Tsatsoulis A., Zafeiriadou E., Katsiki E., Patsiaoura K., Zavos C., Anastasiadou V.V., Slavakis A. (2013). Sex Steroids and Sex Hormone-Binding Globulin in Postmenopausal Women with Nonalcoholic Fatty Liver Disease. Hormones.

[430] Qu X., Donnelly R. (2020). Sex Hormone-Binding Globulin (SHBG) as an Early Biomarker and Therapeutic Target in Polycystic Ovary Syndrome. Int. J. Mol. Sci..

[431] Wang X., Xie J., Pang J., Zhang H., Chen X., Lin J., Li Q., Chen Q., Ma J., Xu X. (2020). Serum SHBG Is Associated With the Development and Regression of Nonalcoholic Fatty Liver Disease: A Prospective Study. J. Clin. Endocrinol. Metab..

[432] Sáez-López C., Salcedo-Allende M.T., Hernandez C., Simó-Servat O., Simó R., Selva D.M. (2019). Sex Hormone-Binding Globulin Expression Correlates With Acetyl-Coenzyme A Carboxylase and Triglyceride Content in Human Liver. J. Clin. Endocrinol. Metab..

[433] Le T.N., Nestler J.E., Strauss J.F., Wickham E.P. (2012). Sex Hormone-Binding Globulin and Type 2 Diabetes Mellitus. Trends Endocrinol. Metab..

[434] Winters S.J., Gogineni J., Karegar M., Scoggins C., Wunderlich C.A., Baumgartner R., Ghooray D.T. (2014). Sex Hormone-Binding Globulin Gene Expression and Insulin Resistance. J. Clin. Endocrinol. Metab..

[435] Saez-Lopez C., Barbosa-Desongles A., Hernandez C., Dyer R.A., Innis S.M., Simó R., Selva D.M. (2017). Sex Hormone-Binding Globulin Reduction in Metabolic Disorders May Play a Role in NAFLD Development. Endocrinology.

[436] Kornicka-Garbowska K., Bourebaba L., Röcken M., Marycz K. (2021). Sex Hormone Binding Globulin (SHBG) Mitigates ER Stress in Hepatocytes In Vitro and Ex Vivo. Cells.

[437] Jia Y., Yee J.K., Wang C., Nikolaenko L., Diaz-Arjonilla M., Cohen J.N., French S.W., Liu P.Y., Lue Y., Lee W.-N.P. (2018). Testosterone Protects High-Fat/Low-Carbohydrate Diet-Induced Nonalcoholic Fatty Liver Disease in Castrated Male Rats Mainly via Modulating Endoplasmic Reticulum Stress. Am. J. Physiol. Endocrinol. Metab..

[438] Schwinge D., Carambia A., Quaas A., Krech T., Wegscheid C., Tiegs G., Prinz I., Lohse A.W., Herkel J., Schramm C. (2015). Testosterone Suppresses Hepatic Inflammation by the Downregulation of IL-17, CXCL-9, and CXCL-10 in a Mouse Model of Experimental Acute Cholangitis. J. Immunol..

[439] Greco D., Kotronen A., Westerbacka J., Puig O., Arkkila P., Kiviluoto T., Laitinen S., Kolak M., Fisher R.M., Hamsten A. (2008). Gene Expression in Human NAFLD. Am. J. Physiol. Gastrointest. Liver Physiol..

[440] Hogg K., Wood C., McNeilly A.S., Duncan W.C. (2011). The in Utero Programming Effect of Increased Maternal Androgens and a Direct Fetal Intervention on Liver and Metabolic Function in Adult Sheep. PLoS ONE.

[441] Lin H.-Y., Xu Q., Yeh S., Wang R.-S., Sparks J.D., Chang C. (2005). Insulin and Leptin Resistance with Hyperleptinemia in Mice Lacking Androgen Receptor. Diabetes.

[442] Fan W., Yanase T., Nomura M., Okabe T., Goto K., Sato T., Kawano H., Kato S., Nawata H. (2005). Androgen Receptor Null Male Mice Develop Late-Onset Obesity Caused by Decreased Energy Expenditure and Lipolytic Activity but Show Normal Insulin Sensitivity with High Adiponectin Secretion. Diabetes.

[443] Rana K., Fam B.C., Clarke M.V., Pang T.P.S., Zajac J.D., MacLean H.E. (2011). Increased Adiposity in DNA Binding-Dependent Androgen Receptor Knockout Male Mice Associated with Decreased Voluntary Activity and Not Insulin Resistance. Am. J. Physiol. Endocrinol. Metab..

[444] Derby C.A., Zilber S., Brambilla D., Morales K.H., McKinlay J.B. (2006). Body Mass Index, Waist Circumference and Waist to Hip Ratio and Change in Sex Steroid Hormones: The Massachusetts Male Ageing Study. Clin. Endocrinol..

[445] Giagulli V.A., Kaufman J.M., Vermeulen A. (1994). Pathogenesis of the Decreased Androgen Levels in Obese Men. J. Clin. Endocrinol. Metab..

[446] Mohr B.A., Bhasin S., Link C.L., O’Donnell A.B., McKinlay J.B. (2006). The Effect of Changes in Adiposity on Testosterone Levels in Older Men: Longitudinal Results from the Massachusetts Male Aging Study. Eur. J. Endocrinol..

[447] Nielsen T.L., Hagen C., Wraae K., Brixen K., Petersen P.H., Haug E., Larsen R., Andersen M. (2007). Visceral and Subcutaneous Adipose Tissue Assessed by Magnetic Resonance Imaging in Relation to Circulating Androgens, Sex Hormone-Binding Globulin, and Luteinizing Hormone in Young Men. J. Clin. Endocrinol. Metab..

[448] Völzke H., Aumann N., Krebs A., Nauck M., Steveling A., Lerch M.M., Rosskopf D., Wallaschofski H. (2010). Hepatic Steatosis Is Associated with Low Serum Testosterone and High Serum DHEAS Levels in Men. Int. J. Androl..

[449] Pellitero S., Olaizola I., Alastrue A., Martínez E., Granada M.L., Balibrea J.M., Moreno P., Serra A., Navarro-Díaz M., Romero R. (2012). Hypogonadotropic Hypogonadism in Morbidly Obese Males Is Reversed after Bariatric Surgery. Obes. Surg..

[450] Zhu C., Zhang Y., Zhang L., Gao J., Mei F., Zhu B., Lu L., Zhou D., Qu S. (2019). Changes in Sex Hormones After Laparoscopic Sleeve Gastrectomy in Chinese Obese Men: A 12-Month Follow-Up. Obes. Surg..

[451] Bekaert M., Van Nieuwenhove Y., Calders P., Cuvelier C.A., Batens A.-H., Kaufman J.-M., Ouwens D.M., Ruige J.B. (2015). Determinants of Testosterone Levels in Human Male Obesity. Endocrine.

[452] Michalakis K., Mintziori G., Kaprara A., Tarlatzis B.C., Goulis D.G. (2013). The Complex Interaction between Obesity, Metabolic Syndrome and Reproductive Axis: A Narrative Review. Metabolism.

[453] Corbould A., Bhathal P.S., Dixon J.B., O’Brien P.E. (2014). Interrelationships of Serum Androgens, Omental Adipose Tissue Metabolism, and Nonalcoholic Fatty Liver Disease in Obese Premenopausal Women. Metab. Syndr. Relat. Disord..

[454] Kim B.-J., Ahn S.H., Lee S.H., Hong S., Hamrick M.W., Isales C.M., Koh J.-M. (2019). Lower Hand Grip Strength in Older Adults with Non-Alcoholic Fatty Liver Disease: A Nationwide Population-Based Study. Aging.

[455] Wang Y.-M., Zhu K.-F., Zhou W.-J., Zhang Q., Deng D.-F., Yang Y.-C., Lu W.-W., Xu J., Yang Y.-M. (2021). Sarcopenia Is Associated with the Presence of Nonalcoholic Fatty Liver Disease in Zhejiang Province, China: A Cross-Sectional Observational Study. BMC Geriatr..

[456] Kazemi M., Jarrett B.Y., Parry S.A., Thalacker-Mercer A.E., Hoeger K.M., Spandorfer S.D., Lujan M.E. (2020). Osteosarcopenia in Reproductive-Aged Women with Polycystic Ovary Syndrome: A Multicenter Case-Control Study. J. Clin. Endocrinol. Metab..

[457] Appiah D., Nwabuo C.C., Ebong I.A., Wellons M.F., Winters S.J. (2021). Trends in Age at Natural Menopause and Reproductive Life Span Among US Women, 1959–2018. JAMA.

[458] Shadyab A.H., Macera C.A., Shaffer R.A., Jain S., Gallo L.C., Gass M.L.S., Waring M.E., Stefanick M.L., LaCroix A.Z. (2017). Ages at Menarche and Menopause and Reproductive Lifespan as Predictors of Exceptional Longevity in Women: The Women’s Health Initiative. Menopause.

[459] Chung L.W., Raymond G., Fox S. (1975). Role of Neonatal Androgen in the Development of Hepatic Microsomal Drug-Metabolizing Enzymes. J. Pharmacol. Exp. Ther..

[460] Collins J.M., Wang D. (2021). Co-Expression of Drug Metabolizing Cytochrome P450 Enzymes and Estrogen Receptor Alpha (ESR1) in Human Liver: Racial Differences and the Regulatory Role of ESR1. Drug. Metab. Pers. Ther..

[461] Wang D., Lu R., Rempala G., Sadee W. (2019). Ligand-Free Estrogen Receptor α (ESR1) as Master Regulator for the Expression of CYP3A4 and Other Cytochrome P450 Enzymes in the Human Liver. Mol. Pharmacol..

[462] Huang D.Q., El-Serag H.B., Loomba R. (2021). Global Epidemiology of NAFLD-Related HCC: Trends, Predictions, Risk Factors and Prevention. Nat. Rev. Gastroenterol. Hepatol..

[463] Paik J.M., Golabi P., Younossi Y., Mishra A., Younossi Z.M. (2020). Changes in the Global Burden of Chronic Liver Diseases From 2012 to 2017: The Growing Impact of NAFLD. Hepatology.

